# Realgar‐Induced CNS Toxicity: Exploring OTC‐Mediated Ornithine Regulation of ZBTB7A Inhibits Astrocyte Glycolysis Based on the Liver–Brain Axis

**DOI:** 10.1002/advs.202502591

**Published:** 2025-11-21

**Authors:** Ping Ye, Zhen Li, Huanyong Fu, Shuai Wang, Xingang Cui, Chengbo Song, Chao Ma, Hong Jiang

**Affiliations:** ^1^ Key Laboratory of Environmental Stress and Chronic Disease Control and Prevention Ministry of Education China Medical University Shenyang Liaoning 110122 China; ^2^ School of Public Health China Medical University Shenyang Liaoning 110122 China; ^3^ The Key Laboratory of Liaoning Province on Toxic and Biological Effects of Arsenic Liaoning 110122 China; ^4^ School of Public Health Mudanjiang Medical University Mudanjiang Heilongjiang 157011 China; ^5^ Shimadzu (China) Co., LTD Shenyang Branch Shenyang 110122 China

**Keywords:** astrocyte‐glycolysis, liver–brain axis, ornithine cycle, realgar/arsenic

## Abstract

Realgar, an arsenic‐containing traditional Chinese medicine, is commonly used in clinical practice. However, prolonged, excessive, or uncontrolled administration of Chinese patent medicines containing realgar can occasionally induce adverse effects. Notably, realgar‐induced central nervous system (CNS) toxicity has garnered significant attention. To elucidate the molecular mechanism underlying realgar‐induced CNS toxicity, conditional intervention animal models (*Zbtb7a* ^GfABC1D^ KD/*Otc* ^TBG^ OE/chrysophanol intervention) are established and exposed to realgar, and a C8‐D1A astrocyte cell line transfected with si‐*Zbtb7a* is established and exposed to both iAs^3+^ and ornithine. Single‐cell transcriptome sequencing, metabolomic analysis, as well as neurobehavioral, molecular biological, and histopathological experiments are performed. These results demonstrate that arsenic derived from realgar crosses the blood–brain barrier and accumulates in the frontal lobe. Within astrocytes, arsenic triggers ZBTB7A‐mediated transcriptional repression of the glycolytic genes *Aldoa*, *Ldha*, and *Pgam1*, consequently reducing lactic acid levels. This cascade of events culminates in energy deficits within the frontal lobe, promoting apoptosis and oxidative damage. These pathological changes manifest behaviorally as decreased learning and memory capacity, diminished spontaneous exploration, and the development of anxiety‐like behaviors. Furthermore, realgar inhibits hepatic ornithine transcarbamylase (OTC), disrupting the hepatic ornithine cycle. This disruption leads to ornithine accumulation, which in turn modulates the transcription factor ZBTB7A in astrocytes, indirectly exacerbating the neurotoxic effects of arsenic. In addition, chrysophanol antagonizes the toxic effects of realgar on the CNS and liver by protecting astrocyte glycolytic function and the hepatic ornithine cycle. This study provides new perspectives and targets for the prevention and treatment of realgar‐induced neurological injuries, as well as new experimental bases and theoretical guidance for the use of rhubarb and realgar in traditional Chinese medicine.

## Graphical abstract

Arsenic in realgar induces CNS toxicity through direct and indirect effects: it activates ZBTB7A in astrocytes, which in turn inhibits glycolysis and impairs mice's learning, memory, and exploration; it also suppresses OTC in the liver to block the ornithine cycle, leading to blood ornithine accumulation that crosses into the frontal lobe and further activates ZBTB7A, worsening CNS toxicity. Chrysophanol antagonizes realgar's CNS toxicity.

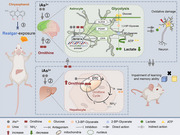

## 1 Introduction

Realgar, a mineral‐based traditional Chinese medicine (TCM) containing arsenic, has been used in China for more than 2000 years.^[^ ^1^, ^2^ ^]^ Several classic prescriptions, such as *Angong Niuhuang* pills for stroke treatment, *Niuhuang Jiedu* tablets for clearing heat and removing toxins, and Paediatric *Zhi Baodan*, all contain different doses of realgar.^[^ ^3^ ^]^ However, the persistent traditional belief that “TCM has no toxic side effects” leads some individuals to misuse realgar‐containing preparations contrary to medical advice. This misuse has resulted in occasional reports of chronic arsenic poisoning, manifesting as systemic damage to the nervous, digestive, and integumentary systems.^[^ ^4^, ^5^, ^6^ ^]^ The brain is one of the target organs of arsenic toxicity.^[^ ^7^ ^]^ Consequently, the neurotoxicity resulting from arsenic exposure and cumulative effects associated with medicinal realgar use has attracted significant public concern regarding central nervous system (CNS) impairment.

Realgar has CNS toxicity.^[^ ^8^, ^9^ ^]^ Astrocytes are the first point of entry of arsenic from realgar into the CNS.^[^ ^10^, ^11^ ^]^ These glial cells maintain robust glycolytic metabolism, supplying lactate as an energy substrate to neurons.^[^ ^11^, ^12^ ^]^ Zinc finger and BTB domain‐containing protein 7A (ZBTB7A) functions as a transcriptional regulator in reactive astrocytes, acting through sequence‐specific DNA binding domains to repress genes encoding glycolytic enzymes.^[^ ^13^, ^14^ ^]^ Consequently, whether arsenic in realgar mediates neurotoxicity via ZBTB7A‐dependent disruption of astrocytic glycolysis merits investigation.

Furthermore, our prior experiments detected elevated ornithine levels in the blood and frontal lobes of realgar‐exposed mice. Molecular docking analysis demonstrated specific binding affinity between ornithine and the ZBTB7A protein, suggesting ornithine's potential contribution to realgar‐induced CNS toxicity. Ornithine plays a key metabolite in the hepatic ornithine cycle,^[^ ^15^ ^]^ a five‐step enzymatic pathway.^[^ ^16^ ^]^ Ornithine transcarbamylase (OTC), a liver‐specific enzyme, directly catalyzes the breakdown of ornithine, and an impaired ornithine cycle caused by a deficiency in OTC results in hyperornithinemia.^[^ ^17^, ^18^ ^]^ Patients with hyperornithinemia‐hyperammonemia‐homocitrullineuria (HHH syndrome) usually present with cognitive deficits, hypotonia, and other symptoms of neurological impairment. Given realgar's established hepatotoxicity,^[^ ^19^, ^20^ ^]^ the mechanistic relationship whereby realgar‐mediated inhibition of hepatic OTC causes ornithine accumulation, potentially activating ZBTB7A‐mediated neurotoxicity, warrants further mechanistic investigation.

This study investigated the CNS and hepatotoxic effects of arsenic in realgar arsenic using in vivo and in vitro models. Building upon the liver–brain axis framework, we further examined how hepatic ornithine transcarbamylase (OTC) regulates ornithine levels and subsequently mediates ZBTB7A‐induced inhibition of astrocytic glycolysis, thereby exacerbating realgar‐associated neurotoxicity. In addition, compounding is one of the most important ways to address the toxicity of toxic Chinese medicines,^[^ ^21^ ^]^ and in the clinic, realgar is often paired with rhubarb.^[^ ^22^, ^23^ ^]^ Therefore, on the basis of the theory of reducing the toxicity of traditional Chinese medicine, we investigated the antagonistic effects of chrysophanol, the main pharmacodynamic constituent of rhubarb, on the toxic effects of realgar on the CNS and liver. This study provides a theoretical basis for understanding the mechanism underlying the compounding and toxicity reduction effects of rhubarb and realgar and provides a scientific basis and experimental foundation for the prevention and control of arsenic neurotoxicity and hepatotoxicity in realgar.

## 2 Results

### 2.1 Realgar Causes Central Nervous System (CNS) Toxicity

**Figure 1:**
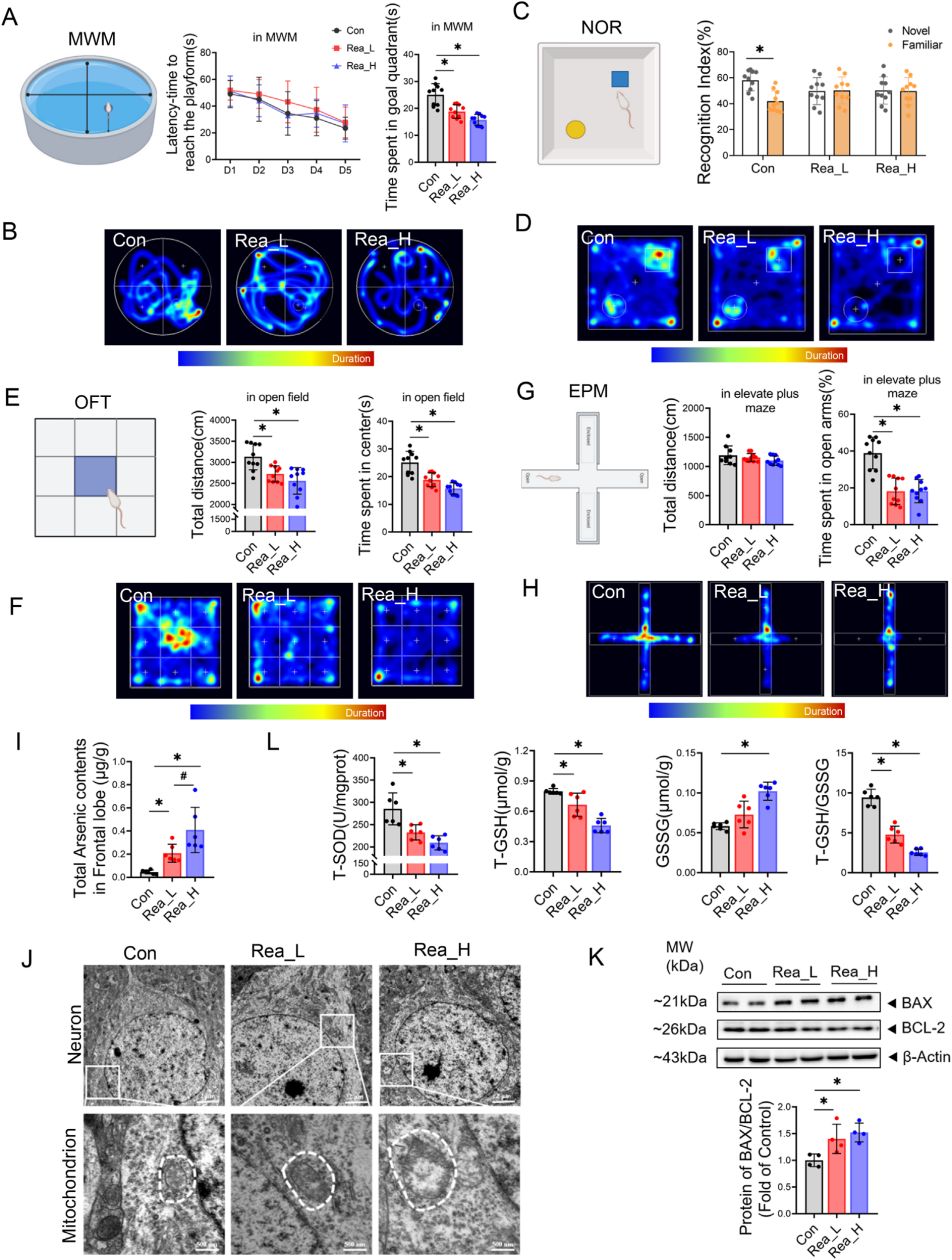
Realgar causes CNS toxicity. A) Mirros water maze, a localized navigation experiment to escape latency and space exploration experiment in platform quadrant exploration time (*n* = 10). B) Mirros water maze, mouse movement trajectory. C) Novel object recognition, cognitive index during the experimental phase (*n* = 10). D) Novel object recognition, mouse movement trajectories. E) Open field test, total distance moved and time spent in the center area by mice (*n* = 10). F) Open field test, mouse movement trajectories. G) Elevated plus maze, total distance travelled and time spent in the open arm by mice (*n* = 10). H) Elevated plus maze, mouse movement trajectories. I) Total arsenic content in the frontal lobe of mice (*n* = 6). J) Ultrastructure of mouse frontal neurons. K) Immunoblotting and quantitative analysis of the expression of BAX/BCL2 in the frontal lobe (*n* = 4). L) Mouse frontal lobe T‐SOD viability, T‐GSH, GSSG content, and T‐GSH/GSSG ratio (*n* = 6). Compared with the Con group, *p < 0.05; compared with the Rea_H group, #*p* < 0.05; the data are expressed as the mean ± SD.

To investigate the mechanism underlying the CNS toxicity caused by realgar, neurobehavioral experiments were first conducted in mice. The results showed that compared with Con group, mice exposed to realgar for 12 weeks exhibited dose‐dependent reductions in spatial learning performance as evidenced by significantly decreased cumulative residence time in the Morris water maze target quadrant(**Figure** **1**A,B, *p *< 0.05). Moreover, in the Con group, the time spent exploring the novel objects was significantly greater than that spent exploring the familiar objects (Figure 1C,D, *p *< 0.05), and the time spent exploring the familiar and novel objects in the Rea_L and Rea_H groups was not significantly different. Open field testing demonstrated progressive reductions in total ambulatory distance and central zone occupancy time with increasing realgar doses (Figure 1E,F, *p *< 0.05). Similarly, elevated plus maze analysis showed realgar dose‐progressive decreases in open arm retention time (Figure 1G,H, *p *< 0.05). These results confirmed that realgar induced a decrease in learning memory and spontaneous exploration ability and an increase in anxiety‐like behaviors in the mice.

The ICP‒MS results revealed that the total arsenic content in the brain tissues of the Rea_L and Rea_H groups gradually increased compared with the Con group (Figure 1I, *p *< 0.05), and a dose‒effect relationship was observed. This progressive deposition pattern indicates efficient blood–brain barrier penetration of realgar‐derived arsenic. Transmission electron microscopy revealed that the cell membrane and nuclear membrane of the neurons in the Con group were smooth and intact, the nuclei were large and rounded, the mitochondria were round or oval, and the mitochondrial cristae and membrane structure were clearly visible. The nuclear membrane of the neurons in the Rea_L group was somewhat fuzzy, and the mitochondrial cristae were reduced and exhibited a disordered arrangement. The neurons in the Rea_H group had unclear nuclear membrane residence, the mitochondria were swollen and lysed, and the mitochondrial cristae were reduced and even exhibited vacuole‐like degeneration (Figure 1J). In addition, compared with the Con group, the levels of the apoptosis‐related proteins BAX/BCL‐2 ratio in the frontal lobe were significantly elevated with increasing doses of realgar (Figure 1K, *p *< 0.05); moreover, the levels of the antioxidant enzymes T‐SOD and T‐GSH were significantly reduced (Figure 1L, *p *< 0.05). These findings confirmed that realgar has CNS toxicity.

### 2.2 Realgar Inhibits Astrocyte Glycolysis and Promotes Astrocyte Polarization toward the A1 Phenotype

**Figure 2:**
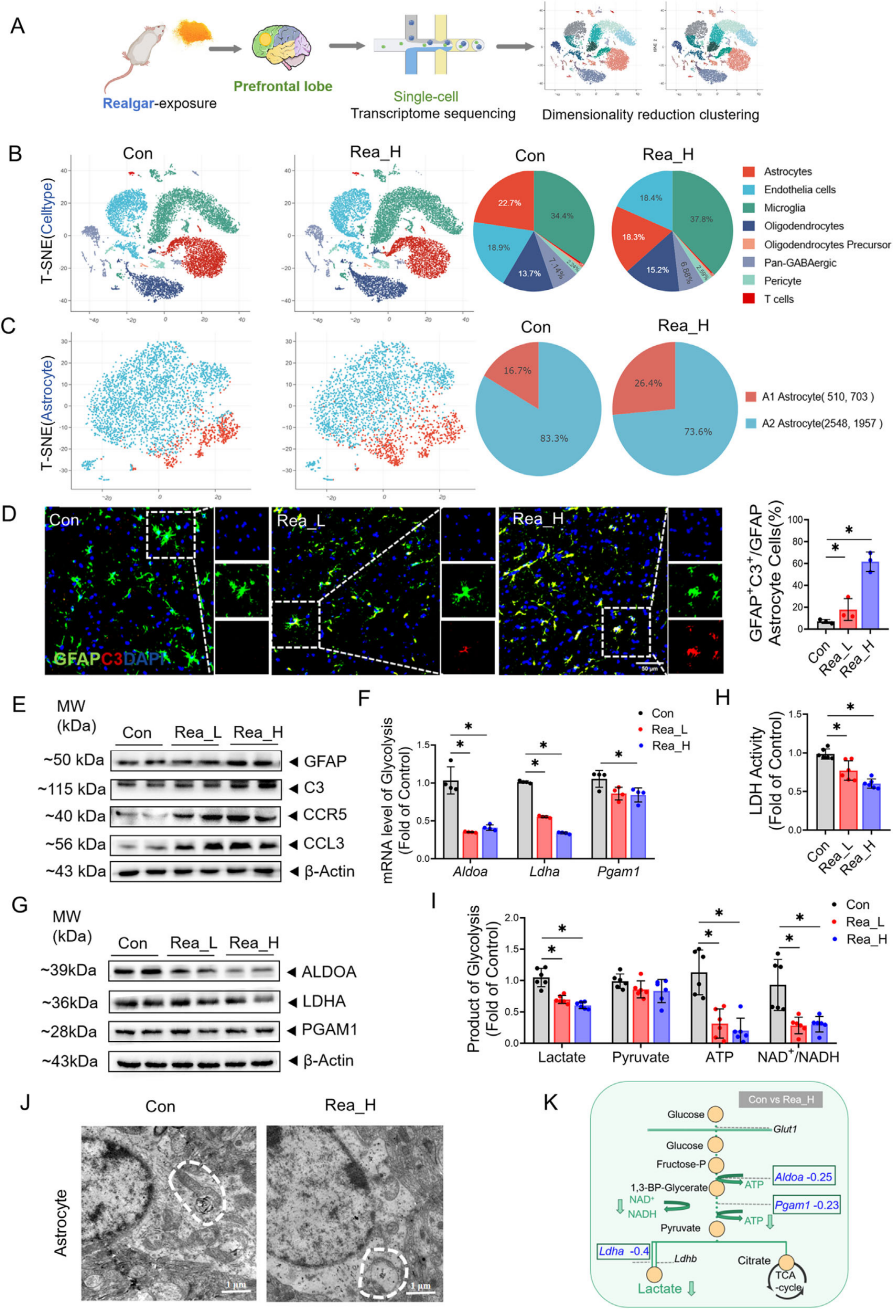
Realgar inhibits astrocyte glycolysis and promotes astrocyte polarization toward the A1 phenotype. A) Flow chart of single‐cell sequencing in the mouse frontal lobe. B) Clustering of T‐SNE down‐regulation and the percentage of number of each cell type. C) T‐SNE down‐regression clustering and number of astrocytes by two subtypes of astrocytes. D) Double immunofluorescence staining and quantitative analysis of GFAP (green) with C3 (red) in the mouse frontal lobe (*n* = 3). DAPI (nucleus), scale bar, 50 µm. E) Immunoblotting of the expression of GFAP, C3, CCR5, CCL3 in the frontal lobe (*n* = 4). F) *Aldoa*, *Ldha*, *Pgam1* mRNA expression levels in the frontal lobe (*n* = 4). G) Immunoblotting of the expression of ALDOA, LDHA, PGAM1 in the frontal lobe (*n* = 4). H) LDH viability in the frontal lobe (*n* = 6); I) Frontal lobe lactate, pyruvate, ATP content, and NAD^+^/NADH ratio (*n* = 6). J) Ultrastructure of mouse frontal astrocyte. K) Glycolysis process in frontal astrocytes in realgar‐exposed mice. Compared with the Con group, *p < 0.05; the data are expressed as the mean±SD.

Single‐cell RNA sequencing (scRNA‐seq) enables high‐resolution characterization of phenotypic and transcriptional dynamics in individual cells, providing transformative insights into neuroscience research.^[^ ^24^, ^25^ ^]^ In this study, the single‐cell suspension and library were subjected to quality control, and the single‐cell capture rate, the number of genes expressed, and the sequencing depth of the two samples met the requirements; moreover, the sequencing data were accurate and reliable (Figures S5A,B, Tables S8 and S9, Supporting Information). The two samples were clustered into 30 cell clusters by T‐SNE dimensionality reduction (**Figure** **2**A; Figure S6A,B, Supporting Information), and these clusters were defined as 7 cell types, namely, Pan‐GABAergic Neuron, Astrocyte, Microglia, Oligodendrocyte, Oligodendrocyte Precursor Cell, Endothelial Cell, T cell, and Pericyte; of these cell types, Astrocyte, Microglia, and Oligodendrocyte accounted for the majority (Figure 2B). Astrocyte subclustering resolved two functionally polarized states defined by marker gene signatures: the A1‐phenotype (neurotoxic) and A2‐phenotype (neuroprotective, Figure S6C, Supporting Information); an increased number of A1‐phenotype cells and a decreased number of A2‐phenotype cells were observed in the Rea_H group (Figure 2C). Immunofluorescence (IF) and Western blotting results revealed that, compared with those in the Con group, the numbers of fluorescence‐positive cells that were colabelled with the frontal A1‐phenotype markers C3 and GFAP were increased (Figure 2D, *p *< 0.05), and the protein expression levels of GFAP, C3, and the chemokines CCR5 and CCL3 were significantly increased (Figure 2E, Figure S6D, *p *< 0.05, Supporting Information). These integrated analyses demonstrate realgar‐induced pathogenic astrocyte polarization towards neurotoxic A1 phenotype.

For the biological function of “powerful glycolysis” in astrocytes,^[^ ^26^ ^]^ we extracted glycolysis‐related genes for analysis. And notably, compared with those in the Con group, the expression levels of *Ldha*, *Aldoa*, *Aldoc*, *Pgam1*, *Mdh1*, *Mpc1*, *Sdhb*, and *Gapdh* were significantly downregulated in the Rea_H group (Figure S6E, Supporting Information). *Aldoa*, *Ldha*, and *Pgam1* are pivotal genes in glycolysis, and their expression levels were verified by RT‒qPCR and Western (Figure 2F,G, Figure S6F, *p *< 0.05, Supporting Information). Furthermore, LDH viability decreased significantly with increasing doses of realgar (Figure 2H, *p *< 0.05). Frontal lobe metabolite analysis revealed unaltered pyruvate levels, alongside a significant reduction in lactate content, ATP levels, and the NAD⁺/NADH ratio (Figure 2I,K, *p *< 0.05). In addition, transmission electron microscopy revealed that the astrocyte mitochondria in the Con group were round or elliptical and that the mitochondrial cristae were neatly arranged, whereas the astrocyte mitochondria in the Rea_H group were swollen, the number of mitochondrial cristae was reduced, or the vacuoles were denatured (Figure 2J). These results suggested that realgar damaged the structure of astrocyte mitochondria, inhibited glycolysis, and promoted the polarization of astrocytes towards the A1 phenotype, which in turn led to frontal lobe energy defects.

### 2.3 ZBTB7A Targets and Inhibits Astrocyte Glycolysis During Realgar‐Induced CNS Toxicity

**Figure 3:**
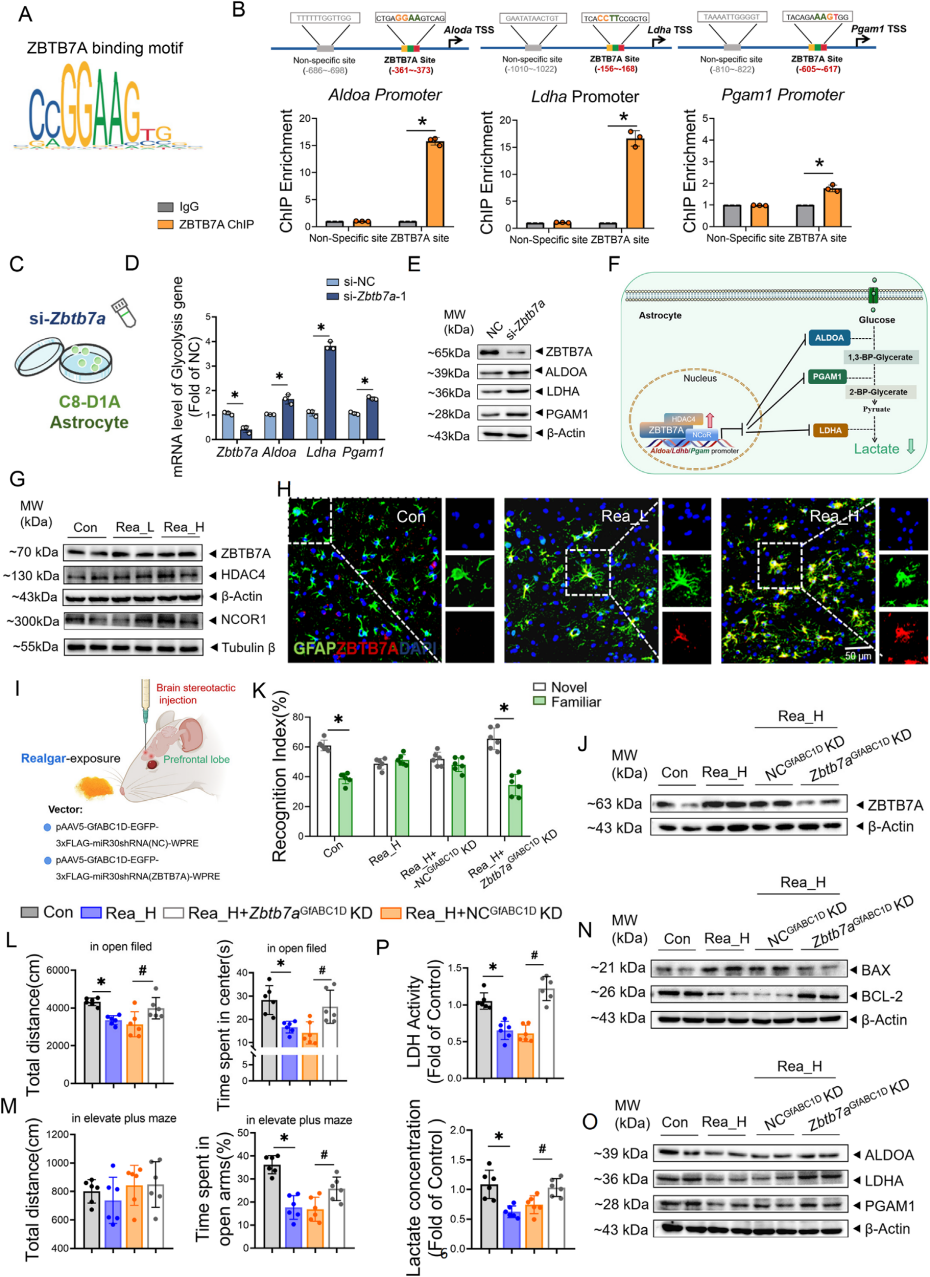
ZBTB7A targets and inhibits astrocyte glycolysis during realgar‐induced CNS toxicity. A) ZBTB7A motif map. B) ChIP assay, ZBTB7A protein was immuno‐enriched folds in *Aldoa*, *Ldha*, *Pgam1* promoters (*n* = 3). Compared with the IgG group, *p < 0.05. C) Schematic diagram of cell treatment. D) Expression levels of *Zbtb7a*, *Aldoa*, *Ldha*, *Pgam1*mRNA in C8‐D1A cells (*n* = 3). Compared with the NC group, *p < 0.05. E) Immunoblotting of the expression of ZBTB7A, ALDOA, LDHA, PGAM1 in C8‐D1A cells (*n* = 3). F) Schematic diagram of the targeting effect of ZBTB7A with *Aldoa, Ldha, Pgam1*. G) Immunoblotting of the expression of ZBTB7A, HDAC4, NCoR1 in the frontal lobe (*n* = 4). H) Double immunofluorescence staining and quantitative analysis of GFAP (green) with ZBTB7A (red) in the mouse frontal lobe (*n* = 3). DAPI (nucleus), scale bar, 50 µm. I) Schematic diagram of Zbtb7a^GfABC1D^ KD animal model construction. J) Immunoblotting of the expression of ZBTB7A in the frontal lobe (*n* = 4). K) Novel object recognition, cognitive index during the experimental phase (*n* = 6). L) Open field test, total distance moved and time spent in the center area by mice (*n* = 6). M) Elevated plus maze, total distance travelled and time spent in the open arm by mice (*n* = 6). N) Immunoblotting of the expression of BAX, BCL2 in the frontal lobe (*n* = 4). O) Immunoblotting of the expression of ALDOA, LDHA, PGAM1 in the frontal lobe (*n* = 4). *p*) LDH viability and lactate content in the frontal lobe (*n* = 6). Compared with the Con group, *p<0.05; compared with the Rea_H+NC^GfABC1D^ KD group, #*p*<0.05; the data are expressed as the mean±SD.

The mechanisms underlying the transcriptional downregulation of glycolytic enzymes *Ldha*, *Aldoa*, and *Pgam1* became the focus and challenge for subsequent investigation. Transcription factor analysis predicted ZBTB7A (motif: 5’‐CCGGAAGTG‐3’; Matrix ID MA0750.2) was found to have specific binding sites with the promoter regions of *Aldoa*, *Ldha*, and *Pgam1* (**Figure** **3**A; Figure S7A, Supporting Information). The ChIP‒qPCR demonstrated that significant ZBTB7A enrichment at specific promoter coordinates: *Aldoa* (−361 to −373 bp), *Ldha* (−156 to −168 bp), and *Pgam1* (−605 to −617 bp) in C8‐D1A cells (Figure 3B, *p *< 0.05). In vitro, the silencing of *Zbtb7a* in C8‐D1A cells was accompanied by elevated mRNA and protein levels of *Aldoa*, *Ldha*, and *Pgam1* (Figure 3C–E; Figure S7B,C, *p *< 0.05, Supporting Information). These results indicate that ZBTB7A binds specifically to the *Ldha*, *Aldoa*, and *Pgam1* promoter sequences and negatively regulates their transcription (Figure 3F).

In vivo, we assessed whether ZBTB7A was highly expressed in astrocytes after realgar exposure and found that the protein expression levels of ZBTB7A and its codeterminant factors HDAC4 and NCoR1 in the frontal lobe were significantly increased with increasing doses of realgar (Figure 3G; Figure S7D, *p *< 0.05, Supporting Information). Furthermore, nuclear translocation of ZBTB7A was significantly enhanced in astrocytes (Figure 3H). An astrocyte‐specific *Zbtb7a* knockdown model(referred to as *Zbtb7a* ^GfABC1D^ KD) was subsequently established and exposed to realgar (Figure 3I,J; Figure S7E,F, *p *< 0.05, Supporting Information). The neurobehavioral experiments revealed that realgar exposure affected the normal neurobehavioral performance of the mice, whereas the mice in the Rea_H+*Zbtb7a* ^GfABC1D^ KD group spent significantly more time exploring the novel objects than the familiar objects (Figure 3K, Figure S7G, *p *< 0.05, Supporting Information); the total distance travelled and the cumulative time spent in the central area were significantly increased in the open field test (Figure 3L; Figure S7G, *p *< 0.05, Supporting Information), and significantly more time was spent in the open arm of the elevated plus maze (Figure 3M, Figure S7G, *p *< 0.05, Supporting Information). In addition, compared with the Rea_H+NC^GfABC1D^ KD group, the levels of the BAX/BCL2 ratios were significantly lower in the frontal lobes of the Rea_H+*Zbtb7a* ^GfABC1D^ KD group (Figure 3N; Figure S7H, *p *< 0.05, Supporting Information), whereas the protein expression levels of ALDOA, LDHA, and PGAM1 were significantly greater (Figure 3O; Figure S7I, *p *< 0.05, Supporting Information), and the lactate content and LDH viability were also significantly greater (Figure 3P, *p *< 0.05). These findings suggested that astrocyte‐specific knockdown of *Zbtb7a* ameliorated realgar‐induced astrocyte glycolysis dysfunction, alleviated frontal energy deficits, and attenuated realgar‐induced neurotoxicity. On the basis of the above experiments, ZBTB7A‐targeted inhibition of astrocyte glycolysis was confirmed to be involved in realgar‐induced CNS toxicity.

### 2.4 Inhibition of Hepatic OTC Expression by Realgar Results in Ornithine Accumulation in the Liver, Blood, and Frontal Lobe

**Figure 4:**
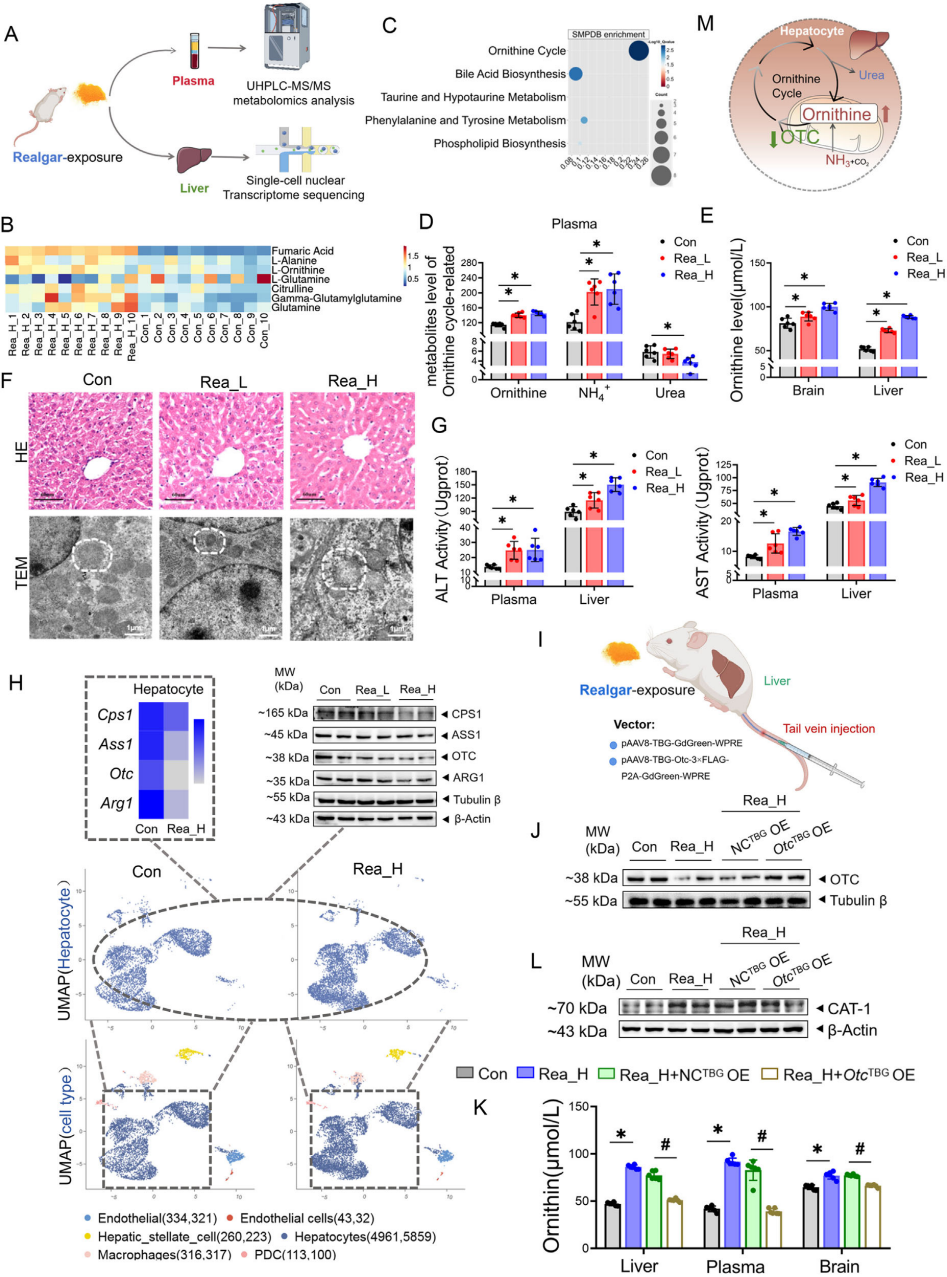
Inhibition of hepatic OTC expression by realgar results in ornithine accumulation in the liver, blood, and frontal lobe. A) Flow chart of metabolomics (plasma) and single‐cell nuclear transcriptomics (liver) sequencing in mice. B) Heatmap of ornithine cycle‐related metabolite expression (*n* = 10). C) Differential metabolite SMPDB enrichment analysis. D) Plasma levels of ornithine, ammonia, and urea (*n* = 6). E) Ornithine content in the frontal lobe and liver (*n* = 6). F) HE staining and hepatocyte ultrastructure of the mouse liver. scale bar, 60 µm, 1 µm. G) AST and ALT viability in plasma and liver (*n* = 6). H) Liver single‐cell nuclear transcriptome sequencing, ornithine cyclase mRNA and protein expression levels (*n* = 4). I) Schematic diagram of *Otc* ^TBG^ OE animal model construction. J) Immunoblotting of the expression of OTC‐1 in the liver (*n* = 4). K) Ornithine content in the liver, plasma, and brain of mice (*n* = 6). L) Immunoblotting of the expression of CAT‐1 in the frontal lobe (*n* = 4). compared with the Con group, *p < 0.05; Compared with the Rea_H+NC^TBG^ OE group, #*p* < 0.05; the data are expressed as the mean±SD.

Liquid chromatography tandem mass spectrometry (LC‒MS) metabolomics detected plasma metabolite alterations in realgar‐exposed mice(**Figure** **4**A; Figure S8A, Supporting Information). The ornithine cycle exhibited the most significant enrichment among differentially abundant metabolites, with elevated ornithine, citrulline, fumarate, and glutamine levels (Figure 4B,C). The levels of key metabolites of the ornithine cycle were verified by a kit; the contents of ornithine and ammonia gradually increased, and the plasma level of urea gradually decreased in the mice exposed to increasing doses of realgar (Figure 4D, *p *< 0.05). Ornithine plays a central role in the ornithine cycle. Notably, the ornithine content and the mRNA and protein expression of the ornithine transporter CAT‐1 were significantly elevated in the frontal lobes of realgar‐exposed mice (Figure 4E; Figure S8B, *p *< 0.05, Supporting Information). The ornithine cycle occurs primarily in mammalian livers, and we similarly observed significantly elevated levels of ornithine in the livers of realgar‐exposed mice (Figure 4E). These results suggest that realgar may damage the liver and induce ornithine cycle disorders, which can cause elevated levels of ornithine in the liver, blood, and frontal lobe.

The liver is the main target organ for the metabolism of arsenic in realgar. The ICP‒MS results revealed that the total arsenic content in the liver was significantly greater in the Rea_L and Rea_H groups than in the Con group (Figure S8C, *p *< 0.05, Supporting Information). HE staining revealed no obvious pathological changes in the Con group; however, in the Rea_H group, the hepatocyte cords were more disorganized, and the hepatic blood sinusoidal gap was widened (Figure 4F). Transmission electron microscopy revealed that the mitochondrial structure was intact in the hepatocytes of the Con group; the number of mitochondrial cristae in the Rea_H group was reduced, the mitochondria were disorganized, and vacuole‐like degeneration was even observed (Figure 4F). Dose‐dependent increases in plasma and hepatic ALT/AST activities further confirmed liver damage in reaglar (Figure 4G, *p *< 0.05). Hepatocytes are the main component unit of the liver. When the snRNA‐seq dataset was integrated, the proportion of hepatocytes in both samples exceeded 80% of the total number of cells (Figure S8D,E, Supporting Information). Notably, ornithine cycle rate‐limiting enzyme genes (*Cps1*, *Ass1*, *Otc*, *Arg1*) were significantly downregulated in hepatocytes of Rea_H group, with *Otc* being the most significantly reduced (Figure 4H, *p *< 0.05), consistent with protein and enzymatic validations (Figure S8F–H, Supporting Information). These findings suggest that OTC is a key enzyme in the impaired ornithine cycle in the livers of mice after exposure to realgar.

To verify the key role of OTC in realgar‐induced ornithine cycle disorders in the liver, a mouse model of hepatocyte‐specific overexpression of *Otc* (referred to as *Otc* ^TBG^ OE) was established and exposed to realgar (Figure 4I,J; Figure S9A, *p *< 0.05, Supporting Information). The results revealed that OTC protein expression was significantly than in the Rea_H+*Otc* ^TBG^ OE group (Figure S9B, *p *< 0.05, Supporting Information), which was accompanied by a significant decrease in ornithine levels in the liver, plasma and frontal lobe (Figure 4K, *p *< 0.05); additionally, the plasma ammonia level was decreased, and the expression of the CAT‐1 protein in the frontal lobe was also reduced (Figure 4l; Figure S9C,D, *p *< 0.05, Supporting Information). These findings confirm hepatic OTC governs ornithine accumulation during realgar‐induced ornithine cycle impairment and regulates cerebral ornithine homeostasis.

### 2.5 Ornithine‐Mediated Regulation of ZBTB7A Exacerbates the Glycolysis Inhibition in Astrocytes Induced by Arsenic in Realgar and Increases CNS Toxicity

**Figure 5:**
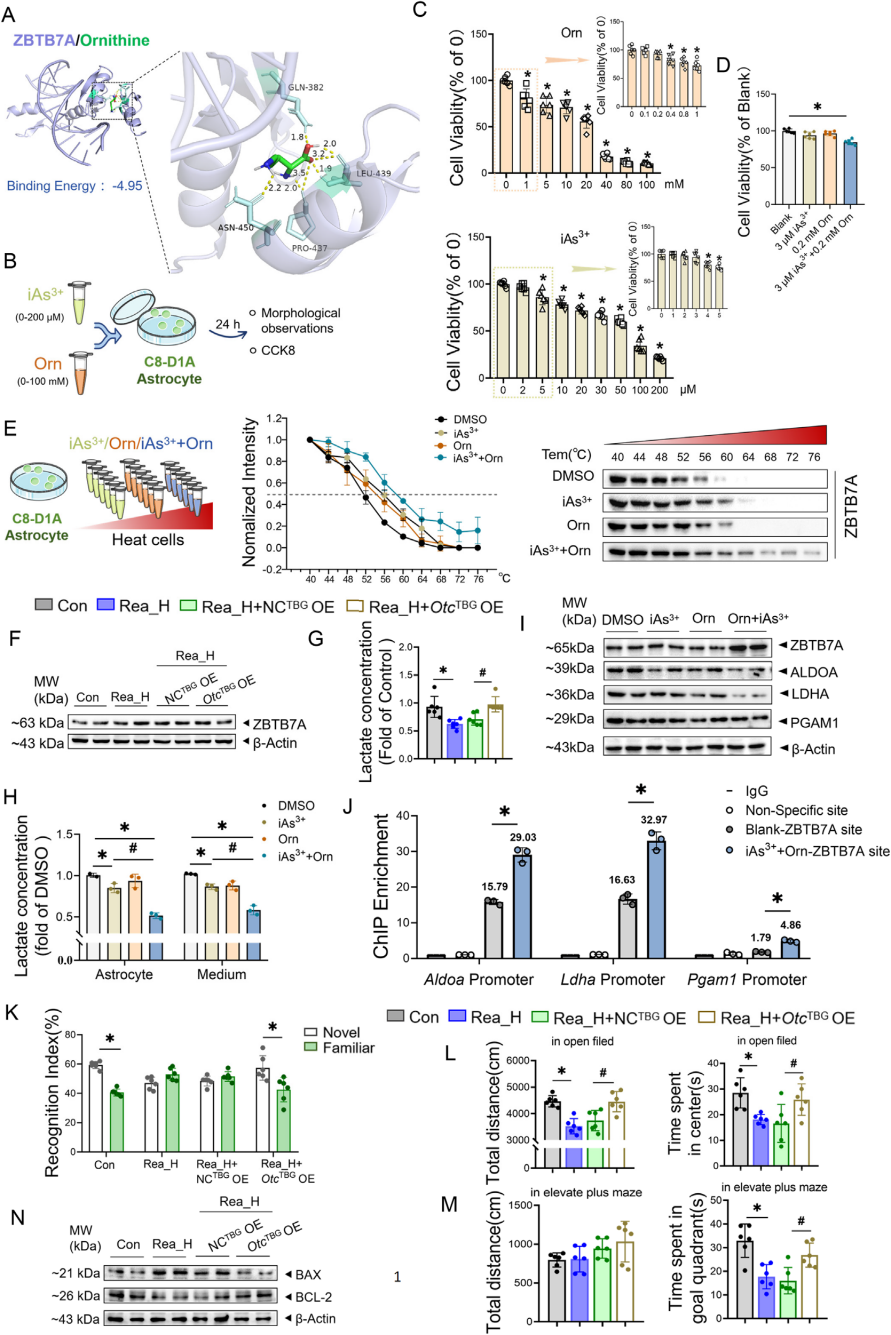
Ornithine‐mediated regulation of ZBTB7A exacerbates the glycolysis inhibition in astrocytes induced by arsenic in realgar and increases CNS toxicity. A) Visualization image of ZBTB7A protein docking with ornithine molecules. B) Schematic of cell treatment. C) Cell viability (%) after treatment of C8‐D1A with different concentrations of iAs^3+^, Orn (*n* = 6). compared with the 0 group, *p < 0.05. D) Cell viability (%) after treatment of C8‐D1A with 3 µm iAs^3+^, 0.2 mm Orn individually and in combination. Compared with the Blank group, *p < 0.05. E) Cell thermal displacement assay, immunoblotting plots, and melting curves of ZBTB7A protein degradation at 40–76°C (*n* = 3). F) Immunoblotting of the expression of ZBTB7A in the frontal lobe (*n* = 4). G) Lactate content in the frontal lobe (*n* = 6). Compared with the Con group, *p < 0.05; compared with the Rea_H+NC^TBG^ OE group, #*p* < 0.05. H) Lactate content in the C8‐D1A cells and culture medium (*n* = 3). I) Immunoblotting of the expression of ZBTB7A, ALDOA, LDHA, PGAM1 in the C8‐D1A cells (*n* = 3). Compared with the DMSO group, *p < 0.05; compared with the iAs^3+^+Orn group, #*p* < 0.05. J) ChIP assay, ZBTB7A protein was immuno‐enriched folds in *Aldoa, Ldha, Pgam1* promoters (*n* = 3). Compared with the Blank‐ZBTB7A site group, *p < 0.05. K) Novel object recognition, cognitive index during the experimental phase (*n* = 6). L) Open field test, total distance moved and time spent in the center area by mice (*n* = 6). M) Elevated plus maze, total distance travelled and time spent in the open arm by mice (*n* = 6). N) Immunoblotting of the expression of BAX, BCL2 in the frontal lobe (*n* = 4). Compared with the Con group, *p < 0.05; compared with the Rea_H+NC^TBG^ OE group, the data are expressed as the mean±SD.

The regulatory relationship between ornithine and transcription factor ZBTB7A remains unreported. Molecular docking revealed high‐affinity binding between ornithine and ZBTB7A (**Figure** **5**A). In vitro, an iAs^3^⁺ exposure model with ornithine supplementation (Orn) was established to simulate realgar‐exposed astrocyte microenvironments (Figure 5B). After microscopic observation of the morphology and CCK8 experiments, 3 µm iAs^3+^ with 0.2 mm Orn were chosen as the treatment conditions (Figure 5C; Figure S10A, *p *< 0.05, Supporting Information); the results revealed that exposure to 3 µm iAs^3+^ or 0.2 mm Orn alone had no effect on the viability of C8‐D1A cells, whereas the combination of the two for 24 h resulted in a significant decrease in cell viability (Figure 5D, *p *< 0.05). In the Cellular Thermal Shift Assay (CETSA), the degradation of the ZBTB7A protein significantly slowed after combined treatment with iAs^3+^ and Orn, increasing the thermal stabilization of the ZBTB7A protein (Figure 5E). Taken together, these findings indicate ZBTB7A serves as a common target for ornithine and arsenic, with ornithine potentiating arsenic‐induced cytotoxicity.

In vivo, realgar‐exposed mice presented a decrease in frontal ZBTB7A protein levels (Figure 5F, *p *< 0.05), as well as an increase in lactate content and LDH activity (Figure 5G; Figure S10B,C, *p *< 0.05, Supporting Information), after the overexpression of OTC in hepatocytes. In vitro, the iAs^3^⁺ and iAs^3^⁺+Orn groups exhibited reduced lactate (cells/medium) and cellular LDH activity versus controls in C8‐D1A cells (Figure 5H; Figure S10D, *p *< 0.05, Supporting Information). Notably, iAs^3^⁺+Orn co‐exposure further decreased these metrics versus iAs^3^⁺ alone. In addition, in both the iAs^3+^ and iAs^3+^+ Orn groups, the protein expression of ZBTB7A was significantly greater than that in the iAs^3+^+ Orn group (Figure 5I; Figure S10E, *p *< 0.05, Supporting Information), and the protein expression levels of ALDOA, LDHA, and PGAM1 were significantly lower (Figure 5I; Figure S10E, *p *< 0.05, Supporting Information). Similarly, there was a significant change in the expression levels of these proteins in the iAs^3+^+ Orn group compared with those in the iAs^3+^ group. The ChIP‒qPCR results revealed that the enrichment products of the ZBTB7A protein in the promoter regions of *Ldha* (−156 to −168 bp), *Aldoa* (−361 to −373 bp), and *Pgam1* (−605 to −617 bp) were significantly increased by combined exposure to iAs^3+^ and Orn compared with those in the untreated groups (Figure 5J). These findings suggest that the combined exposure of iAs^3+^ and Orn promotes binding between ZBTB7A and the promoters of *Aldoa*, *Ldha*, and *Pgam1*. In summary, in astrocytes, ornithine targets and regulates ZBTB7A; represses the transcription of *Ldhb*, *Aldoa*, and *Pgam*; and exacerbates the inhibition of glycolytic function that is caused by arsenic in realgar.

Given hepatic OTC regulates cerebral ornithine during realgar exposure; thus, the *Otc* ^TBG^ OE mouse model was used to explore the effect of ornithine on the toxic effects of realgar on the CNS. The neurobehavioral experiments revealed that realgar exposure affected the normal neurobehavioral performance of the mice, whereas the mice in the Rea_H+*Otc* ^TBG^ OE group spent significantly more time exploring the novel objects than the familiar ones (Figure 5K; Figure S10F, *p *< 0.05, Supporting Information); the total distance travelled and the cumulative time spent in the central area were significantly greater in the open field test (Figure 5L; Figure S10F, *p *< 0.05, Supporting Information), and significantly more time was spent in the open arm of the elevated plus maze (Figure 5M; Figure S10F, *p *< 0.05, Supporting Information). *Otc* ^TBG^ OE reversed the reduction in the T‐SOD content and T‐GSH/GSSG ratio (Figure S10H, *p *< 0.05, Supporting Information), increase in the BAX/BCL‐2 ratio in the frontal lobe caused by realgar exposure (Figure 5N; Figure S10G, *p *< 0.05, Supporting Information). These results confirm hepatocyte OTC overexpression mitigates realgar‐induced cognitive deficits and neurotoxicity, demonstrating that realgar‐induced CNS toxicity critically depends on ornithine accumulation.

### 2.6 Chrysophanol Protects Against Disorders of Astrocyte Glycolysis and the Hepatic Ornithine Cycle, Antagonizing Realgar‐Induced CNS Toxicity and Hepatotoxicity

**Figure 6:**
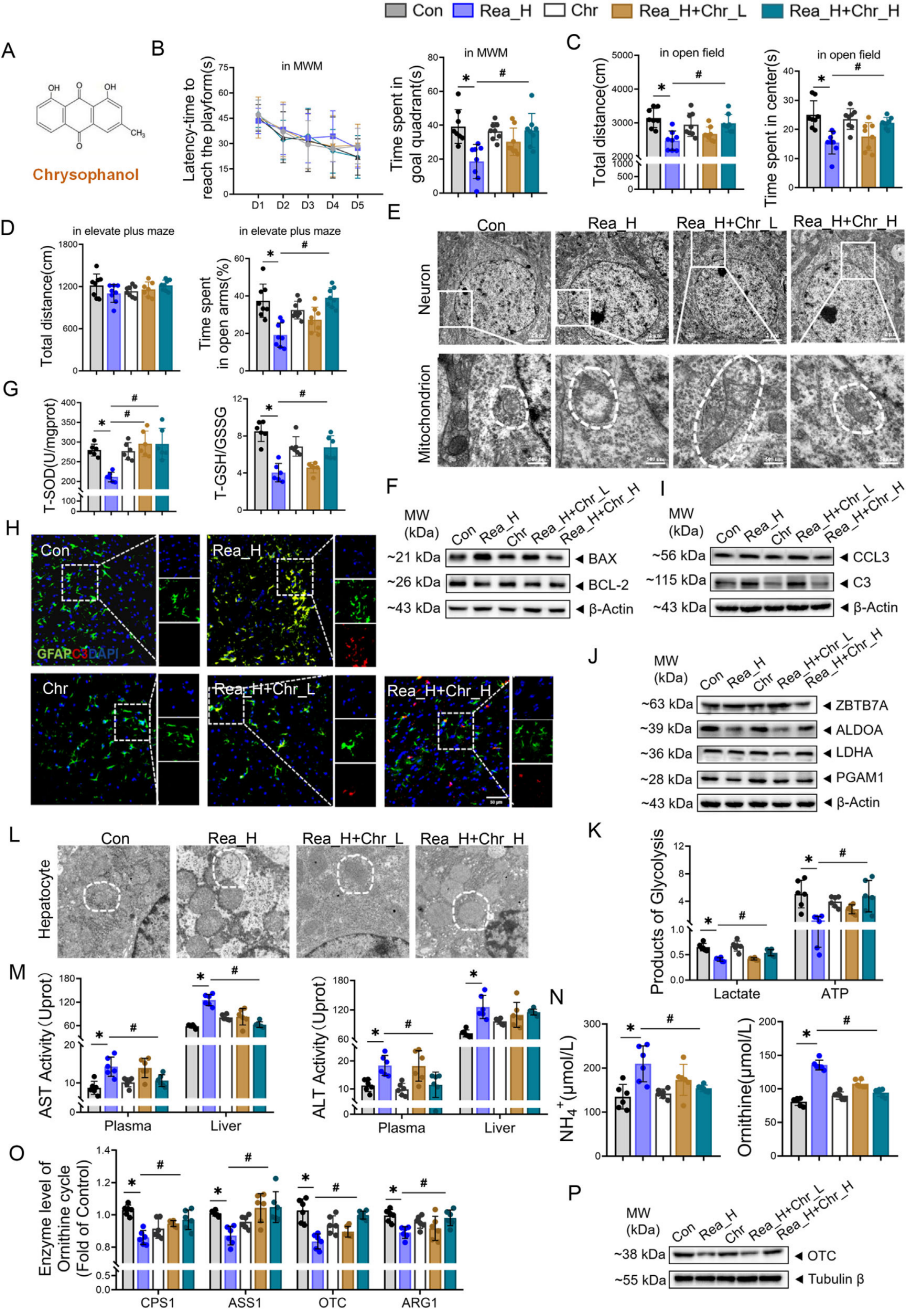
Chrysophanol protects against disorders of astrocyte glycolysis and the hepatic ornithine cycle, antagonizing realgar‐induced CNS toxicity and hepatotoxicity. A) Chemical structure of chrysophanol. B) Mirros water maze, localized navigation experiment to escape latency and space exploration experiment in platform quadrant exploration time (*n* = 8). C) Open field test, total distance moved and time spent in the center area by mice (*n* = 8). D) Elevated plus maze, total distance travelled, and time spent in the open arm by mice (*n* = 8). E) Ultrastructure of mouse frontal neurons. F) Immunoblotting of the expression of BAX, BCL2 in the frontal lobe (*n* = 4). G) Mouse frontal lobe T‐SOD viability, and T‐GSH/GSSG ratio (*n* = 6). H) Double immunofluorescence staining and quantitative analysis of GFAP (green) with C3 (red) in the mouse frontal lobe (*n* = 3). DAPI (nucleus), scale bar, 50 µm. I) Immunoblotting of the expression of C3, CCL3 in the frontal lobe (*n* = 4). J) Immunoblotting of the expression of ZBTB7A, ALDOA, LDHA, PGAM1 in the frontal lobe (*n* = 4). K) Frontal lobe lactate, and ATP content (*n* = 6). L) Ultrastructure of mouse hepatocyte. M) AST and ALT viability in plasma and liver (*n* = 6). N) Ornithine, and NH^4+^ content in plasma. O) Enzymatic activity of CPS1, ARG1, OTC, and ASS1 in the liver (*n* = 6). *p*) Immunoblotting of the expression of OTC in the liver (*n* = 4). Compared with the Con group, *p < 0.05; Compared with the Rea_H group, the data are expressed as the mean±SD.

A mouse model of realgar exposure with chrysophanol intervention was established. Neurobehavioral analyses revealed that realgar impaired normal neurobehavioral function, whereas chrysophanol significantly: increased the time spent by the mice in the target quadrant of the Morris water maze (**Figure** **6**B; Figure S11A, *p *< 0.05, Supporting Information); increased the total distance travelled and the cumulative time spent in the central area in the open field test (Figure 6C; Figure S11A, *p *< 0.05, Supporting Information); and enhanced the time spent in the open arm of the elevated plus maze (Figure 6D; Figure S11A, *p *< 0.05, Supporting Information). Moreover, transmission electron microscopy revealed that compared with those in the Rea_H group, the cell membrane and nuclear membrane structures of neurons in the Rea_H+Chr_L and Rea_H+Chr_H groups were smoother and more intact, and the mitochondrial cristae were neatly arranged without swelling or vacuole‐like degeneration (Figure 6E). In addition, compared with those in the Rea_H group, the levels of BAX/BCL‐2 ratios gradually decreased in the Rea_H+Chr_L and Rea_H+Chr_H groups (Figure 6F; Figure S11B, *p *< 0.05, Supporting Information), and the contents of T‐SOD and T‐GSH gradually increased (Figure 6G, *p *< 0.05). Among them, none of the above indices were significantly changed in the Chr group. The effects of chrysophanol on the astrocyte phenotype in the frontal lobe of realgar exposed mice were analyzed by IF and Western blotting. The amount of fluorescently labelled C3 and GFAP in the frontal lobe was significantly lower after chrysophanol intervention than in the Rea_H group (Figure 6H; Figure S11C, *p* < 0.05, Supporting Information), and the expression levels of the C3 and CCL3 proteins were similarly significantly lower (Figure 6I; Figure S11D,E, *p *< 0.05, Supporting Information); the expression levels of ZBTB7A proteins were similarly significantly lower and glycolysis‐related proteins ALDOA, LDHA, and PGAM1 proteins were significantly elevated (Figure 6J; Figure S11F, *p *< 0.05, Supporting Information). Moreover, chrysophanol reversed the realgar‐induced defects in the lactate and ATP contents and the NAD^+^/NADH ratio (Figure 6K; Figure S11G,H, *p *< 0.05, Supporting Information). Taken together, these results suggest that chrysophanol antagonizes realgar‐induced astrocyte glycolytic dysfunction and attenuates frontal energy deficits, thereby alleviating frontal oxidative damage and apoptosis and ameliorating realgar‐induced deficits in learning, memory, and spontaneous exploratory ability in mice.

Hepatic ultrastructural analysis showed chrysophanol restored mitochondrial integrity versus Rea_H, with well‐defined cristae and absence of vacuolar degeneration (Figure 6L). In addition, chrysophanol significantly reduced the viability of ALT and AST in the plasma and liver of realgar‐exposed mice (Figure 6M, *p *< 0.05). Similarly, none of the above indices significantly changed in the Chr group. Analysis of the ornithine cycle‐related indices revealed that, compared with the Rea_H group, chrysophanol significantly decreased the blood ammonia and ornithine levels (Figure 6N, *p *< 0.05), significantly increased the blood urea levels (Figure S11I, *p *< 0.05, Supporting Information), and significantly increased the enzyme levels of the rate‐limiting enzymes of the ornithine cycle (CPS1, ASS1, OTC, and ARG1) as well as OTC protein levels (Figure 6O,P; Figure S11J, *p *< 0.05, Supporting Information). These experiments revealed that chrysophanol ameliorated realgar‐induced hepatic ornithine cycle disorders, alleviated the high ammonia–ornithine environment in the blood circulation, and thus antagonized realgar hepatotoxicity.

## 3 Discussion

Realgar mainly contains As_2_S_2_, which is one of the 28 toxic Chinese medicines specifically prescribed by the State Council in the *Measures for the Administration of Toxic Medicines for Medical Use*.^[^ ^27^, ^28^ ^]^ The precautions in the *Chinese Pharmacopoeia* describe realgar as prudent for internal use, not to be used for a long time, and prohibited for pregnant women. Clinically, abuse of realgar or realgar‐containing preparations may induce adverse reactions including dizziness, headache, irritability, dysphagia, abdominal pain, hyperpigmentation, and palmoplantar keratosis, with neurological, gastrointestinal, and dermatological toxicity being predominant manifestations.^[^ ^4^, ^29^ ^]^ Arsenic is listed by the WHO as “one of the 10 chemicals of major public health concern”,^[^ ^30^, ^31^ ^]^ and the brain is one of the target organs of arsenic‐induced toxicity.^[^ ^7^, ^32^ ^]^ Clinical studies, epidemiological investigations and animal experiments have shown that chronic arsenic exposure can lead to language, cognitive, behavioral, hearing and motor deficits.^[^ ^32^ ^]^ The resulting effects of realgar on the human CNS have become a popular topic.

To elucidate the intrinsic molecular mechanism realgar‐induced neurotoxicity, we used female ICR mice, which were intragastrically treated with realgar for 3 months, to establish two doses (0.45 g kg^−1^ (Rea_L) and 1.35 g kg^−1^ (Rea_H)) for use in the realgar‐exposed mouse models. Neurobehavioral assessments revealed that the learning, memory, and spontaneous exploratory ability of the mice were reduced and that anxiety‐like behavior was apparent after exposure to realgar. Consistent with our team's previous studies,^[^ ^33^ ^]^ these findings are also closely related to the clinical manifestations of neurological damage, such as dizziness, headache, and amnesia, resulting from the administration of realgar‐containing herbal preparations. The frontal lobe is the main brain area responsible for higher cognitive functions such as attention, memory, and problem solving, as well as a key brain area responsible for emotion regulation such as anxiety and fear extinction.^[^ ^34^, ^35^, ^36^ ^]^ The results of ICP‒MS revealed that arsenic accumulation occurred in the frontal lobe after realgar exposure. Transmission electron microscopy revealed that the nuclear membrane boundary of frontal neurons was unclear and that the mitochondrial structure was disrupted; moreover, the degree of apoptosis in the frontal lobe was elevated, and oxidative damage occurred. In conclusion, the toxic effects of realgar on the CNS was confirmed.

The development and application of scRNA‐seq has allowed researchers to deconstruct complex neural systems, helping to identify the diversity of glial cells.^[^ ^37^ ^]^ With respect to the scRNA‐seq data, we divided the captured astrocytes into two subpopulations, A1 (neurotoxic)/A2 (neuroprotective), and found that the number of cells with the A1 phenotype increased and the number of cells with the A2 phenotype decreased in the Rea_H group. Polarization of astrocytes towards the A1 phenotype after realgar exposure was confirmed by detection of A1 phenotype markers by IF and Western blotting experiments. Astrocytes are the only glycogen‐storing cells in the brain and express high levels of the catalytic enzymes required for the glycolytic process^[^ ^38^ ^]^; thus, their main metabolic mode is glycolysis. Notably, many glycolysis‐related genes, *Ldha*, *Aldoa*, *Aldoc*, *Pgam*, *Mdh1*, *Mpc1*, *Slc2a1*, and *Gapdh*, were downregulated in the astrocytes of the Rea_H group. Among these genes, *Aldoa*, *Ldha*, and *Pgam1* are important genes for glycolysis and were experimentally verified and subsequently focused on. Moreover, the fluxes of glycolysis and energy metabolites (lactate, ATP, and NAD^+^/NADH) were significantly reduced in the frontal lobes of the Rea_H group, suggesting frontal energy defects. In addition, transmission electron microscopy revealed a certain degree of damage to the mitochondria of astrocytes in the frontal lobe of the Rea_H group. Studies have shown that impaired brain energy triggered by the absence or reduction of early glycolytic‐metabolizing enzymes accelerates AD pathology, resulting in learning memory and cognitive dysfunction.^[^ ^38^, ^39^ ^]^

Therefore, the reason for the reduced expression of glycolytic enzymes (ALDOA, LDHA, and PGAM1) in the frontal astrocytes of realgar‐exposed mice became the focus of our next study. Transcription factor prediction analysis revealed that ZBTB7A has specific binding sites (5’‐CCGGAAGTG‐3’) with the *Aldoa*, *Ldha*, and *Pgam1* promoter regions. ZBTB7A specifically binds to cis‐acting elements in the promoter region of genes through a DNA‐binding domain and synergistically represses the transcription of target genes through the recruitment of inhibitory complexes (NCoR and HDAC).^[^ ^40^, ^41^ ^]^ While Liu et al.^[^ ^14^ ^]^ reported that reduced ZBTB7A expression in colon cancer patients was positively correlated with the upregulation of the glycolytic genes *Glut3*, *Pfkp*, and *Pkm* and poor patient survival. However, we did not observe changes in the transcript levels of *Glut3*, *Pfkp* and *Pkm* in the scRNA‐seq data but did find significant reductions in the transcript levels of *Aldoa*, *Ldha*, and *Pgam1*, which may be attributed to the different glycolytic genes affected by ZBTB7A transcriptional repression due to differences in the subject population, intervention modality and disease model. In vitro, we found that ZBTB7A silencing in C8‐D1A cells elevated *Ldha*, *Aldoa*, and *Pgam1* expression. Moreover, the ChIP‒qPCR results confirmed that ZBTB7A was enriched in the promoter regions of *Aldoa*, *Ldha*, and *Pgam1*. These results suggest that ZBTB7A targets and represses the transcription of the glycolytic genes *Aldoa*, *Ldha*, and *Pgam1* in astrocytes. However, Whether realgar mediates ZBTB7A‐driven astrocyte metabolic dysregulation remains unexplored.

ZBTB7A was significantly upregulated in frontal astrocytes but unchanged in neurons and microglia of depressed mice, indicating its role as a key transcriptional regulator in reactive astrocytes.^[^ ^14^ ^]^ Additionally, we similarly observed that ZBTB7A was upregulated in the frontal astrocytes of realgar exposed mice. Next, to verify the effect of impaired glycolysis mediated by ZBTB7A in astrocytes on realgar neurotoxicity, a mouse model of astrocyte‐specific *Zbtb7a* knockdown (referred to as *Zbtb7a* ^GfABC1D^ KD) was established on the basis of exposure to realgar. The results confirmed that realgar‐induced astrocyte glycolytic dysfunction and frontal lobe energy deficits were alleviated after *Zbtb7a* ^GfABC1D^ KD; moreover, the learning memory and spontaneous exploratory ability of the mice improved, and frontal lobe apoptosis was attenuated. However, it is thus unclear whether the upstream target of ZBTB7A is directly regulated by arsenic in realgar or whether other mediators coregulate it.

Since realgar‐containing proprietary Chinese medicines are mostly administered orally, the drugs are absorbed and metabolized into the blood circulation for distribution to target organs.^[^ ^42^ ^]^ We used LC‒MS metabolomics to detect and analyze the changes in plasma metabolites in mice after realgar exposure. The differentially abundant metabolites were screened and found to be involved primarily in the biological process of ornithine cycling. The ornithine cycle is an important metabolic process for increasing the toxicity of high levels of ornithine–citrulline–ammonia in the blood.^[^ ^43^ ^]^ However, our experiments revealed that the plasma ornithine and ammonia levels were significantly greater and that the end‐product blood urea level was significantly lower in the Rea_H group. These findings suggest that the plasma ornithine cycle metabolite profile was disrupted in mice after realgar exposure. Most extrahepatic organs lack an intact ornithine cycle enzyme system, so this process is completed mainly in the liver.^[^ ^44^ ^]^ The liver, being arsenic's primary metabolic site, accumulated realgar‐derived arsenic, resulting in hepatocyte structural damage and functional impairment,^[^ ^45^ ^]^ which were similarly confirmed in our study. Any factor that may affect liver function, including stress and exogenous chemicals, may cause damage to the ornithine cycle enzyme system in liver cells.^[^ ^46^ ^]^ We detected by snRNA‐seq that the transcript levels of the ornithine cycle rate‐limiting enzymes *Cps1*, *Ass1*, *Otc*, and *Arg* were significantly reduced in the hepatocytes of the Rea_H group, and the levels of *Otc* were the most significantly reduced. Subsequent gene/protein/enzyme validations corroborated these findings. In addition, ornithine levels in the liver increased with increasing doses of realgar. Therefore, We thus propose that realgar impairs hepatic ornithine cycling‐particularly through OTC suppression‐leading to ornithine accumulation.

To test this hypothesis, a mouse model of hepatocyte‐specific overexpression of *Otc* (referred to as *Otc* ^TBG^ OE) with realgar exposure was established; we confirmed the alleviation of hepatic ornithine cycling disorders in realgar‐exposed mice after *Otc* ^TBG^ OE. Studies have shown that impaired transport, catabolism, or metabolism of ornithine into the mitochondrial matrix caused by OTC defects can lead to HHH syndrome, which is characterized by cognitive deficits, ataxia, and other neurological symptoms.^[^ ^47^, ^48^ ^]^ In the present study, we found that *Otc* ^TBG^ OE ameliorated the realgar‐induced reduction in learning memory ability and spontaneous exploration, alleviated frontal neuronal cell apoptosis and oxidative stress in mice, moreover, reduced the ornithine content and the protein expression of the ornithine transporter CAT‐1 in the frontal lobes of realgar exposed mice. These experimental results confirmed that hepatic OTC expression regulates the level of ornithine in the brain and that the neurotoxicity of realgar is closely related to its inhibition of hepatic OTC expression.

It is well known that high ammonia concentrations lead to severe damage to nerve cells.^[^ ^49^ ^]^ Clinically, patients with HHH syndrome develop progressive neurological dysfunction despite maintaining control of blood ammonia levels; therefore, acute or sustained accumulation of metabolites, mainly ornithine and homocitrulline, may often be a major factor in triggering neurological symptoms.^[^ ^50^, ^51^, ^52^ ^]^ However, stereotactic injection of citrulline into the brain did not affect oxidative stress‐related enzymes in the rat brain, whereas ornithine injection triggered lipid peroxidation damage in the brain.^[^ ^53^ ^]^ Therefore, ornithine was analyzed in depth as a key metabolite in this study. Viegas^[^ ^54^ ^]^ used ornithine to treat isolated rat cerebral cortical cells and reported a dose‐dependent decrease in the cellular utilization of glucose and acetate and a decrease in glycolysis. Moreover, Zanatta^[^ ^50^ ^]^ reported that ornithine disrupted redox and energy homeostasis in rat cortical astrocytes. This study demonstrated that realgar exposure activates the transcription factor ZBTB7A in astrocytes and inhibits astrocyte glycolytic function. Therefore, we hypothesized that ornithine may be a key metabolite involved in the astrocyte glycolytic impairment induced by arsenic in realgar. The relationship between the regulatory role of ornithine and the transcription factor ZBTB7A has not yet been studied. Our findings on the molecular docking of ornithine with the ZBTB7A protein suggest that ornithine has a regulatory effect on the ZBTB7A protein.

Therefore, in vitro models of C8‐D1A cells exposed to iAs^3+^ and ornithine (Orn) alone or in combination were established to mimic the brain microenvironment in which astrocytes are exposed to realgar. Exposure to low concentration (3 µm iAs^3+^ and 0.2 mm Orn) exposure alone had no significant effect on C8‐D1A cell morphology or viability, whereas cell viability was significantly reduced after combined exposure (3 µm iAs^3+^+0.2 mm Orn), suggesting that ornithine exacerbates arsenic‐induced cytotoxicity in C8‐D1A cells. In addition, after analysis of ALDOA, LDHA, and PGAM1 protein expression and lactate flux, along with ChIP experiments were performed, it was confirmed that ornithine synergizes with arsenic to inhibit astrocyte glycolytic function in astrocytes. Similarly, in vivo, the reduction in ornithine in the frontal lobes of realgar‐exposed *Otc* ^TBG^ OE mice was accompanied by a decrease in the level of ZBTB7A protein expression, along with an increase in the level of glycolysis. The results of these in vitro and in vivo experiments confirmed that after realgar exposure, ornithine and arsenic coregulated the transcription factor ZBTB7A in astrocytes, mainly by acting on the DNA‐binding domain of ZBTB7A, increasing the stability of the protein‒DNA complex, and synergistically promoting the binding of ZBTB7A to the promoters of *Ldhb*, *Aldoa*, and *Pgam1*, thereby repressing glycolysis.

Compounding is an important method for addressing the toxicity of toxic Chinese medicines and can increase the rationality and safety of the clinical application of toxic Chinese medicines.^[^ ^55^ ^]^ Realgar is commonly used in combination with rhubarb, in preparations such as *Niuhuang Jiedu tablets*, *Xiaoer Qingre tablets*, *Xiaoer Huadu San* and other daily medicines.^[^ ^56^, ^57^, ^58^ ^]^ Studies have confirmed that the total anthraquinone of rhubarb is one of the main chemical constituents groups that reduces the toxicity of realgar in *Niuhuang Jiedu tablets*.^[^ ^59^ ^]^ Chrysophanol is 1,8‐dihydroxy‐3‐methyl anthraquinone (C_15_H_10_O_4_), which accounts for approximately 1/2 of total rhubarb anthraquinone, and has pharmacological properties such as anti‐inflammatory effects, improved memory function, and anti‐myocardial ischaemia effects.^[^ ^59^, ^60^ ^]^ Therefore, in this study, the dose of chrysophanol used as an entry point was selected on the basis of the *Chinese Pharmacopoeia* (2020)^[^ ^61^ ^]^ “relevant provisions for under the *Niuhuang Jiedu tablets*”, with 0.04 g kg^−1^ chrysophanol as the low‐dose intervention group and two times the amount of low‐dose chrysophanol, which represents the amount of total anthraquinones, as the high‐dose intervention group (0.08 g kg^−1^). A chrysophanol intervention model was established based on a previously established realgar‐treated mouse model to further explore the antagonistic effects of rhubarb on realgar toxicity.

Studies have shown that chrysophanol pretreatment significantly restores neurological function in a mouse model of cerebral ischaemia‒reperfusion.^[^ ^62^, ^63^ ^]^ In this study, we confirmed that chrysophanol could alleviate frontal lobe energy deficits by ameliorating realgar‐induced astrocyte glycolytic dysfunction; reversing realgar‐induced frontal lobe neuronal structural damage, apoptosis and oxidative stress; and ultimately ameliorating abnormal neurobehavioral activities, such as decreased learning memory, reduced spontaneous exploration, and increased anxiety‐like behaviors, in mice. The total anthraquinones of rhubarb are metabolized mainly in the liver, and chrysophanol is one of their metabolites.^[^ ^64^, ^65^ ^]^ In the present study, chrysophanol ameliorated realgar‐induced hepatic ornithine cycle disturbances, alleviated the hyperammonia‒hyperornithine environment in the circulation, and improved liver function. Furthermore, chrysophanol ameliorated ammonia‐induced astrocyte damage by preventing oxidative stress.^[^ ^66^ ^]^ We therefore speculate that chrysophanol may indirectly antagonize realgar neurotoxicity by antagonizing the relatively induced inhibition of hepatic OTC expression, ameliorating ornithine cycle disorders, and thereby regulating ZBTB7A‐mediated regulation of astrocyte glycolysis; however, experimental confirmation is needed.

In summary, on the basis of the liver‒brain axis theory, our study investigated the molecular mechanisms of direct and indirect regulatory effects of realgar‐induced neurotoxicity in depth from the perspective of multiorgan and multicellular interactions with ornithine as a central mediator. The specific mechanisms were as follows: After exposure to realgar, the transcriptional repression of *Aldoa*, *Ldha*, and *Pgam1* by the transcription factor ZBTB7A in astrocytes led to astrocytic glycolytic dysfunction, resulting in frontal lobe energy deficits, which ultimately resulted in neurotoxicity manifestations such as neuronal structural damage, reduced antioxidant capacity and increased apoptosis levels, and reduced learning and memory and spontaneous exploration ability in mice. Concurrently, realgar suppressed hepatic OTC expression, leading to ornithine cycle disorders manifested as ornithine accumulation in the blood. Ornithine and arsenic co‐regulated the transcription factor ZBTB7A in astrocytes, synergistically enhancing its transcriptional repression of glycolytic genes and exacerbating neurotoxicity. Furthermore, the antagonistic effects of chrysophanol on the hepatotoxicity and neurotoxicity caused by realgar were elucidated on the basis of the theory of toxicity reduction in Chinese herbal medicine. However, there are still several limitations in this study: 1. Following realgar exposure, how arsenic or ornithine modulates ZBTB7A‒including whether it involves RNA modifications or protein covalent modifications‒remains to be elucidated. 2. While we have confirmed ornithine as a mediator in liver‒brain axis communication, other undiscovered mediators may contribute to realgar's indirect neurotoxicity. 3. Chrysophanol's detoxification mechanisms‐whether direct (e.g., altering realgar absorption, distribution, metabolism, or excretion) or indirect (specific target modulation)‒require further investigation. In conclusion, this work identifies novel therapeutic targets for preventing realgar‐induced neurological injury and provides an experimental foundation for optimizing rhubarb‐realgar formulations in clinical practice.

## 4 Conclusion

Arsenic in realgar induces CNS toxicity through direct and indirect effects. The specific mechanism involves the activation of transcriptional repression of *Aldo*a, *Ldha*, and *Pgam1* by ZBTB7A in astrocytes after exposure to arsenic in realgar, which in turn inhibits astrocyte glycolysis, ultimately leading to decreases in mice's learning and memory abilities and spontaneous exploration; in addition, realgar impairs hepatic function, inhibits hepatic OTC, induces ornithine cycle blockage, and leads to ornithine that accumulation in the blood and crosses into the frontal lobe, which further activates ZBTB7A and exacerbates the CNS toxicity of arsenic in realgar. In addition, chrysophanol can antagonize the CNS toxicity of realgar.

## 5 Experimental Section

### Reagents and Chemicals

Realgar (>90% As_2_S_2_) was purchased from Henan Sanmenxia Yuhuangshan Pharmaceutical Co., Ltd. (Henan, China; Batch no. 180428). Chrysophanol (>98%) was purchased from Xi'an Huilin Biotechnology Co., Ltd. (Xi'an, China; Batch No. HL‐201130). Sodium arsenite (NaAsO_2_, ≥99.0%) was purchased from Sigma–Aldrich (St. Louis, MO, USA). l‐Ornithine (>98%) was purchased from APExBIO Co., Ltd.(Houston, USA; Batch No. B8919). Carboxymethylcellulose sodium (CMC‐Na) was obtained from Sinopharm Chemical Reagent Co., Ltd.(Shanghai, China). C8‐D1A cells were obtained from Pricella Biotechnology Co., Ltd. (Wuhan, China). Viral vectors (adeno‐associated virus) were obtained from OBiO Technology Co., Ltd. (Shanghai, China), and RNA reagents (siRNA sequences with transfection reagents) were provided by Mingsheng Biotechnology Co., Ltd. (Shenyang, China). Total glutathione/oxidized glutathione test kit, total superoxide dismutase test kit, ATP content test kit, NAD^+^ /NADH test kit, and urea test kit were purchased from Elabscience Biotechnology Co., Ltd. (Wuhan, China). Total lactate dehydrogenase test kit, pyruvate test kit, lactate test kit, alanine aminotransferase (ALT) test kit, aspartate aminotransferase (AST) test kit, and blood ammonia assay kit were purchased from Nanjing Jiancheng Bioengineering Institute (Nanjing, China). Ornithine content assay kit and CPS1, ASS1, OTC, ARG1 enzyme assay kits were purchased from Enzyme‐linked Biotechnology Co., Ltd. (Shanghai, China). The details of the Kits are shown in Table S1 (Supporting Information).

### Animal Modeling and Disposal

SPF female ICR mice, aged 4–6 weeks and weighing 15–20 g, were provided by Huafukang Biotechnology Co., Ltd (Animal Licence No.: SCXK (Beijing) 2019‐0008), and were grouped according to the random number table method after 1 week of acclimatization feeding. After 1 week of acclimatization, the animals were grouped according to the random number table method. Female mice were selected because the population with conditions *NiuHuang JieDu* tables targets (constipation, oral ulcers) is predominantly female. Additionally, epidemiological surveys and animal experiments show that females are more sensitive to arsenic exposure.^[^ ^67^, ^68^, ^69^ ^]^ The gavage dose of realgar and chrysophanol was calculated by converting the equivalent dose between humans and mice based on the relevant provisions of *Niuhuang Jiedu Tablets* in the *Chinese Pharmacopoeia* (2020).^[^ ^61^ ^]^

Animal model ①: Thirty‐six ICR mice were divided into Control (Con), 0.45 g kg^−1^ realgar (Rea_L), and 1.35 g kg^−1^ realgar (Rea_H) groups. The Con group was intragastrically administered 0.5% (*w/v*) sodium carboxymethylcellulose (CMC‐Na). The Rea_L, Rea_H group was intragastrically administered with realgar suspended in 0.5% CMC‐Na at a dose of 0.45 g kg^−1^, 1.35 g kg^−1^, once a day, and the dosages were adjusted weekly according to the body weight changes. Neurobehavioral experiments were carried out on the tenth week, and the samples were taken at the end of the twelfth week for the subsequent experiments. This experiment was approved by the Institutional Animal Care and Use Committee (IACUC) of China Medical University (Shenyang, Liaoning, China)(Ethics Approval No. CMU2022002). The specific procedure is shown in Figure S1 (Supporting Information).

Animal model ②: Thirty‐two ICR mice were divided into four groups, the Con group was intragastrically administered 0.5% CMC‐Na suspension, the Rea_H group, the Rea_H+NC^GfABC1D^ KD group, and the Rea_H+*Zbtb7a* ^GfABC1D^ KD group were intragastrically administered 1.35 g kg^−1^ realgar suspension. Mice in the Rea_H+NC^GfABC1D^ KD group and Rea_H+*Zbtb7a* ^GfABC1D^ KD group were given a single injection of Adeno‐Associated Virus (AAV) at a titre of ≥1.0 × 10^12^ vg mL^−1^ via stereotaxic brain injection at the beginning of week 7, and the injection site was the bilateral frontal lobe (AP: +1.98 mm; ML: ±0.4 mm; DV: −1.8 mm), the viral vector pAAV2/5‐GfABC1D‐EGFP‐3xFLAG‐miR30shRNA(NC)‐WPRE was used as a control, pAAV2/5‐GfABC1D‐EGFP‐3xFLAG‐miR30shRNA(ZBTB7A)‐WPRE was used for knockdown of the ZBTB7A expression in frontal astrocytes. (Ethical approval number: CMU20240181). The specific process is shown in Figure S2 (Supporting Information). The shRNA sequences are shown in Table S2 (Supporting Information).

Animal model ③: Thirty‐two ICR mice were divided into four groups, the Con group was intragastrically administered 0.5% CMC‐Na suspension, the Rea_H group, the Rea_H+NC^TBG^ OE group, and the Rea_H+*Otc* ^TBG^ OE group were intragastrically administered 1.35 g kg^−1^ realgar suspension. Mice in the Rea_H+NC^TBG^ OE group and Rea_H+*Otc* ^TBG^ OE group were given a single injection of AAV at a titer of ≥2.5 × 10^12^ vg mL^−1^ by tail vein injection at the beginning of week 7, and the viral vectors pAAV2/8‐TBG‐GdGreen‐WPRE were used as a control; pAAV2/8‐TBG‐Otc‐3×FLAG‐P2A‐GdGreen‐WPRE was used to enable OTC overexpression in hepatocytes. (Ethical approval number: CMU20240181). The specific process is shown in Figure S3 (Supporting Information).

Animal model ④: Fifty ICR mice were divided into five groups, The Con group (Con) was intragastrically administered 0.5% CMC‐Na suspension, the 1.35 g kg^−1^ realgar group (Rea_H) was intragastrically administered 1.35 g kg^−1^ realgar suspension, the 0.04 g kg^−1^ chrysophanol group (Chr) was intragastrically administered 0.04 g kg^−1^ chrysophanol suspension, the 1.35 g kg^−1^ realgar plus 0.04 g kg^−1^ chrysophanol group (Rea_H+Chr_L) was intragastrically administered 1.35 g kg^−1^ realgar plus 0.04 g kg^−1^ chrysophanol suspension, and the 1.35 g kg^−1^ realgar plus 0.08 g kg^−1^ chrysophanol group (Rea_H+Chr_H) was intragastrically administered 1.35 g kg^−1^ realgar plus 0.08 g kg^−1^ chrysophanol suspension once a day for 12 weeks. (Ethical approval number: CMU2022002). The specific procedure is shown in Figure S4 (Supporting Information).

### Cell Culture and Disposal

The C8‐D1A murine astrocyte cell line (Pricella, Wuhan) was cultured in DMEM supplemented with 10% fetal bovine serum and 1% penicillin‐streptomycin (not ampicillin). Cells were maintained at 37°C in a 5% CO_2_ humidified atmosphere (Thermo Fisher Scientific, MA, USA) with >95% relative humidity. All experiments used cells within 20 passages.

Arsenite exposure: Sodium arsenite (NaAsO_2_) was dissolved in phosphate‐buffered saline (PBS) to prepare 10 mm iAs^3^⁺ stock solution. Working concentrations were freshly diluted in complete medium before treatment.

Ornithine treatment: l‐Ornithine was dissolved in sterile dimethyl sulfoxide (DMSO) to generate 100 mm stock solution, with a final DMSO concentration ≤0.1% in all experiments.

siRNA transfection: RNATransMate and siRNA were added to centrifuge tubes A and B containing serum‐free medium to dilute the cells once the confluence reached 60–70%. The tubes were then mixed and allowed to stand at room temperature for 10 min to form an RNA/RNATransMate complex. After that, RNA/RNATransMate was added to the culture dish, and the cells were collected 24–48 h after transfection. The siRNA sequences are shown in Table S3 (Supporting Information).

### AAV Procedure for Experimental Animal Brain Stereotactic Injection

The Lamda and Bregma locations on the mice's cranial surface were completely exposed, the skin was prepped, and the animals were secured with ear rods. According to the atlas of The Mouse Brain in Stereotaxic Coordinates,^[^ ^70^ ^]^ the Bregma point's coordinates were set as the origin, and the prefrontal lobe's injection point's coordinates were set as follows: AP: +1.98 mm; ML: ± 0.4 mm; DV: −1.8 mm. A tiny hole was then carefully drilled. AAV was aspirated using an ultra‐micro syringe pump (RWD, Shenzhen, China). The needle was then lowered by 1.8 mm to the injection site, and the injection was carried out at a rate of 0.25 µL min^−1^.

### The Process of Injecting AAV into Experimental Animals' Tail Veins

Mice were placed in a holder with their tails completely exposed. To promote vascular dilatation and filling, the tails were gently squeezed at the root and regularly cleaned with an alcohol cotton ball. An insulin needle was used to aspirate 200 µL of AAV, which was then gradually injected into the mouse's back 1/4 of the tail, in a manner parallel to the vein.

### Morris Water Maze

A 15 cm diameter escape platform was submerged 1 cm below the water surface in the center of the target quadrant within a 1.5 m diameter circular pool. The pool featured black interior surfaces to minimize visual cues. During acquisition trials, mice were introduced facing the pool wall at randomized entry points. Trials terminated automatically upon platform location or after 60 s maximum duration. Subjects underwent four daily trials from different cardinal starting positions across five consecutive training days. Following a 7‐day retention interval, probe testing was conducted with the platform removed. Time spent in the target quadrant during the 60‐s probe trial was recorded.

### Novel Object Recognition

The mice spent 3 min exploring freely in an empty 60 cm × 60 cm × 60 cm box on the first day. The mice were taught for three days in a row on the second and fourth days. During this time, two identically colored cylinders, A and B, were positioned diagonally 10 cm from the box's wall, and the mice explored for 3 min.

The mice were allowed to wander for 5 min on the sixth day of the formal experiment by swapping out one of the cylinders (A) for the square (C). The amount of time spent examining both new and familiar items was noted. It was noted how long it took the mice to investigate the new and familiar items.

### Open Field Test

A camera, a recording device, and a 60 cm × 60 cm × 60 cm black empty box make up the open field system. The bottom of the open field was separated into nine equal sections based on the experimental observation indices, with the central region measuring 20 cm by 20 cm. Every mouse was put in the box in the same spot and allowed to roam around for 5 min. During that time, the average speed, total distance traveled, cumulative number of times, and amount of time spent in the center were noted.

### Elevated Plus Maze

The elevated plus maze is typically set to a height of 50 cm above the ground and is made up of two open and two closed arms that form a cross shape. After being taken out, the mice were carefully positioned in the middle of the maze and allowed to roam around for 5 min. The amount of time spent in the open and closed arms was noted.

### Determination of Total Arsenic

In accordance with our team's earlier investigation,^[^ ^8^ ^]^ a Shimadzu inductively coupled plasma mass spectrometer Inductively coupled plasma mass spectrometry‐2030 (ICP‐MS‐2030) (Shimadzu, Japan) instrument was employed. 50 mg of mouse liver or brain tissue were weighed, combined with 500 µL of deionized water, and properly mixed. Next, 1500 µL of 15 m HNO_3_ was added, and the mixture was allowed to digest for 48 h at room temperature. Following digestion, the supernatant was centrifuged for 5 min at 12 000 rpm and then added to the ICP‐MS to measure the amount of arsenic.

### Ultrastructure of Neurons, Astrocytes, and Hepatocytes

Following animal execution, approximately 1 mm^3^ of tissue was quickly cut at a low temperature using 2.5% glutaraldehyde phosphate buffer fixed at 4 °C for 24 h, osmium fixation for 2 h, gradient alcohol dehydration (50% alcohol 30 min, 70% alcohol 30 min, and 100% acetone 10 min, three times), polymerization, block repair, ultrathin microtome sections, uranyl acetate and lead citrate staining, and transmission electron microscopy (HITACH, Japan) to view the ultrastructure.

### Single‐Cell/Single‐Cell Nuclear Transcriptome Sequencing

Following the manufacturer's (China SeekOne) protocol, tissues were separated to create a single‐cell or single‐nucleus suspension. After staining with AO/PI cell staining solution, the cells were counted and assessed for quality control (QC). To construct a single‐cell transcriptome library that is compatible with high‐throughput sequencers, the cell suspension, along with reverse transcription reagents, was placed into the SeekOne®DD chip. The filtered cell‐gene expression matrix was utilized for further analysis after quality control was completed on the raw sequencing data. Data dimensionality reduction and visualization were performed using nonlinear techniques, specifically t‐SNE or UMAP. Cell type annotation was subsequently conducted by either referencing the SingleR dataset or identifying established target cell type markers within the CellMarker 2.0 database (http://117.50.127.228/CellMarker/).

### Single‐Cell/Single‐Cell Nuclear Transcriptome Sequencing—Bioinformatics Analysis

Differential gene expression analysis was performed on specific cell subpopulations between experimental and control groups using the FindMarkers function within the Seurat package. This analysis identified genes significantly upregulated or downregulated in the experimental group compared to the control. Lianchuan BioCloud (www.omicstudio.cn/) was used to map the volcanoes of differently expressed genes (DEGs). KEGG enrichment analysis of genes with differential expression was carried out using the David online bioinformatics resource (https://davidbioinformatics.nih.gov/).

### LC‐MS Metabolomics Sequencing

Ice‐cold methanol:acetonitrile (v/v, 1:1), 400 µL, was mixed with 100 µL of mouse plasma and vortexed vigorously. Sonication was done in an ice bath for an hour. After that, the supernatant was gathered and examined using a Q‐Exactive Plus system in conjunction with UPLC‐ESI‐Q‐Orbitrap‐MS.

### LC‐MS Metabolomics Sequencing—Bioinformatics Analysis

Multidimensional statistical analyses were performed using SIMCA‐P 14.1 (Umetrics, Umeå, Sweden), including pattern recognition and orthogonal partial least squares‐discriminant analysis (OPLS‐DA). Hierarchical cluster analysis and correlation matrices were generated using R (version 4.3.1). Receiver operating characteristic (ROC) curves were constructed using the Lianchuan BioCloud platform (http://www.omicstudio.cn/). The KEGG (https://www.genome.jp/kegg/pathway.html) and SMPDB (https://smpdb.ca/) databases were used to conduct metabolite pathway enrichment analysis.

### Western Blot

RIPA lysate (Biyuntian, Shanghai, China) was used to lyse tissues or cells, and a BCA kit (Takara Bio Inc., Shiga, Japan) was used to measure the total protein concentration. After being separated by SDS‐PAGE gel electrophoresis at 6–12%, the measured proteins were moved to a PVDF membrane (Millipore, Germany), sealed, and incubated with the particular primary antibody for the entire night at 4 °C. Following incubation, the membranes were conjugated for 1 h with the appropriate anti‐mouse or anti‐rabbit IgG antibody. The Tanon 5200 (Tanon, Shanghai, China) with ECL emitting solution was used to expose and develop the membranes. The antibody dilutions are shown in Table S4 (Supporting Information).

### Real‐Time PCR

Trizol was used to extract the RNA, and the PrimeScript RT kit's instructions (RR047A, Takara, Japan) were followed to reverse‐transcribe the total RNA into cDNA. The QuantStudio 6 Flex Real‐Time PCR System (Life Technologies) was then used to quantify the samples after they had been processed using the SYBR® Premix Ex Taq II kit (RR820A, Takara, Japan). Following treatment of the samples with the SYBR® Premix Ex Taq II kit (RR820A, Takara, Japan), the target genes were then quantified using the QuantStudio 6 Flex Real‐Time PCR System (Life Technologies). Using β‐actin as an internal reference, the 2^−ΔΔCt^ technique was used to rectify and analyze the data. The primer sequences are shown in Table S5 (Supporting Information).

### Immunofluorescence

Tissue samples fixed in 4% paraformaldehyde were paraffin‐embedded for a full day. Sections underwent antigen repair after being deparaffinized in xylene for 2 min apiece. Following xylene deparaffinization, the sections were dehydrated for 2 min each in graded alcohol (100%, 95%, 85%, 75%, and 50%), mended antigenically, and sealed before being incubated with primary antibody for the entire night at 4 °C. After washing the following day, fluorescent secondary antibody was added, and the mixture was incubated for 1 h at room temperature. Following washing, the slices were sealed using an anti‐fluorescence quenching sealer that contained DAPI (Solarbio, Beijing, China). They were then examined and captured on camera using a fluorescence microscope. The antibody dilutions are shown in Table S6 (Supporting Information).

### Oxidative Stress Levels

Mouse frontal lobe tissues were weighed and homogenized (10% w/v) in ice‐cold physiological saline using a tissue‐to‐solvent ratio of 1:9 (mg:µL). The homogenates was centrifuged for 10 min at 4000 rpm and 4 °C, and the supernatant was removed for measurement. As per the kit's instructions, a colorimetric approach was used to measure the amount of superoxide dismutase (T‐SOD), total glutathione (T‐GSH), and oxidized glutathione (GSSG) in the mice's frontal lobe.

### Energy Metabolism Levels

As directed by the Biochemistry Kit, either gather cells and cell culture media or make a 10% tissue homogenate. Using a colorimetric technique, ascertain the concentration of the following energy metabolism‐related indices: pyruvate, lactate, total lactate dehydrogenase, ATP, and NAD^+^/NADH.

### Metabolite Levels of Ornithine Cycle

As directed by the kit, prepare the necessary reagents and draw the standard curves. Make a 10% tissue homogenate, then measure the supernatant. Direct measurements are made of plasma. As directed by the kit, ascertain the amount of ornithine, ammonia, and urea in mouse plasma as well as the amount of ornithine in liver and brain tissues.

### **HE** Staining

Liver tissues soaked in paraformaldehyde for 24 h were sectioned and paraffin‐embedded. The sections were dewaxed for 5 min each in xylene I and II, and then hydrated for 5 min each in gradient alcohol and distilled water. After 5 min of staining with hematoxylin staining solution, the sections were differentiated for 1 to 3 s using 1% hydrochloric acid. Following 10–15 s of staining with a 0.5% eosin staining solution, the sections were successively dehydrated by immersing them in gradient alcohol for 5 min each, followed by 5 min each of xylene I and II. Finally, one drop of neutral tree resin was added to the sections, and they were sealed. Under a microscope, the samples were examined and captured on camera.

### ALT and AST Viability Assays

A 10% (w/v) liver tissue homogenate was prepared in ice‐cold PBS (0.1 m, pH 7.4). The assay and control wells were set, the OD value of each well was determined using the enzyme marker, the standard curve was created in accordance with the kit operating table, and the corresponding AST/GPT and ALT/GOT activities were obtained by checking the standard curve using the absolute OD value.

### Enzyme Tests for Ornithine Cycle Rate‐Limiting (CPS1, ASS1, OTC, ARG)

In accordance with the ELISA kit, a 10% liver tissue homogenate and washing buffer were made. ELISA was used to measure each well's absorbance at 450 nm. The standard's linear regression curve was plotted, the concentration value of each sample was determined using the curve equation, and the standard's concentration was plotted as the horizontal coordinate, the corresponding OD value as the vertical coordinate, and the blank well as the 0 well.

### Prediction of Transcription Factor and Gene Promoter Binding Sites

The promoter area is the default region. The transcription start site was obtained from the NCBI website's “gene” database (https://www.ncbi.nlm.nih.gov/gene/term) and ranged from +100 to −1000 bp. The JASPAR database (http://jaspar.genereg.net/) provided the transcription factor ZBTB7A (MA0750.2) binding motif (‐CCGGAAGTG‐). The binding site of ZBTB7A to the gene's promoter region was predicted, and its location and score were examined.

### Molecular Docking

The 3D structure of ZBTB7A (PDB ID: 8H9H) was retrieved from the Protein Data Bank (PDB) (http://www.rcsb.org/). The 3D conformation of ornithine (MOL000054) was acquired from the Traditional Chinese Medicine Systems Pharmacology (TCMSP) database (https://www.tcmsp‐e.com/#/database). Molecular docking simulations were performed using AutoDock 4.2.6 with Lamarckian genetic algorithm parameters, and docking complex visualization and interaction analysis were conducted in PyMOL‐X 2.1.1 (Schrödinger, LLC).

### ChIP‐qPCR

Following the procedures for chromatin immunoprecipitation (Guangzhou OneMed Biomedical Technology Co., Ltd., Guangzhou, China), 1 × 10^7^ C8‐D1A cells were gathered, subjected to glycine neutralization and formaldehyde cross‐linking, had their nuclei extracted, had their chromatin cleaved by ultrasonication, undergone immuno‐enrichment, and had their DNA recovered using an adsorption column before being used in the qPCR tests. The sequences of the promoter primers are shown in Table S7 (Supporting Information).

### CCK8 Assay

Fill the 96‐well plate with 100 µL of C8‐D1A cell suspension. After the cells have reached 50% density, add 10 µL of various iAs^3+^ and Orn concentrations. The plate should then be incubated for 24 h. Fill each well with 10 µL of CCK‐8 solution (Shanghai YEASEN Biological Science and Technology Co., Ltd.), and then place the plate in the incubator for 1–4 h. An enzyme marker was used to quantify the absorbance at 450 nm. Cell viability (%) = [A (plus drug) – A (blank)]/[A (0 plus drug) – A (blank)]× 100.

### Cellular Thermal Shift Assay

C8‐D1A cells were harvested and lysed according to the method established by Guan et al.^[^ ^71^ ^]^ Following lysis, homogenates were centrifuged at 20 000 ×g for 20 min at 4 °C, transfer the supernatant, and then add various medication concentrations to thoroughly mix and incubate for 30 min at room temperature. React for 3 min at various temperature gradients, then cool to ambient temperature before being centrifuged at 20 000 ×g and high‐speed centrifugation for 20 min at 4 °C. Finally, add the sample buffer for target protein Western blot detection.

### Statistical Analysis

All data were expressed as mean ± standard deviation (x±SD), and statistical analyses and graphs were produced using GraphPad Prism 8.0 software (GraphPad software, San Diego, CA, USA). Multiple comparisons between groups were performed by one‐way analysis of variance (ANOVA), *p *< 0.05 was considered to indicate a significant difference.

## Conflict of Interest

The authors declare no conflict of interest.

## Supporting information



Supporting Information

## Data Availability

The data that support the findings of this study are available from the corresponding author upon reasonable request.
