# Supplementary material for: Realgar‐Induced CNS Toxicity: Exploring OTC‐Mediated Ornithine Regulation of ZBTB7A Inhibits Astrocyte Glycolysis Based on the Liver–Brain Axis
Source: Adv Sci (Weinh). 2025 Nov 21;13(2):e02591. doi: 10.1002/advs.202502591 (PMC12786332; doi:10.1002/advs.202502591)
Supplement: Supplementary file 1 — Supporting Information [file ADVS-13-e02591-s001.docx]

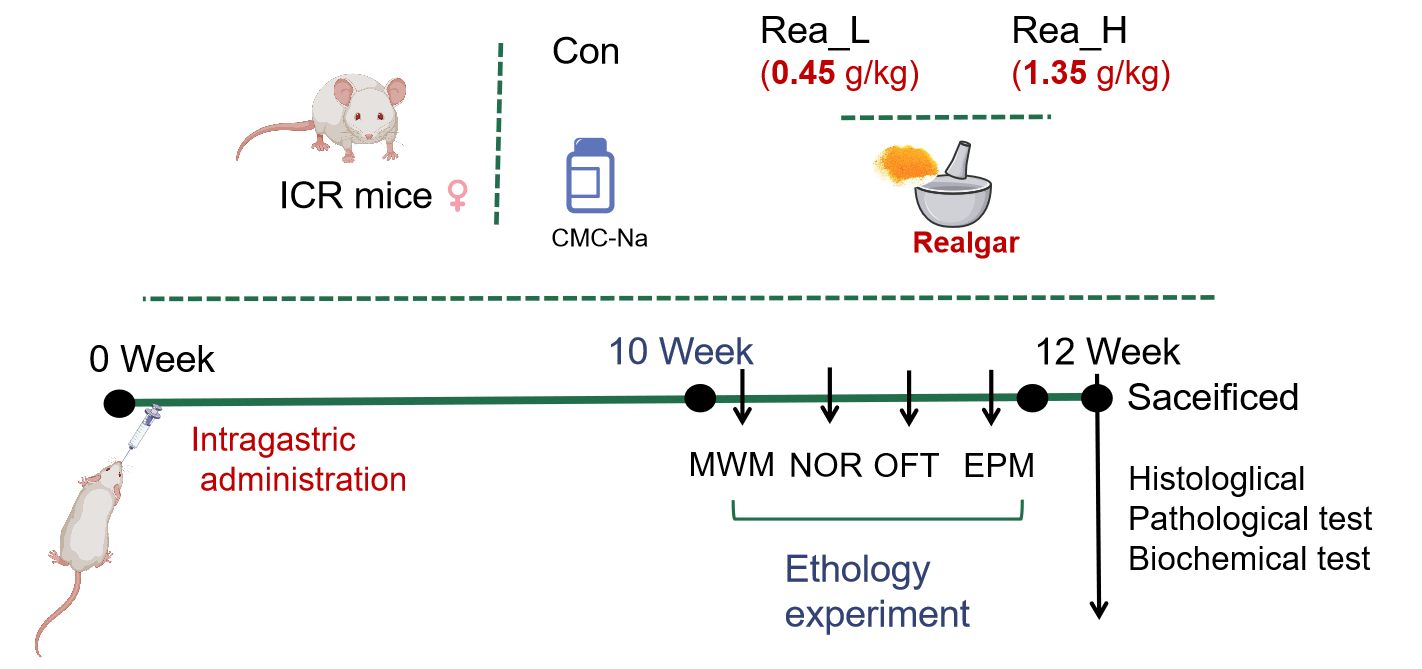

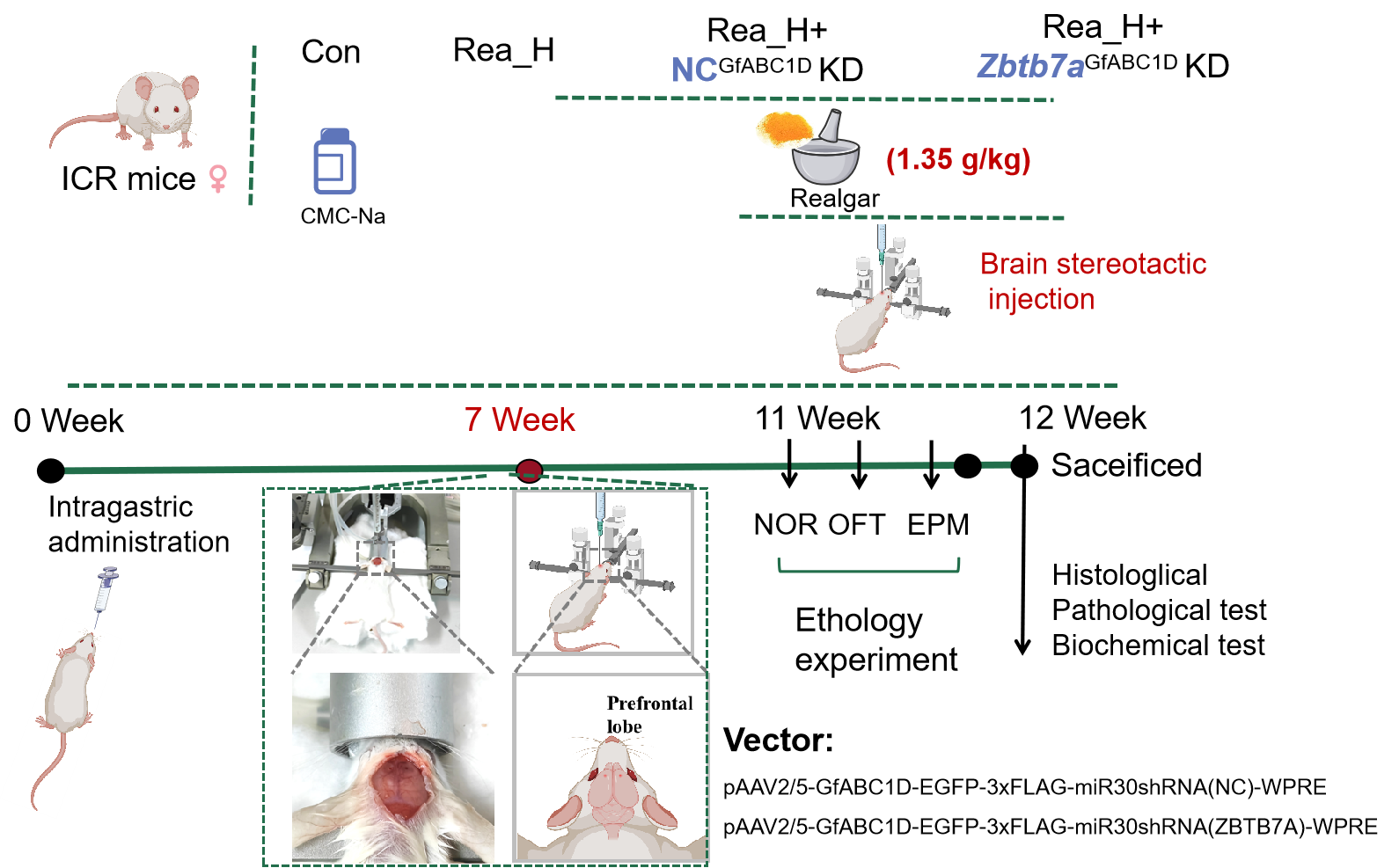


**Supplementary Figures and tables**

**Figure S1. Flow chart of the grouping and processing of realgar-exposure experimental animals**

**Figure S2. Flow chart of the grouping and processing of *Zbtb7a*^GfABC1D^ KD experimental animals**


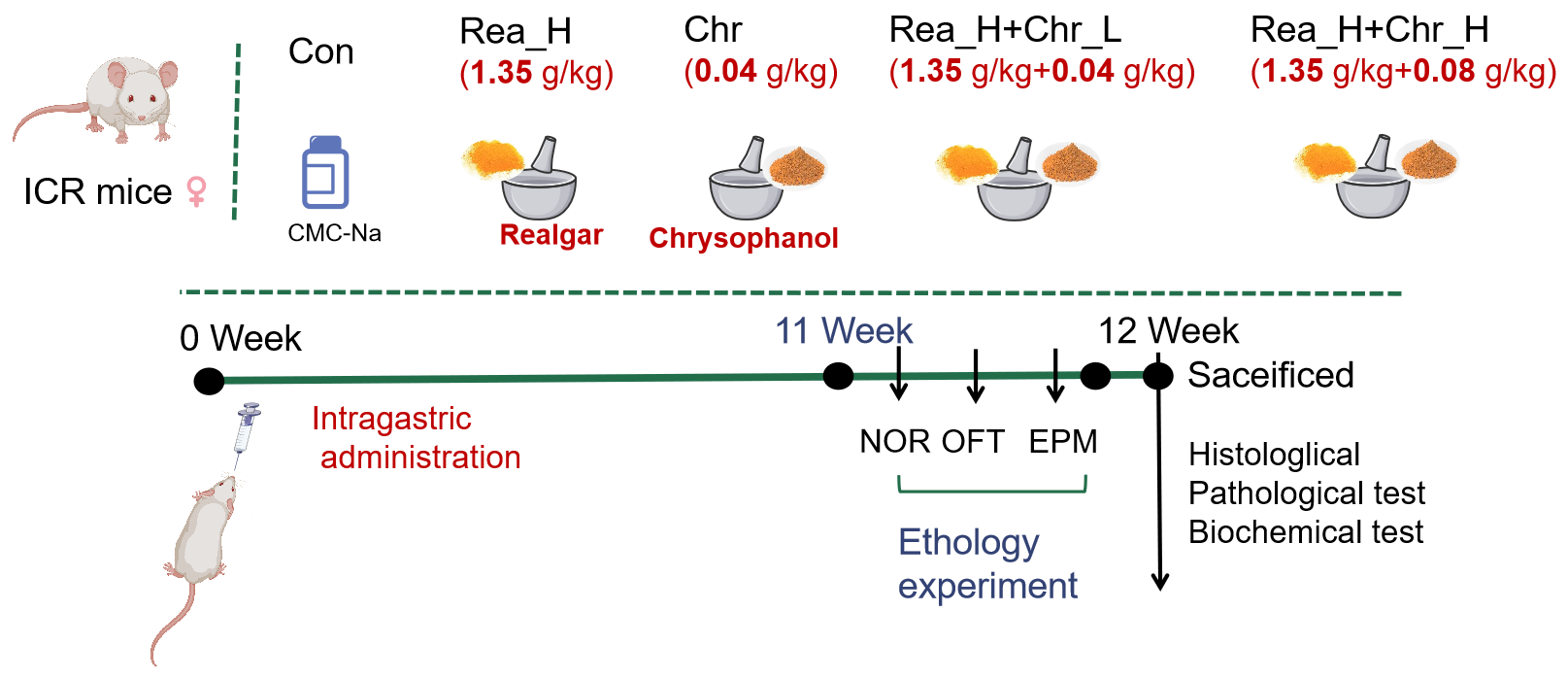

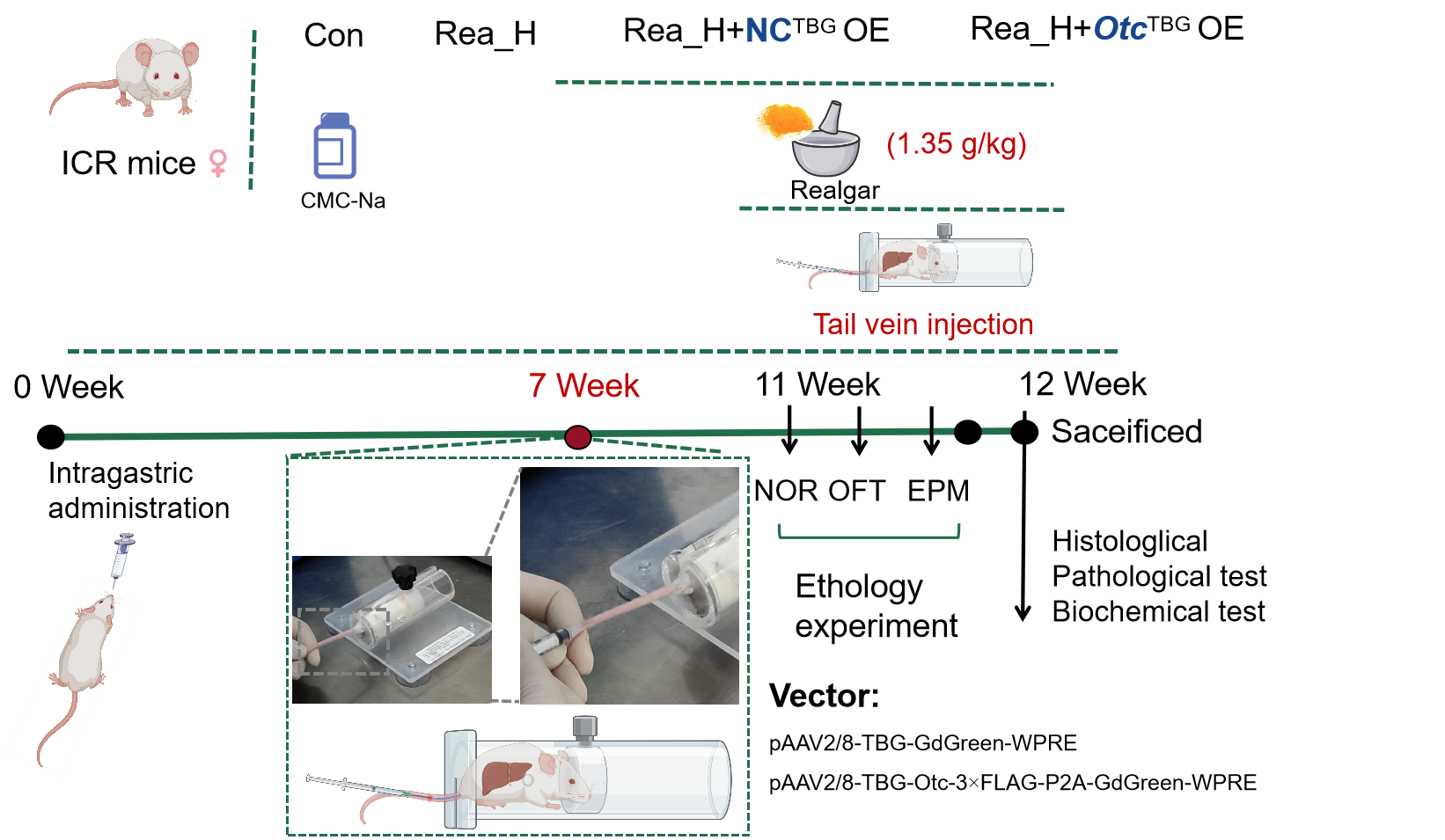


**Figure S3. Flow chart of the grouping and processing of *Otc*^TBG^ OE experimental animals**

**Figure S4. Flow chart of the grouping and processing of Chrysophanol intervention**

**experimental animals**

**Table S1. Details of the Kits**

| Kits | Catalog | Company |
| --- | --- | --- |
| T-GSH/GSSG test kit | E-BC-K097-M | Elabscience Biotechnology Co., Ltd. (Wuhan, China) |
| T-SOD test kit | E-BC-K020-M |  |
| ATP content test kit | E-BC-K157-M |  |
| NAD^+^ /NADH test kit | E-BC-K804-M |  |
| Urea test kit | E-BC-K183-M |  |
| Total lactate dehydrogenase test kit | A020-1 | Nanjing Jiancheng Bioengineering Institute (Nanjing, China) |
| Pyruvate test kit | A081-1-1 |  |
| Lactate test kit | A019-2-1 |  |
| ALT test kit | C009-2-1 |  |
| AST test kit | C010-2-1 |  |
| Blood ammonia assay kit | A086-2-1 |  |
| Ornithine content assay kit | ml941620V | Enzyme-linked Biotechnology Co., Ltd. (Shanghai, China) |
| CPS1 ELISA kits | ml986655V |  |
| ASS1 ELISA kits | ml058171V |  |
| OTC ELISA kits | ml369540V |  |
| ARG1 ELISA kits | ml106177V |  |


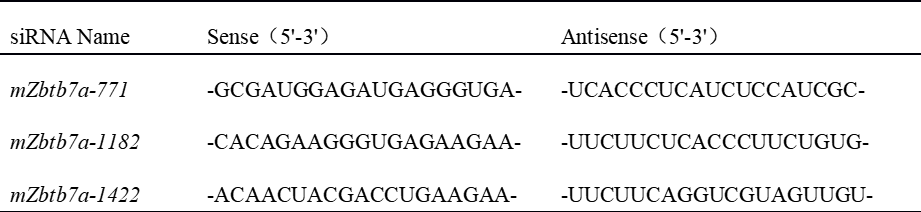


**Table S2. Target-shRNA sequence for AAV**

| Vector name | Sense（5'-3'） |
| --- | --- |
| pAAV2/5-GfABC1D-EGFP-3xFLAG-miR30shRNA(NC)-WPRE | -GCGATGGAGATGAGGGTGA- |
| pAAV2/5-GfABC1D-EGFP-3xFLAG-miR30shRNA(ZBTB7A)-WPRE | -CTCGCTTGGGCGAGAGTAA- |

**Table S3. Sequence for siRNA**

| siRNA Name | Sense（5'-3'） | Antisense（5'-3'） |
| --- | --- | --- |
| *mZbtb7a-771* | -GCGAUGGAGAUGAGGGUGA- | -UCACCCUCAUCUCCAUCGC- |
| *mZbtb7a-1182* | -CACAGAAGGGUGAGAAGAA- | -UUCUUCUCACCCUUCUGUG- |
| *mZbtb7a-1422* | -ACAACUACGACCUGAAGAA- | -UUCUUCAGGUCGUAGUUGU- |

**Table S4. Antibody for Western blot**

| Antibodies | Product code | Dilution rate | Supplier |
| --- | --- | --- | --- |
| Rb-anti-BCL2 Polyclone | 26593-1-AP | 1:1000 | Proteintech |
| Rb-anti-BAX Polyclone | 50599-2-Ig | 1:1000 | Proteintech |
| Mc-anti-GFAP Monoclonal | 60190-1-Ig | 1:8000 | Proteintech |
| Rb-anti-C3 Polyclone | 213337-1-AP | 1:1500 | Proteintech |
| Rb-anti-CCL3 Polyclone | YT8075 | 1:1000 | Immunoway |
| Rb-anti-CCR5 Polyclone | YT6108 | 1:1000 | Immunoway |
| Rb-anti-ALDOA Polyclone | YT0191 | 1:1000 | Immunoway |
| Rb-anti-LDHA Polyclone | YN3033 | 1:1000 | Immunoway |
| Rb-anti-PGAM1 Polyclone | YN5398 | 1:1000 | Immunoway |
| Rb-anti-ZBTB7A Polyclone | Bs-0819R | 1:1000 | Bioss |
| Rb-anti-NCoR1 Polyclone | YT2999 | 1:1000 | Immunoway |
| Rb-anti-HDAC4 Polyclone | TA6349S | 1:1000 | Abmart |
| Rb-anti-CAT-1 Polyclone | YN3729 | 1:1000 | Immunoway |
| Rb-anti-CPS1 Polyclone | YN4846 | 1:1000 | Immunoway |
| Rb-anti-ARG1 Polyclone | YT0311 | 1:1000 | Immunoway |
| Rb-anti-OTC Polyclone | YN3102 | 1:1000 | Immunoway |
| Rb-anti-ASS1 Polyclone | YN5812 | 1:1000 | Immunoway |
| Rb-anti-β-Actin Polyclone | 20536-1-Ig | 1:8000 | Proteintech |
| Rb-anti-Tubulin β Polyclone | AF7011 | 1:5000 | Affinity |
| Goat Anti Mouse IgG(H+L) (HRP) | RS0001 | 1:10000 | Immunoway |
| Goat Anti Rabbit IgG(H+L) (HRP) | RS0002 | 1:10000 | Immunoway |

**Table S5. Primer sequence for RT-qPCR**

| Genes | Forward（5'-3'） | Reverse（5'-3'） |
| --- | --- | --- |
| *Ldha* | -CGTCCCGAACAACAAGA- | -CTTCCAACACATCCACCA- |
| *Aldoa* | -GCTGAATAGGCTGCGTTCT- | -GGTGGGTAGGCGAAAGG- |
| *Pgam1* | -CAGCAACATCAGCAAGGAT- | -TCATTCCAGAAGGGCAGT- |
| *Zbtb7a* | -GTCTGTCCCCTCCCCAT- | -CCTGTGGCTCGGTCAATAC- |
| *Cps1* | -AGAGATGGACGCTGTTGG- | -CCTTGGCTGATGGTCTGT- |
| *Ass1* | -GGAAAGCAGACTACACGGA- | -CTTCAGCCACACGAGGA- |
| *Otc* | -GGATCAAGCAGAAAGGAGAA- | -GTGGTAAGAAAGGAAGGGTGT- |
| *Arg1* | -ACGGTCTGTGGGGAAAG- | -TCAGGGGAGTGTTGATGTC- |
| *Slc7a1* | -CCCTTCTCCTCTCACTTGG- | -TCGCTGGGATAGTCTTGC- |
| *β-actin* | -TCTTTGCAGCTCCTTCGT- | -GACCCATTCCCACCATC- |

**Table S6. Antibody for immunofluorescence**

| Antibodies | Product code | Dilution rate | Supplier |
| --- | --- | --- | --- |
| Mc-anti-GFAP Monoclonal | 60190-1-Ig | 1:200 | Proteintech |
| Rb-anti-C3 Polyclone | 213337-1-AP | 1:200 | Proteintech |
| Rb-anti-ZBTB7A Polyclone | Bs-0819R | 1:1000 | Bioss |
| FITC-conjugated Goat anti-Mouse IgG (H+L) | AS001 | 1:200 | Abclonal |
| Cy3-conjugated Goat anti-Rabbit IgG (H+L) | AS007 | 1:400 | Abclonal |

**Table S7. Primer sequence for ChIP-qPCR**

| Genes | Forward（5'-3'） | Reverse（5'-3'） |
| --- | --- | --- |
| *Ldha promoter*  (Specific site) | -ATCTCCTTCAAGGACAAGGCTG- | -AGACATAGGTTTCTATTAGCACAAGA- |
| *Ldha promoter*  (Non-Specific) | -TCTTTAAGTGTGTACCTCTT- | -TTTGAATCCCAGCAGTGAAAA- |
| *Aldoa promoter*  (Specific site) | -TCTCTGAACGTTTTGCCCGA- | -AGTGACTCACAGCCATCACG- |
| *Aldoa promoter*  (Non-Specific) | -GGTGTAAAGTAGCAACAAAAA- | -AAACTCACAGAGATCTATCTC- |
| *Pgam1 promoter*  (Specific site) | -ACTTTTCTTGGCTCTCTGTCG- | -CCTGTCCTTTTAGTGGAGAGCA- |
| *Pgam1 promoter*  (Non-Specific) | -GAAGTTTAATGGAAATAAAC- | -CAACATTTAGTAGAAGAAAAGGGAT- |


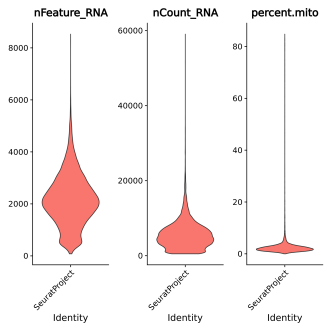

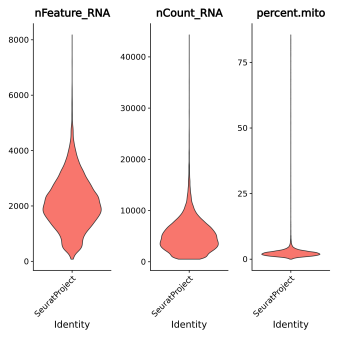


Con

Rea_H

Con

Rea_H


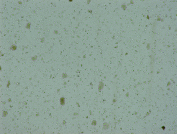

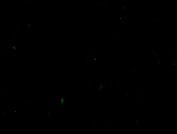

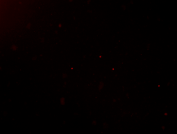

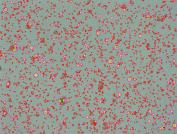

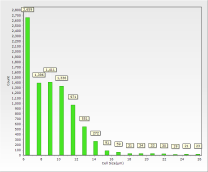

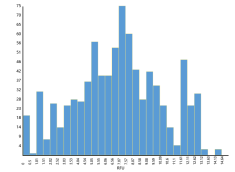

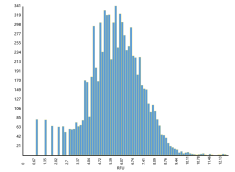

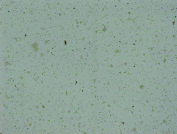

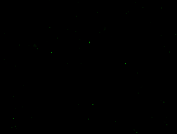

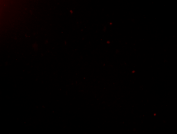

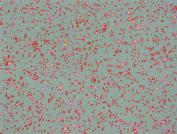

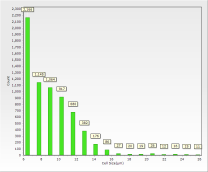

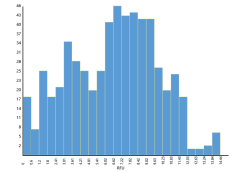

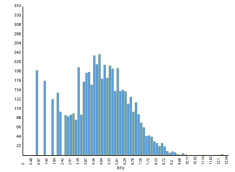


A

B

**Figure S5. Mouse frontal lobe single cell transcriptome sequencing QC plot.** (**A**) Con, Rea-H group single cell suspension final AOPI staining count quality control chart. (**B**) Con, Rea_H group cell gene number, total number of mRNA molecules, mitochondrial gene number violin chart.

**Table S8. Quality control data for single-cell libraries**

| Sample_name | Estimated_Number  _of_Cells | Mean_Reads  _per_Cell | Mean_Genes  _per_Cell | Valid_  Barcodes | Sequencing_  Saturation |
| --- | --- | --- | --- | --- | --- |
| Con | 13,666 | 30,772 | 2,036 | 87.22% | 60.07% |
| Rea_H | 14,845 | 33,904 | 2,106 | 86.93% | 61.91% |

**Table S9. Raw data filtering indicators and filtered data**

| Sample_name | Cell_num | UMI_threshold | UMI_filter | Mito_threshold | Mito_filter |
| --- | --- | --- | --- | --- | --- |
| Con | 13,666 | 300 | 13,666 | 10 | 13,498 |
| Rea_H | 14,845 | 300 | 14,845 | 10 | 14,544 |


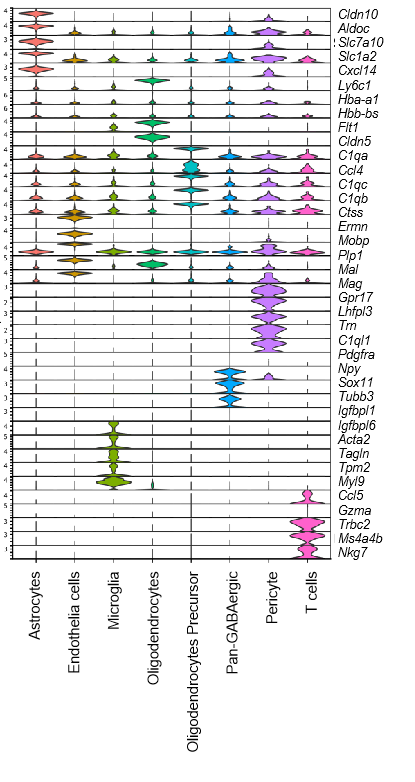


B


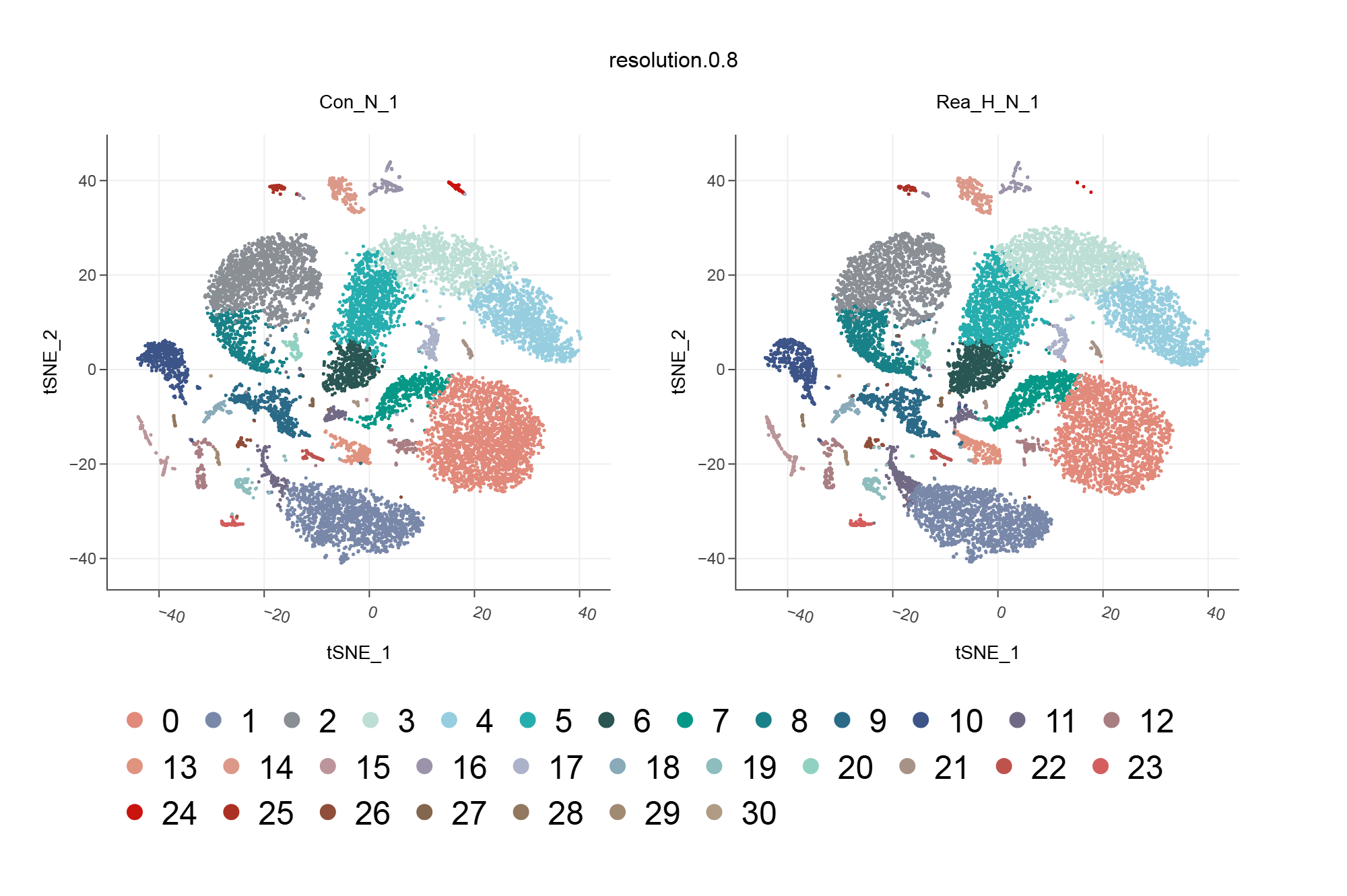

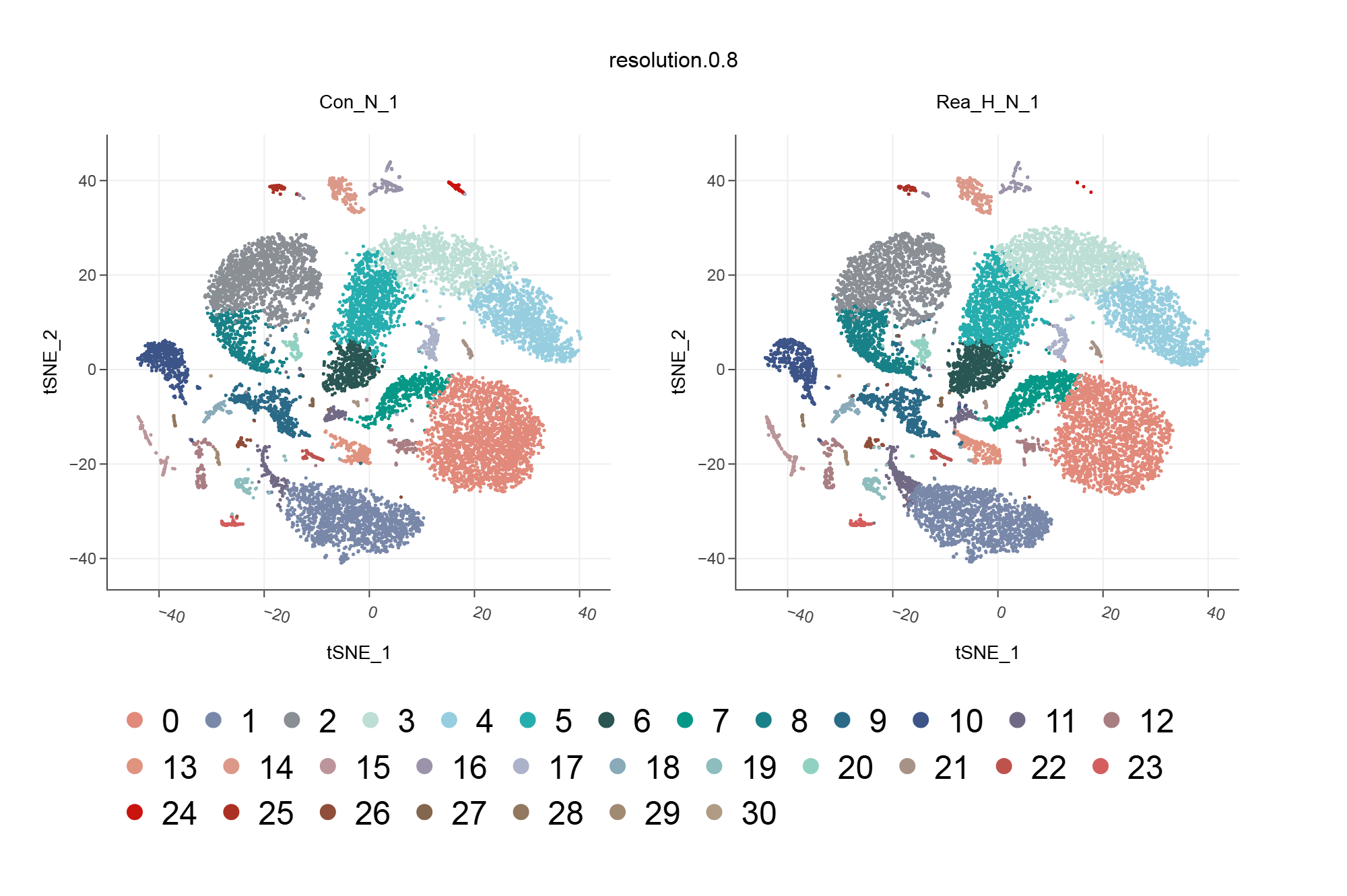


Con

Rea_H

A

C


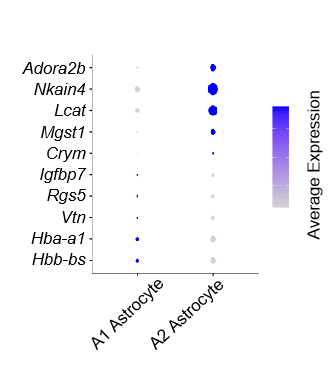

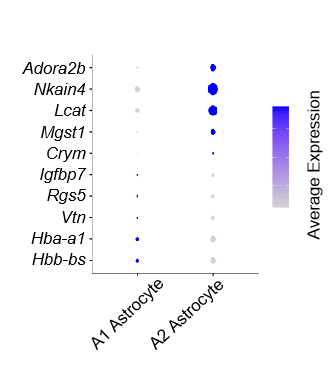

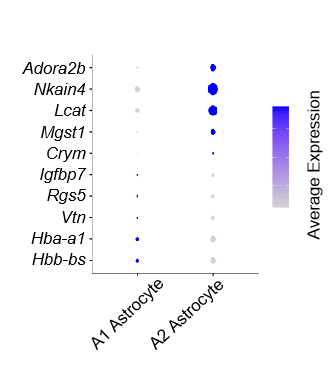


E

Con Rea_H


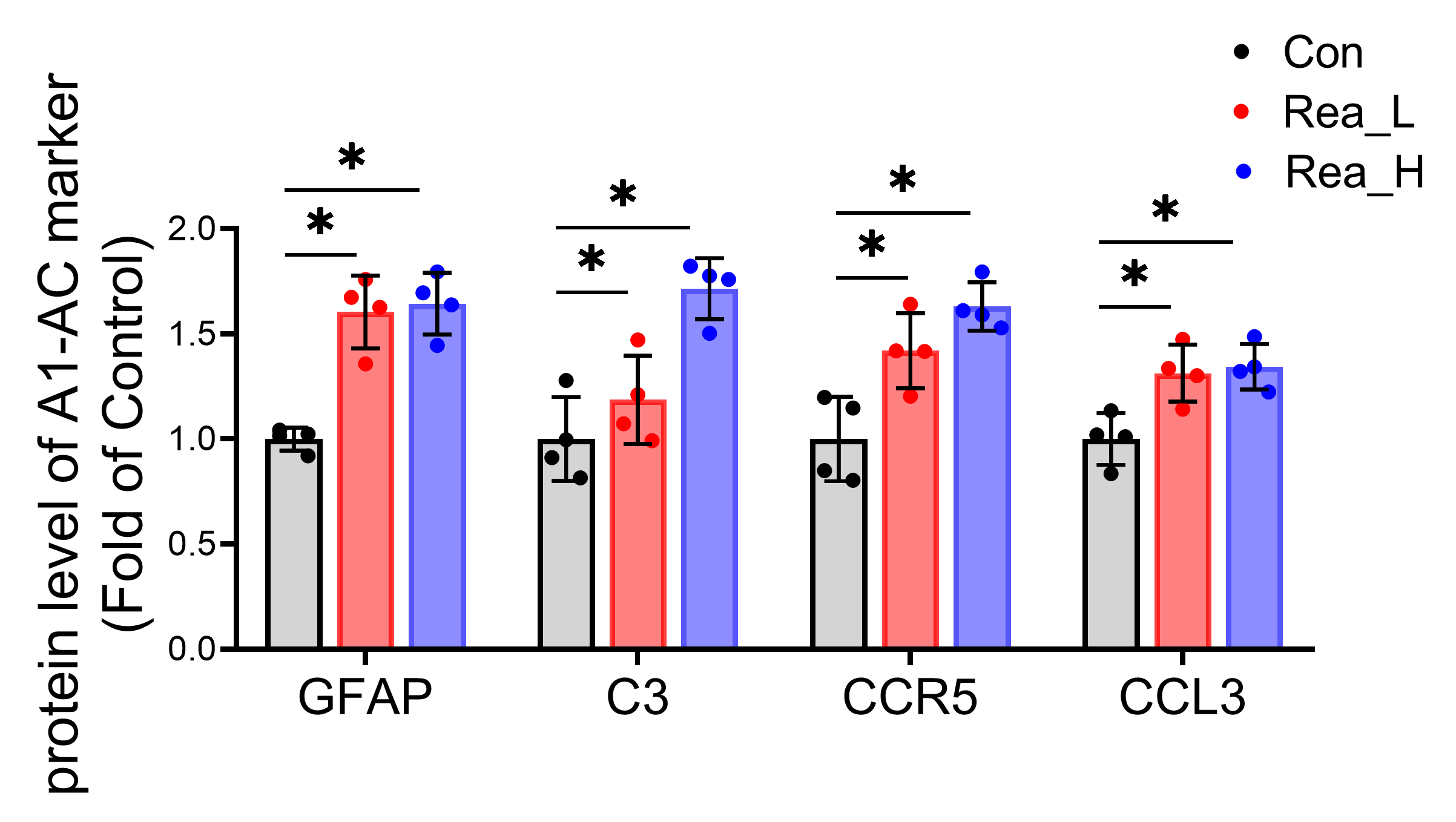


D

F


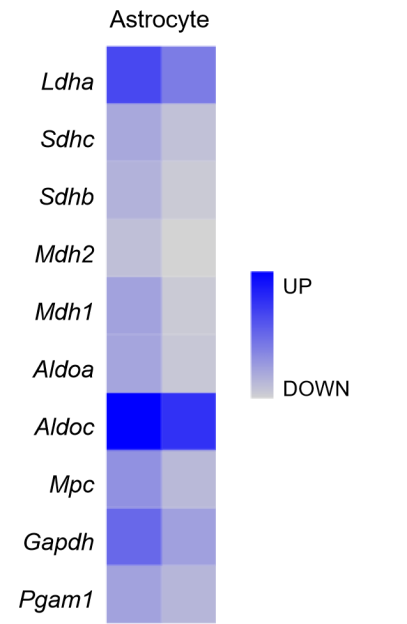


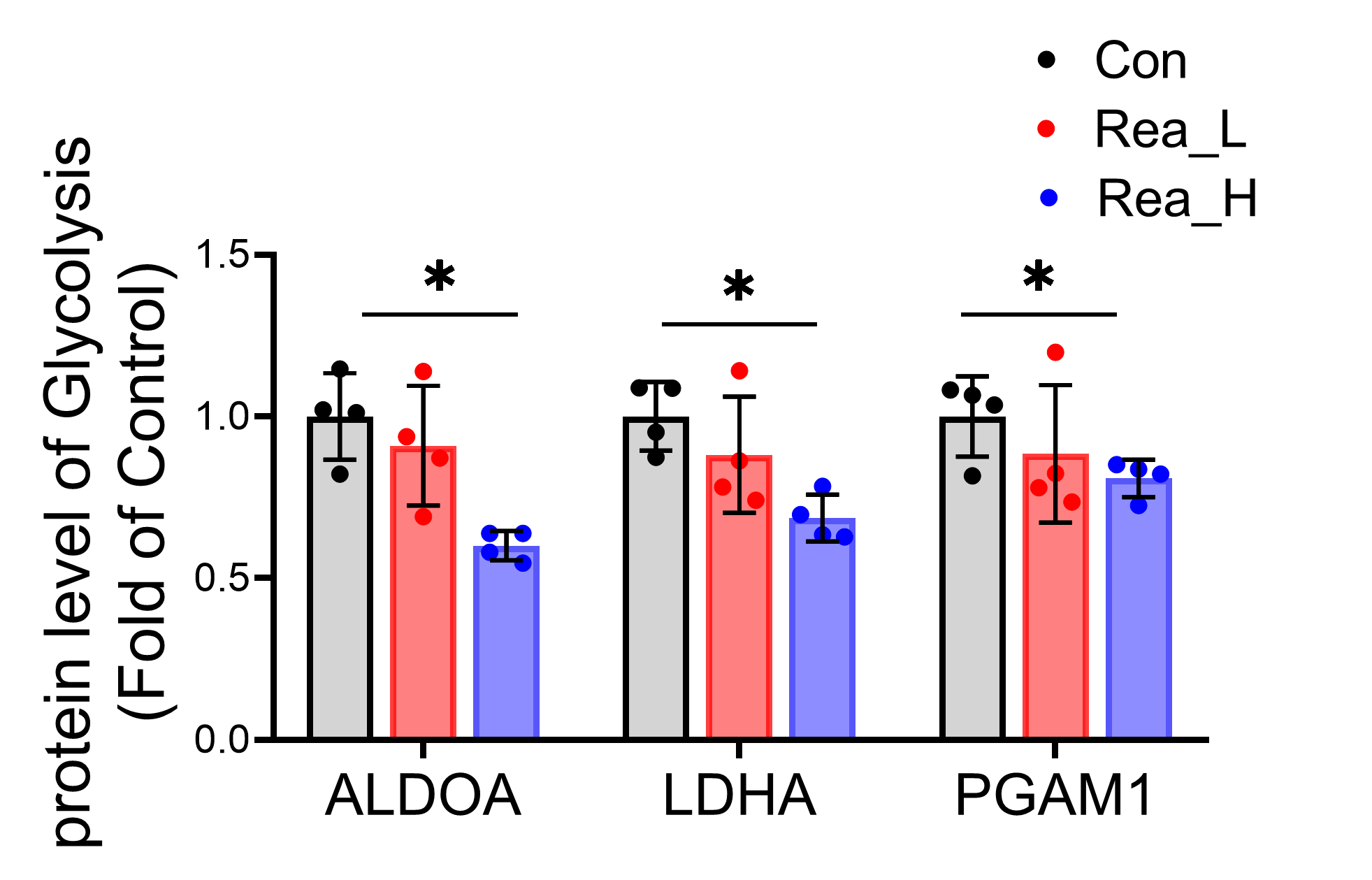


**Figure S6. Realgar inhibits astrocyte glycolysis and promotes astrocyte polarization toward the A1 phenotype.** (**A**) T-SNE descending clustering of each cell cluster. (**B**) Marker gene violin maps for each cell type. (**C**) Marker gene bubble plots of two cell subtypes of astrocytes. (**D**) Quantitative analysis of the protein expression of GFAP, C3,CCR5,CCL3 in the frontal lobe (n=4). (**E**) Heatmap of glycolysis-related gene expression in astrocytes. (**F**) Quantitative analysis of the protein expression of ALDOA, LDHA,PGAM1in the frontal lobe (n=4). Compared with the Con group, **P* < 0.05; the data are expressed as the mean±SD.


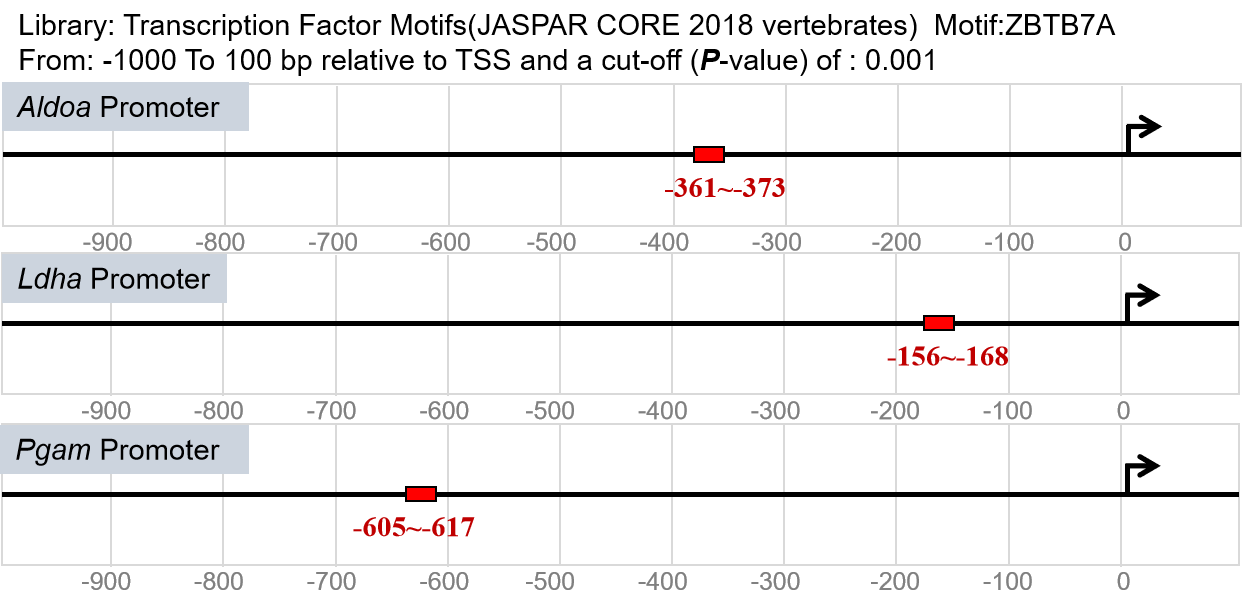


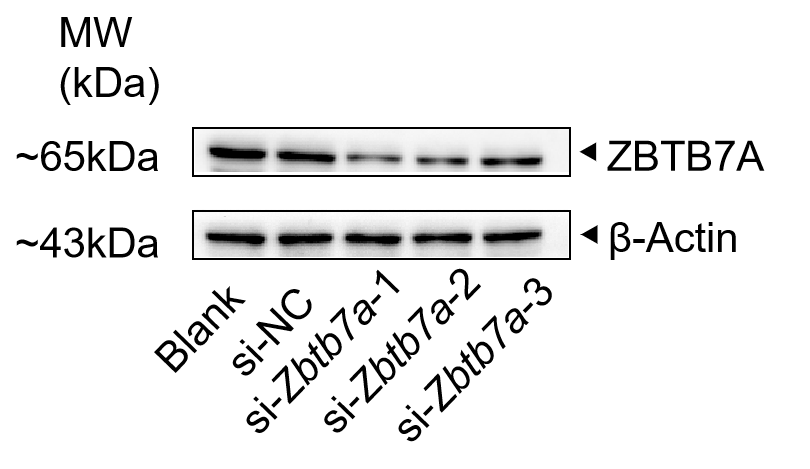

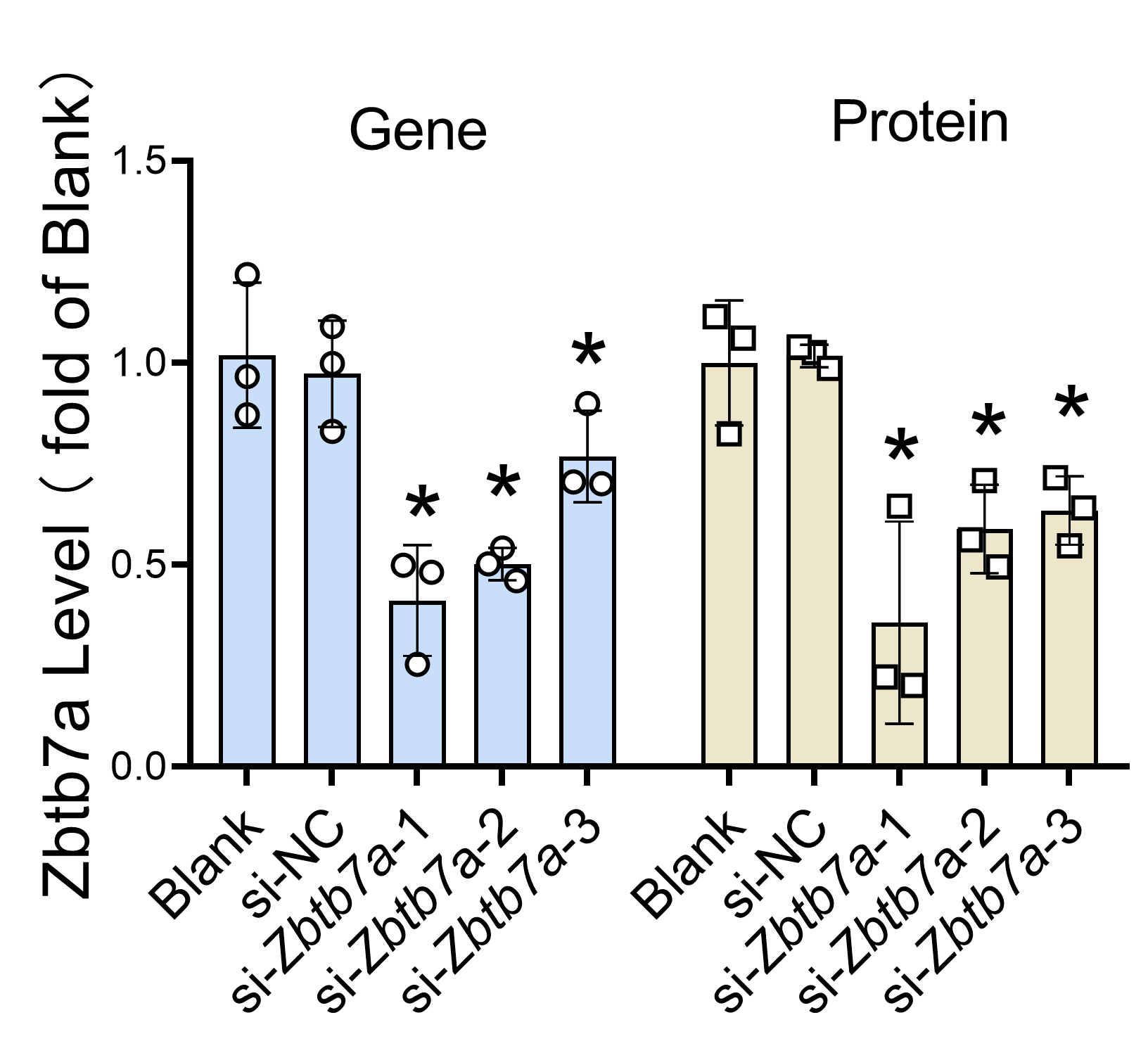

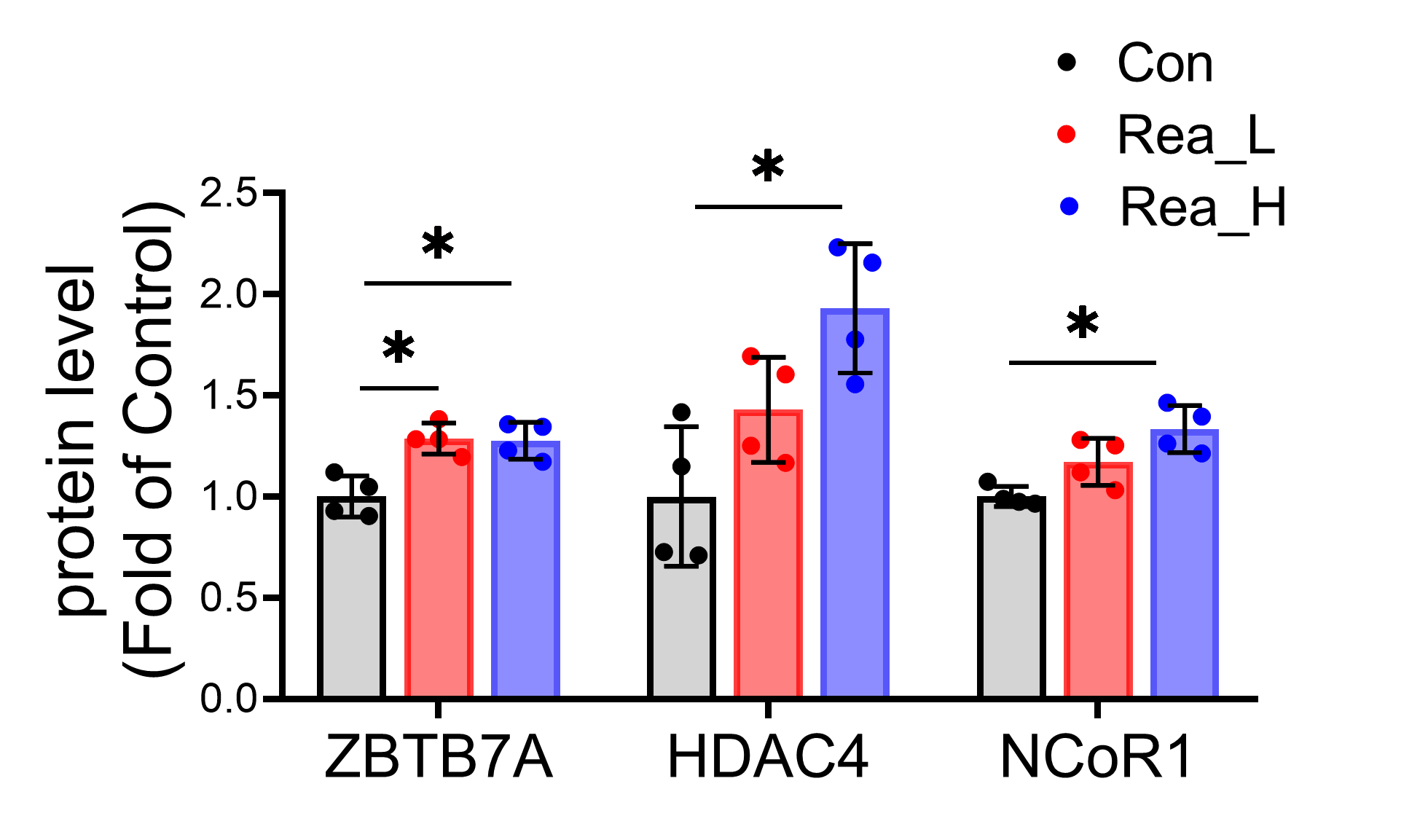

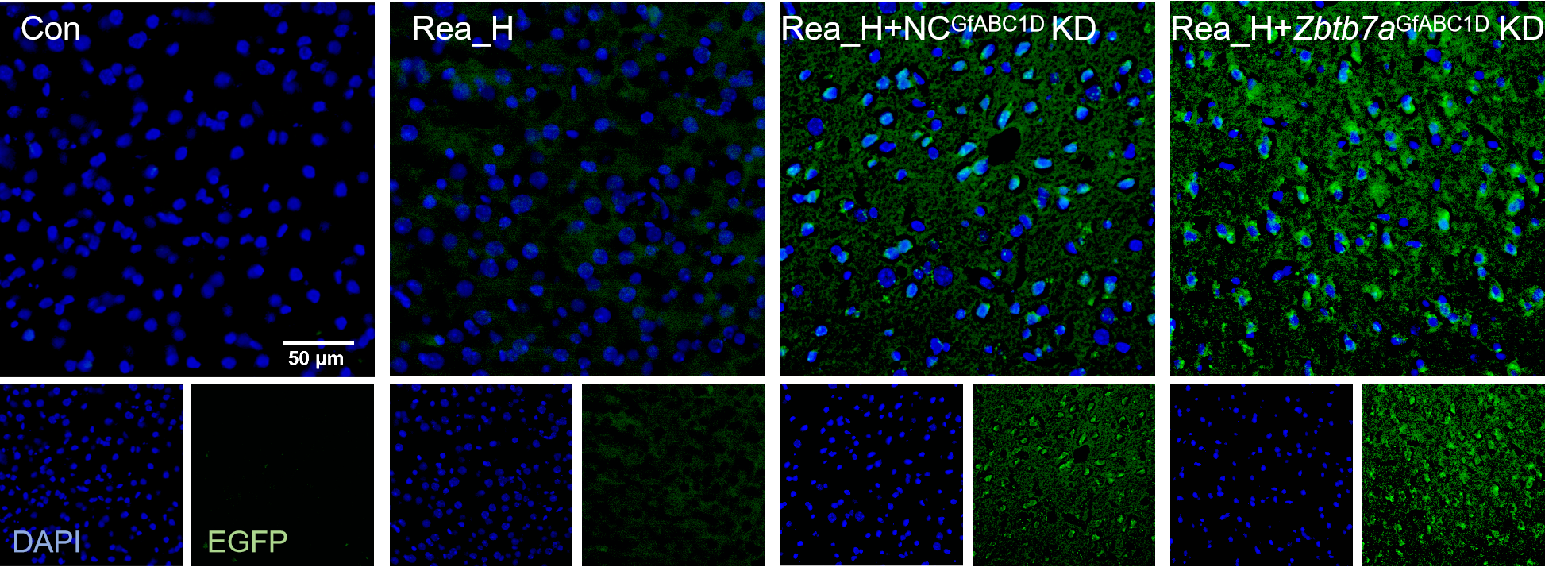

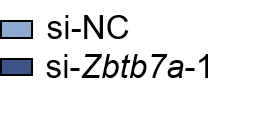


OFT

EPM


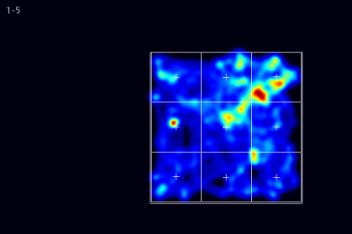

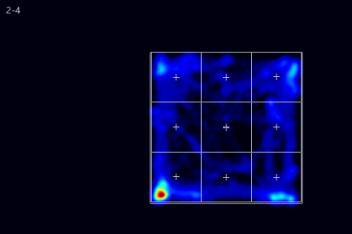

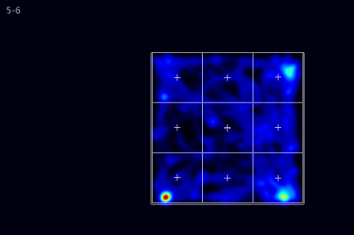

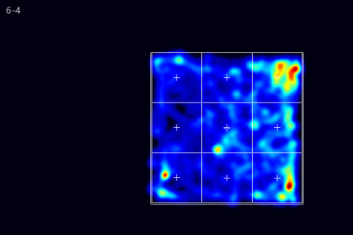

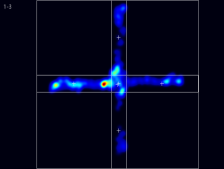

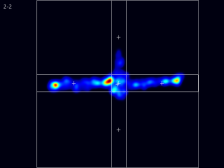

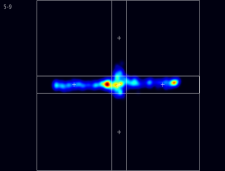

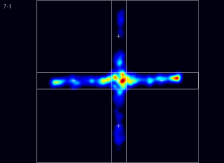

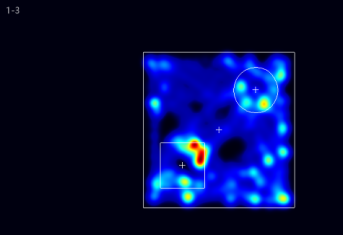

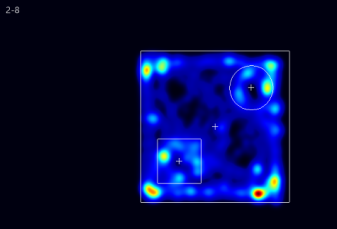

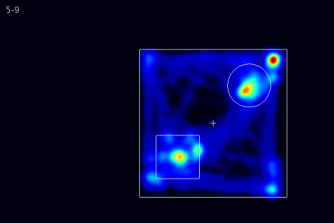

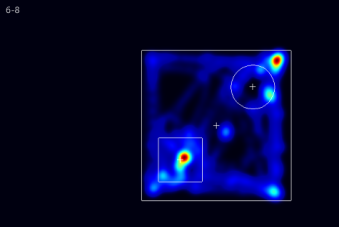


NOR

Con Rea_H


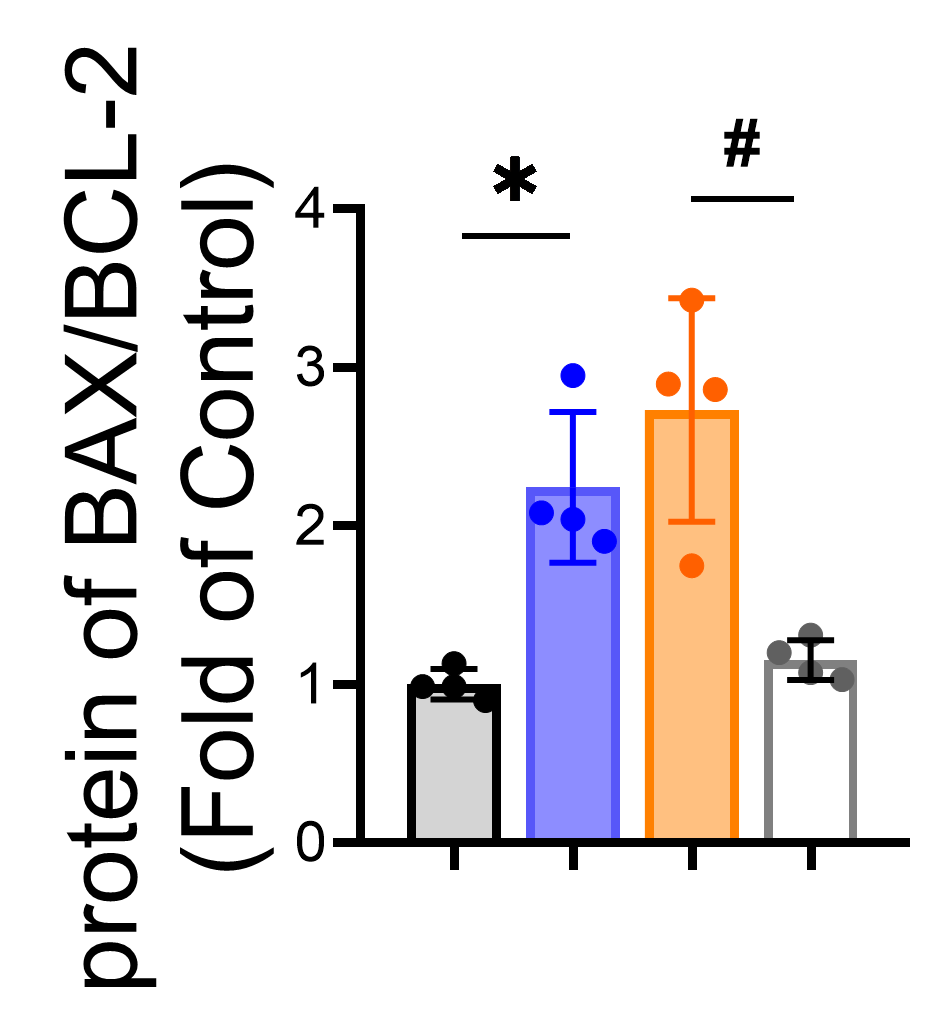

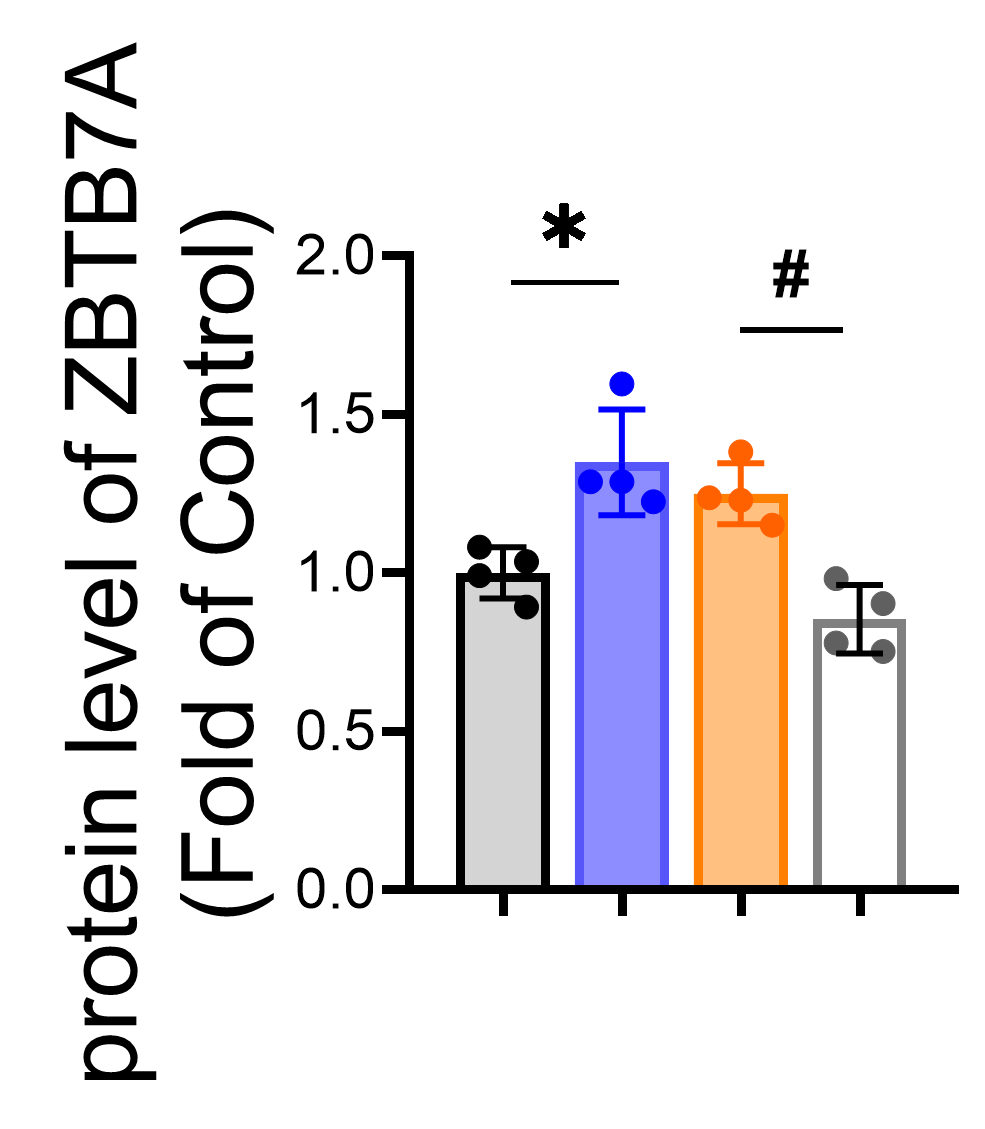

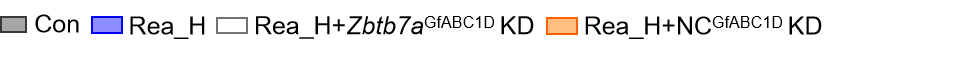


A

B

C

D

E

G

F

H

I


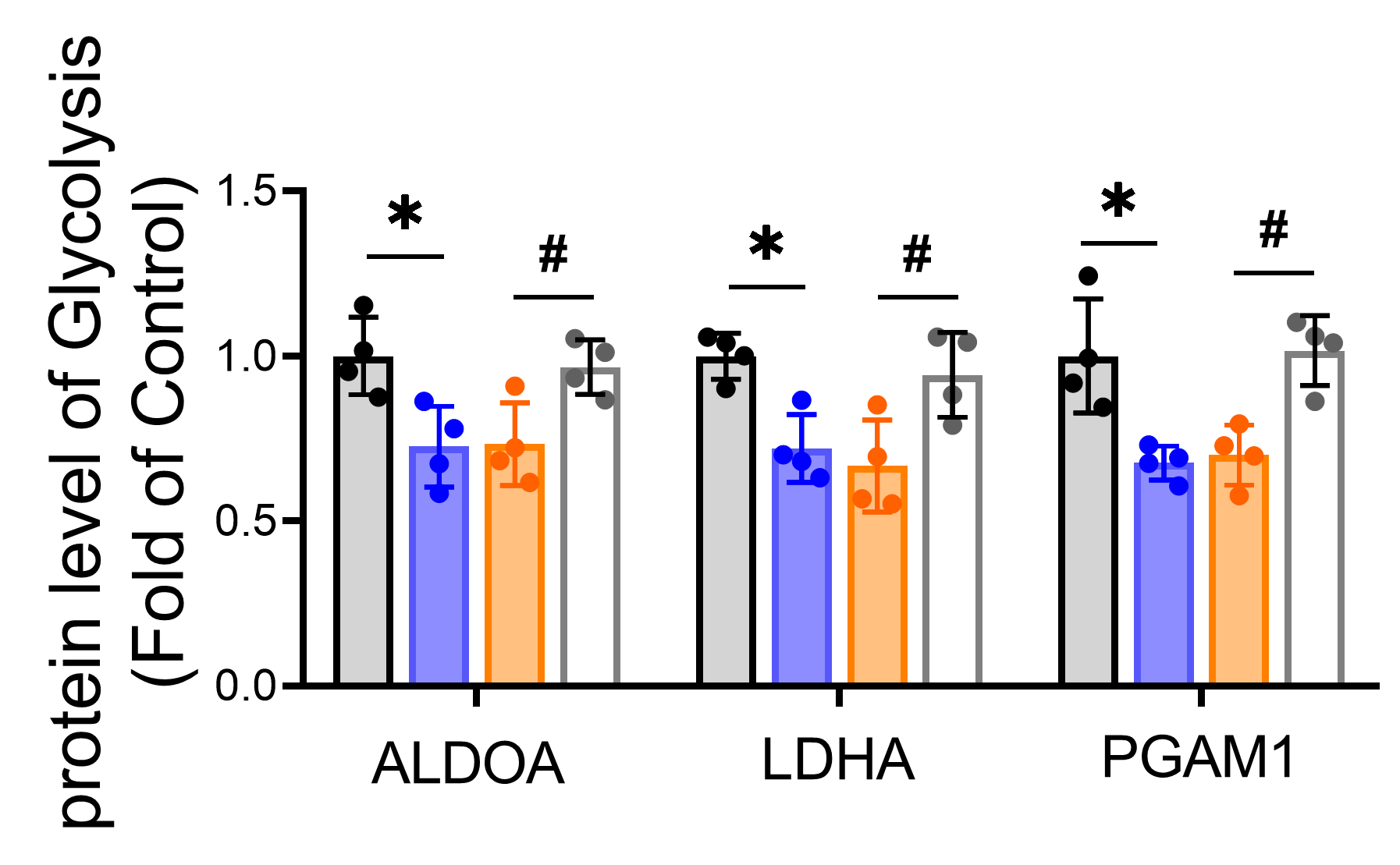


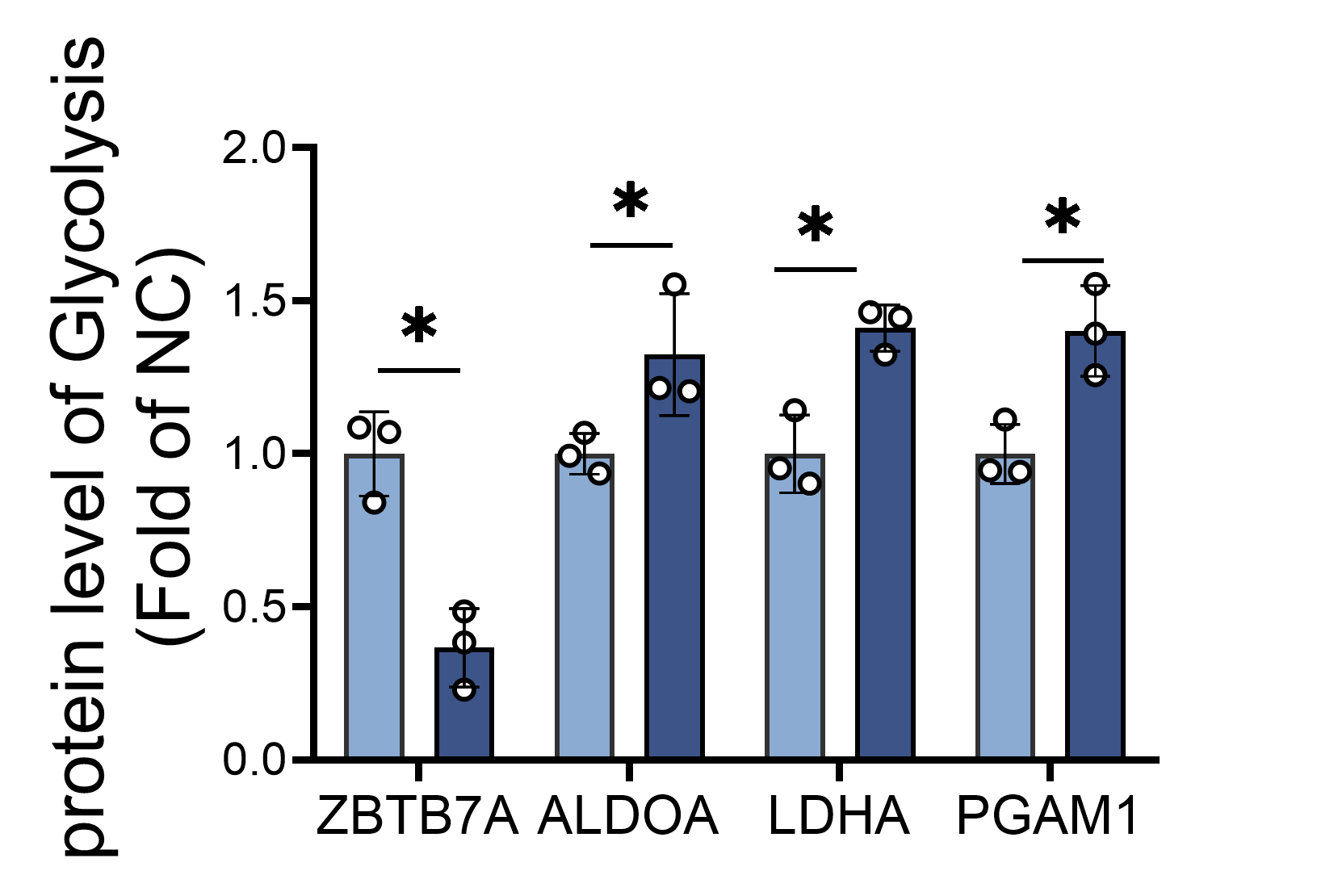


Rea_H+

NC^GfABC1D^ KD

Rea_H+

*Zbtb7a*^GfABC1D^ KD

**Figure S7. ZBTB7A targets and inhibits astrocyte glycolysis during realgar-induced CNS toxicity.** (**A**) ZBTB7A binding sites to Aldoa, Ldhb, and Pgam1 promoters. (**B**) siRNA transfection, ZBTB7A mRNA and protein expression levels in C8-D1A cells (n=3). compared with the Blank group, **P* < 0.05. (**C**) Quantitative analysis of the protein expression of ZBTB7A, ALDOA, LDHA, PGAM1in the C8-D1A cell(n=3). Compared with the NC group, **P* < 0.05.(**D**) Quantitative analysis of the protein expression of ZBTB7A, HDAC4, NCoR1 in the frontal lobe (n=4). (**E**) AAV fluorescence (green) in the frontal lobe of mice. scale bar, 50 μm. (**F**) Quantitative analysis of the protein expression of ZBTB7A in the frontal lobe (n=4).(**G**) Mouse locomotion trajectories in Novel object recognition, Open field test, Elevated plus maze experiment. (**H**) Quantitative analysis of the protein expression of BAX, BCL2 in the frontal lobe (n=4). (**I**) Quantitative analysis of the protein expression of ALDOA, LDHA, PGAM1 in the frontal lobe (n=4).Compared with the Con group, **P* < 0.05; compared with the Rea_H+NC^GfABC1D^ KD group, #*P* < 0.05; the data are expressed as the mean±SD.


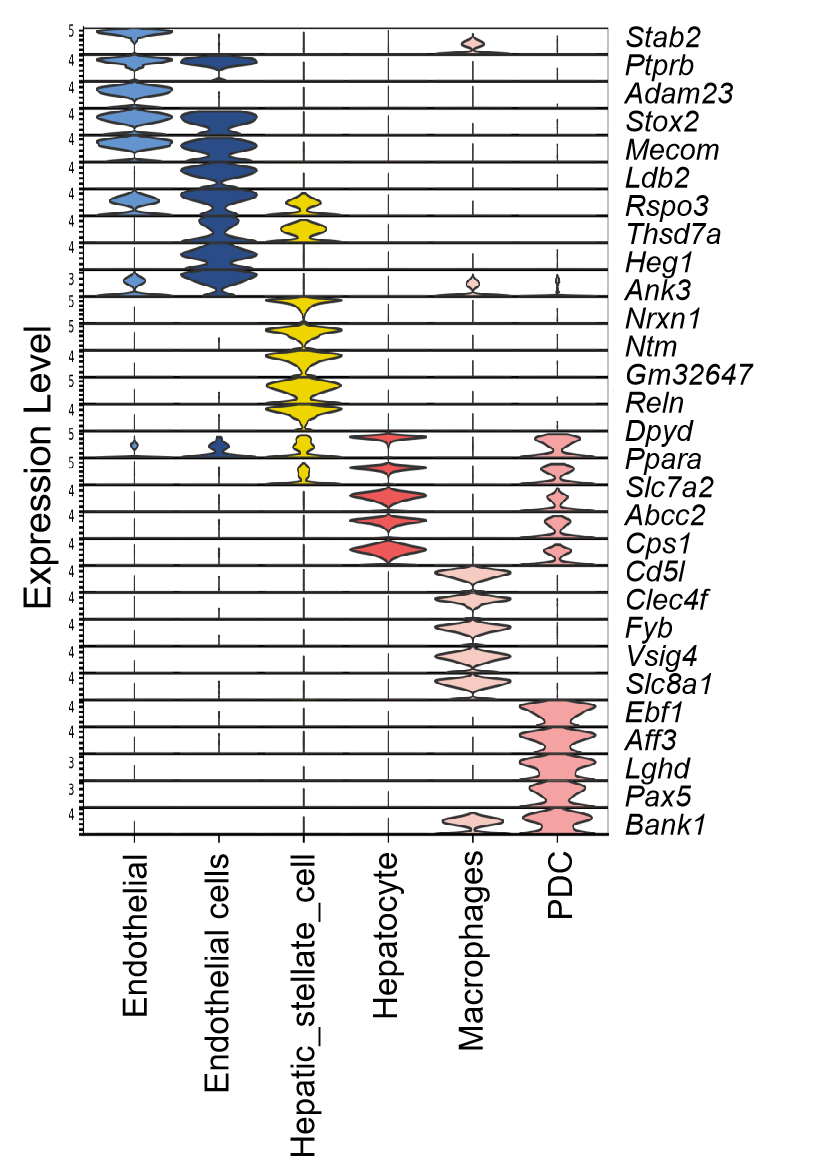


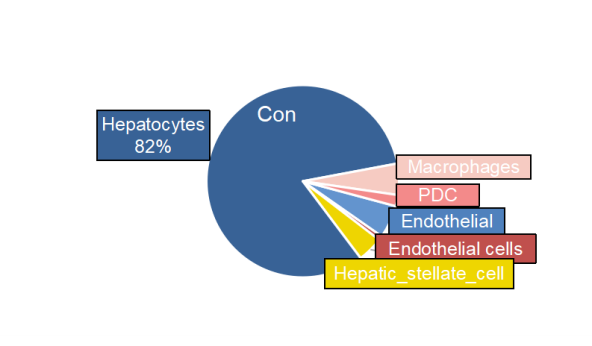

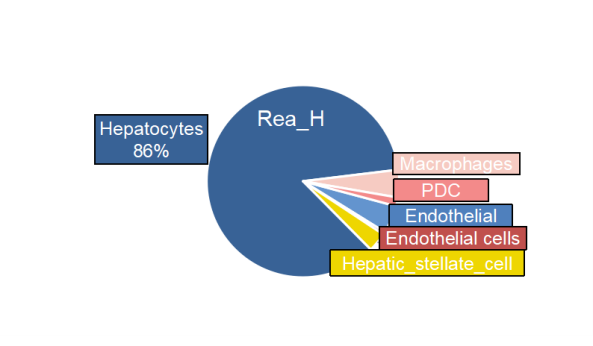


E

D


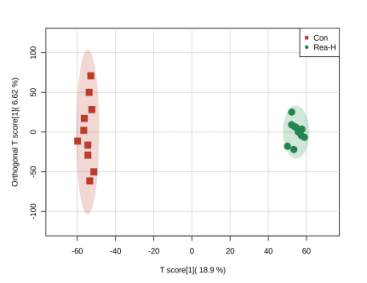

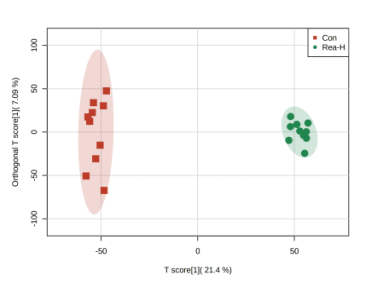

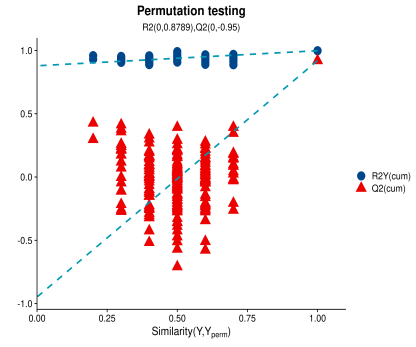

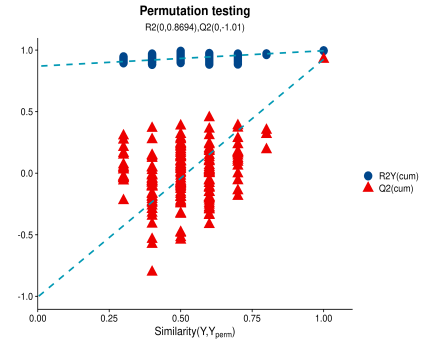


NEG

POS

NEG

POS


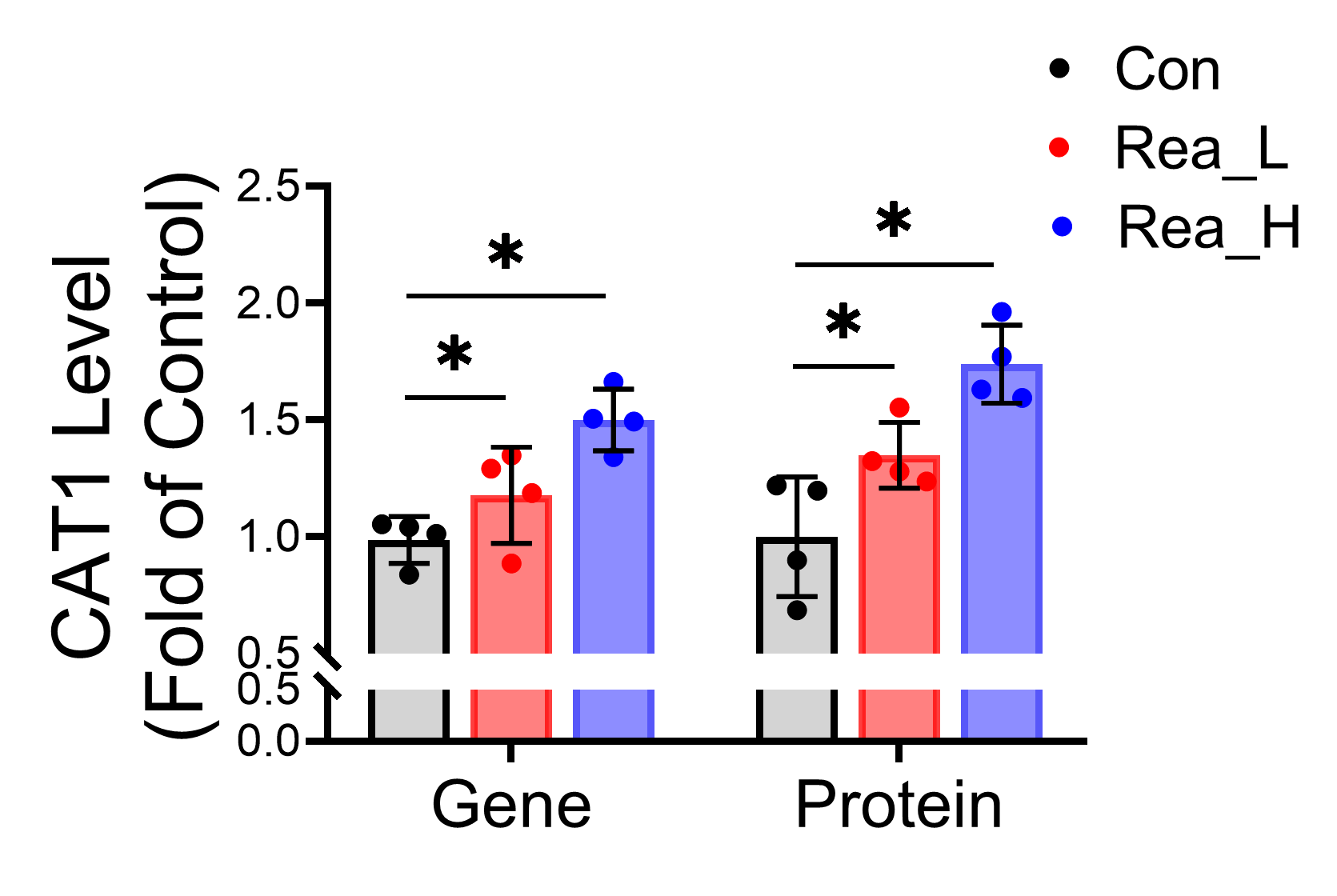

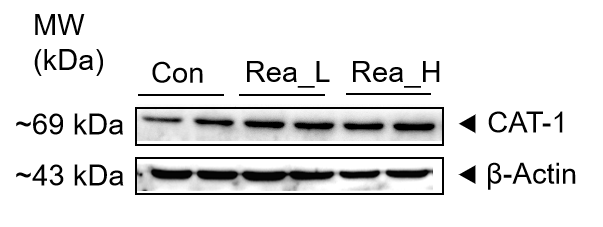

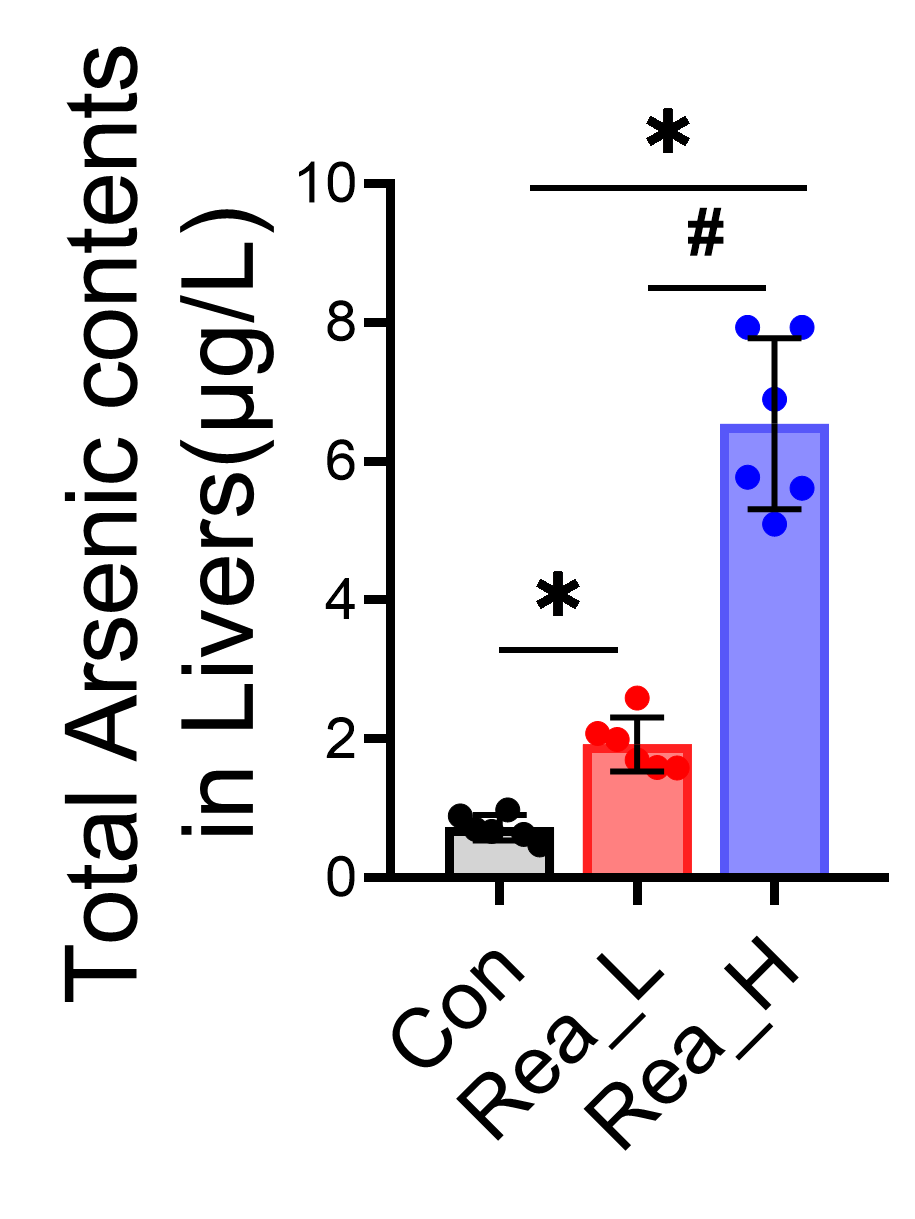


A

B

C

G

F

H


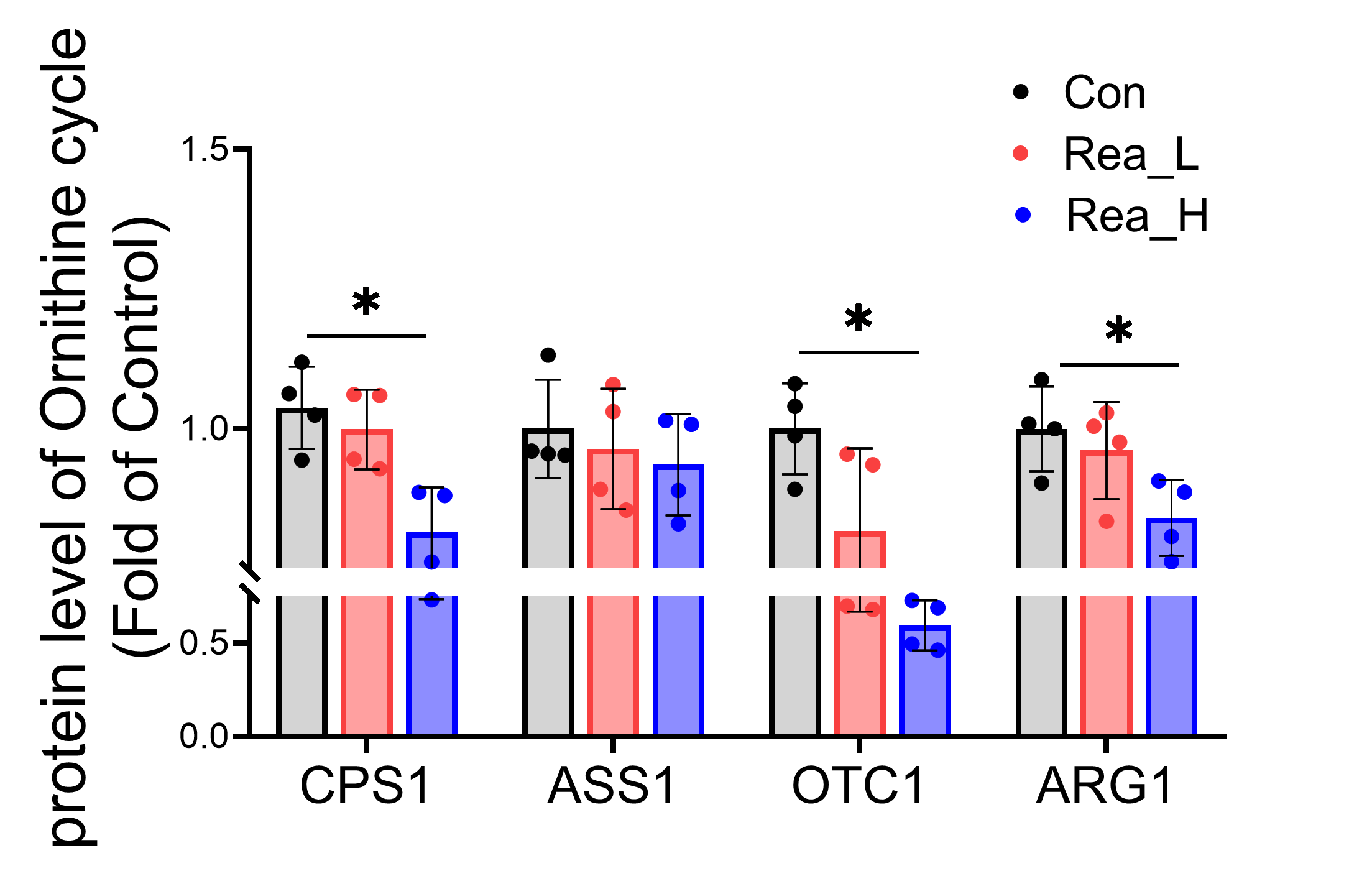


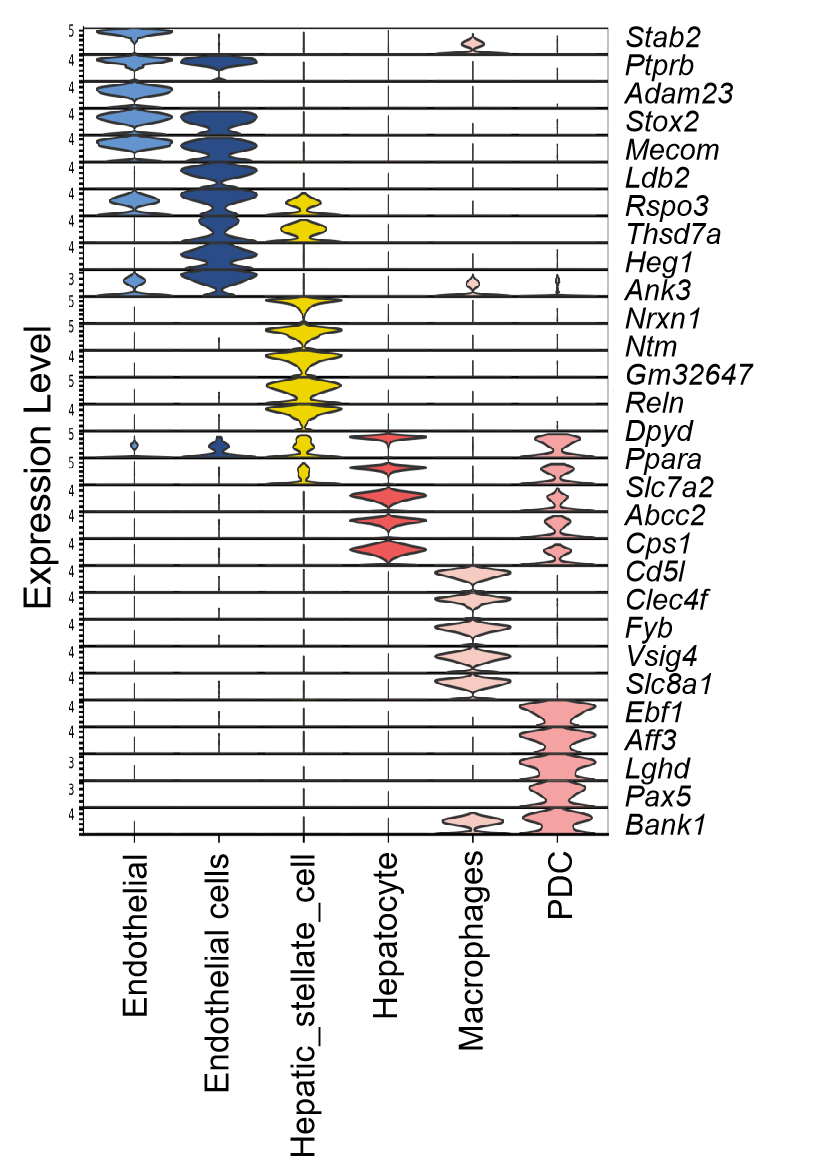


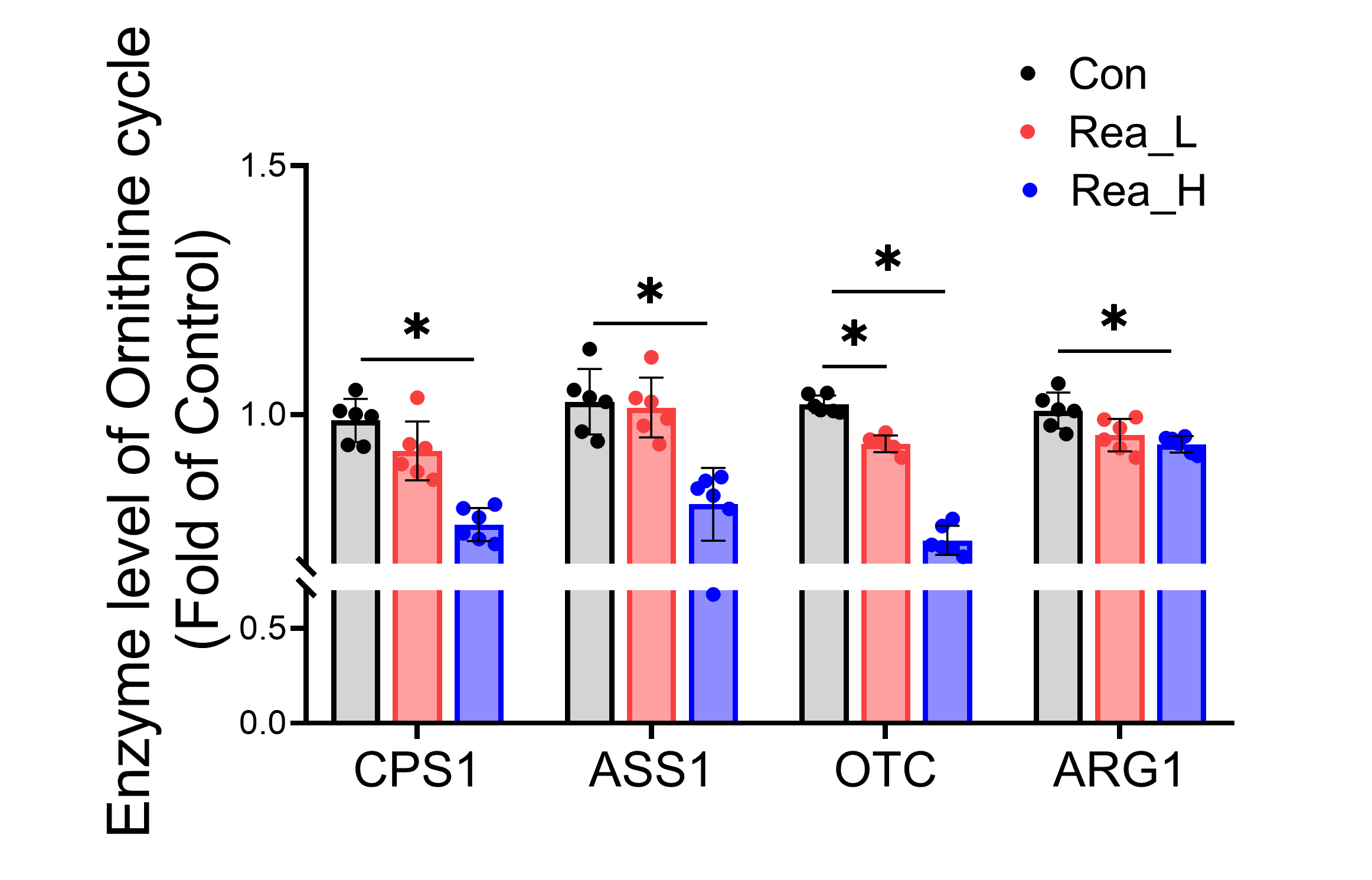


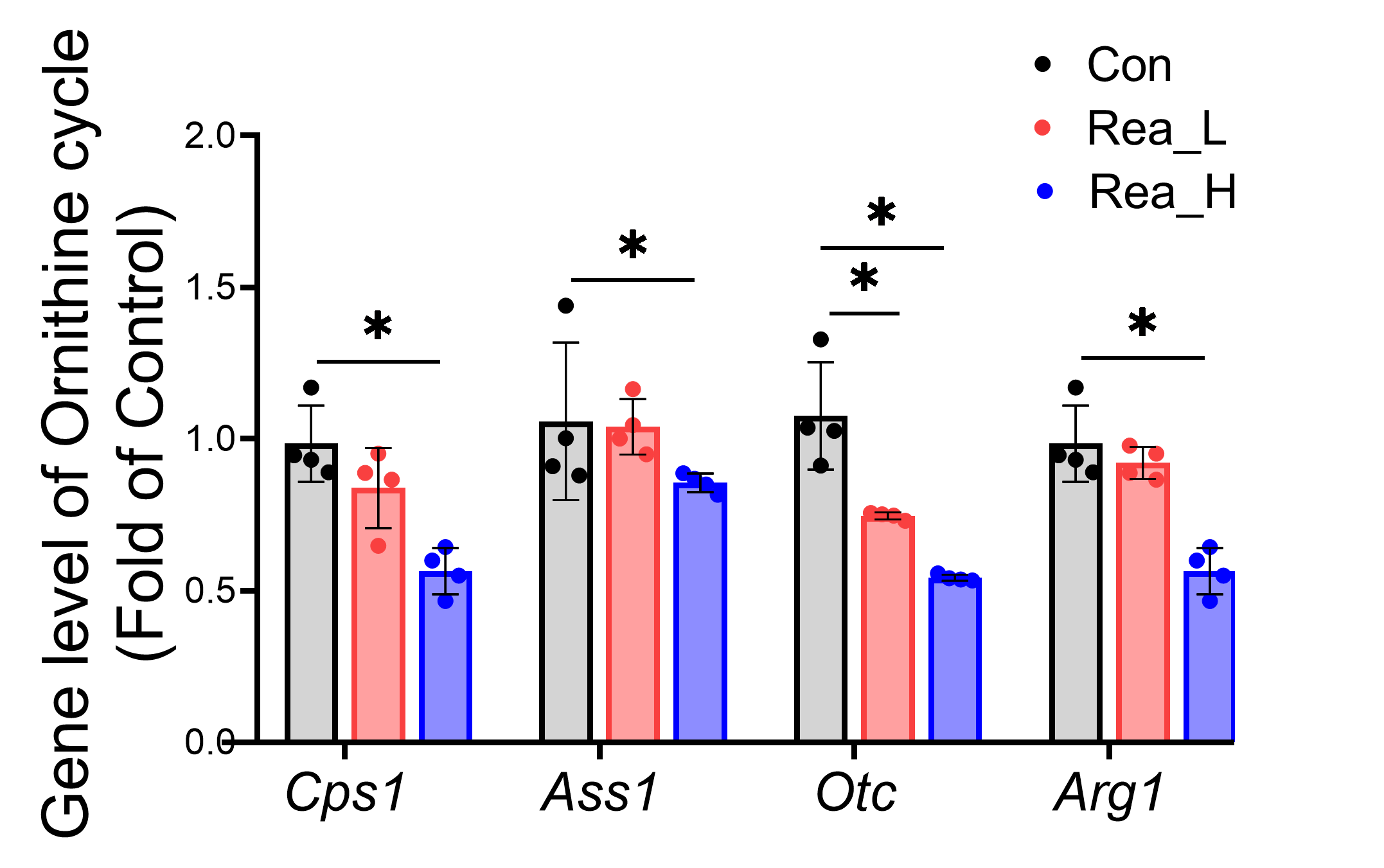


**Figure S8.Inhibition of hepatic OTC expression by realgar results in ornithine accumulation in the liver, blood, and frontal lobe.** (**A**) Plot of OPLS-DA model score and replacement test in POS and NEG modes. (**B**) Protein and mRNA expression of CAT-1 in the frontal lobe (n=4). (**C**) Total arsenic content in mouse liver (n=6). (**D**) Marker gene violin maps for each cell type. (**E**) Percentage of each cell type. (**F-H**) mRNA, protein, and enzyme expression levels of CPS1, ARG1, OTC, and ASS1 (n=4 or 6). Compared with the Con group, **P* < 0.05; the data are expressed as the mean±SD.


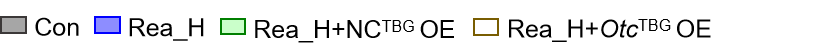

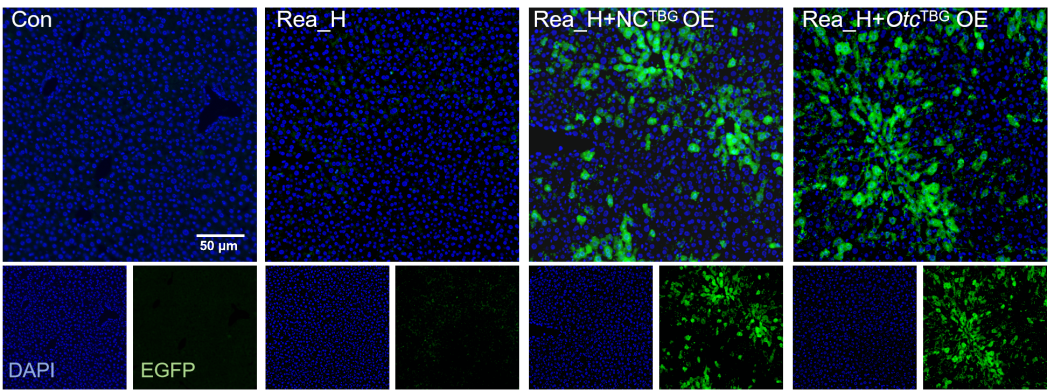


A


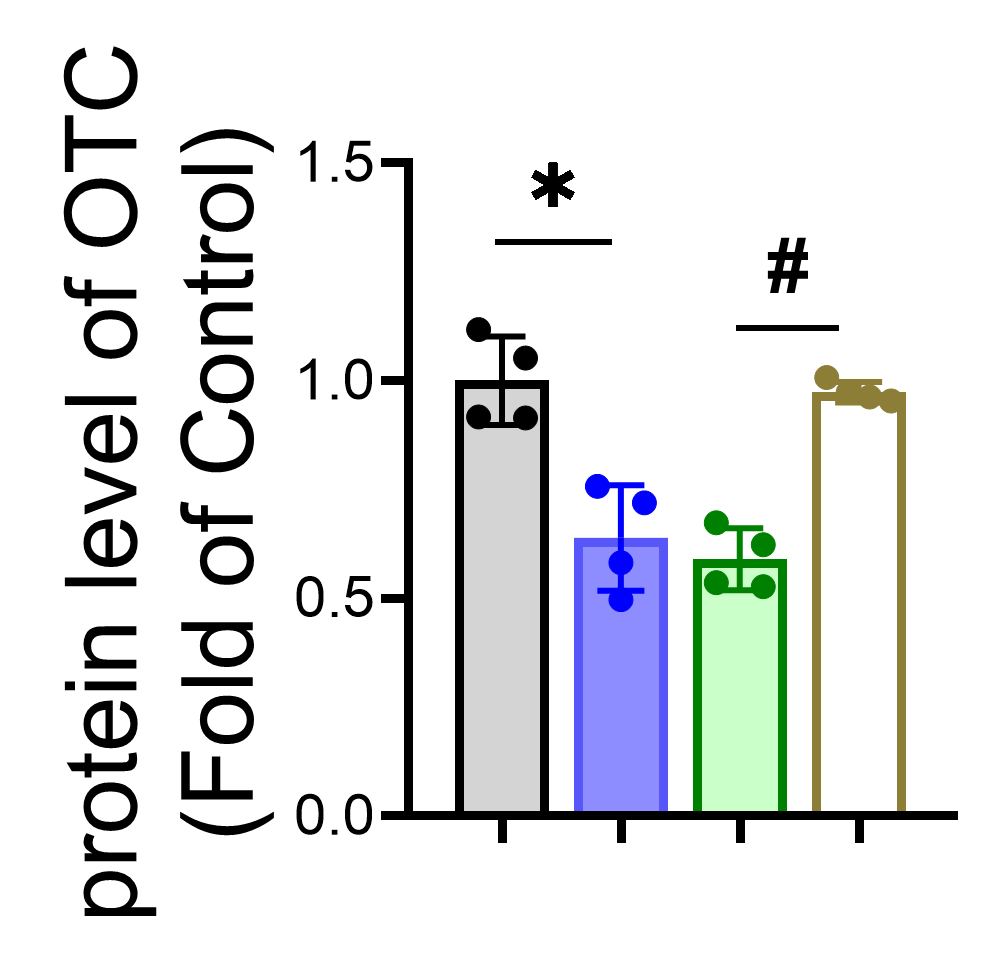

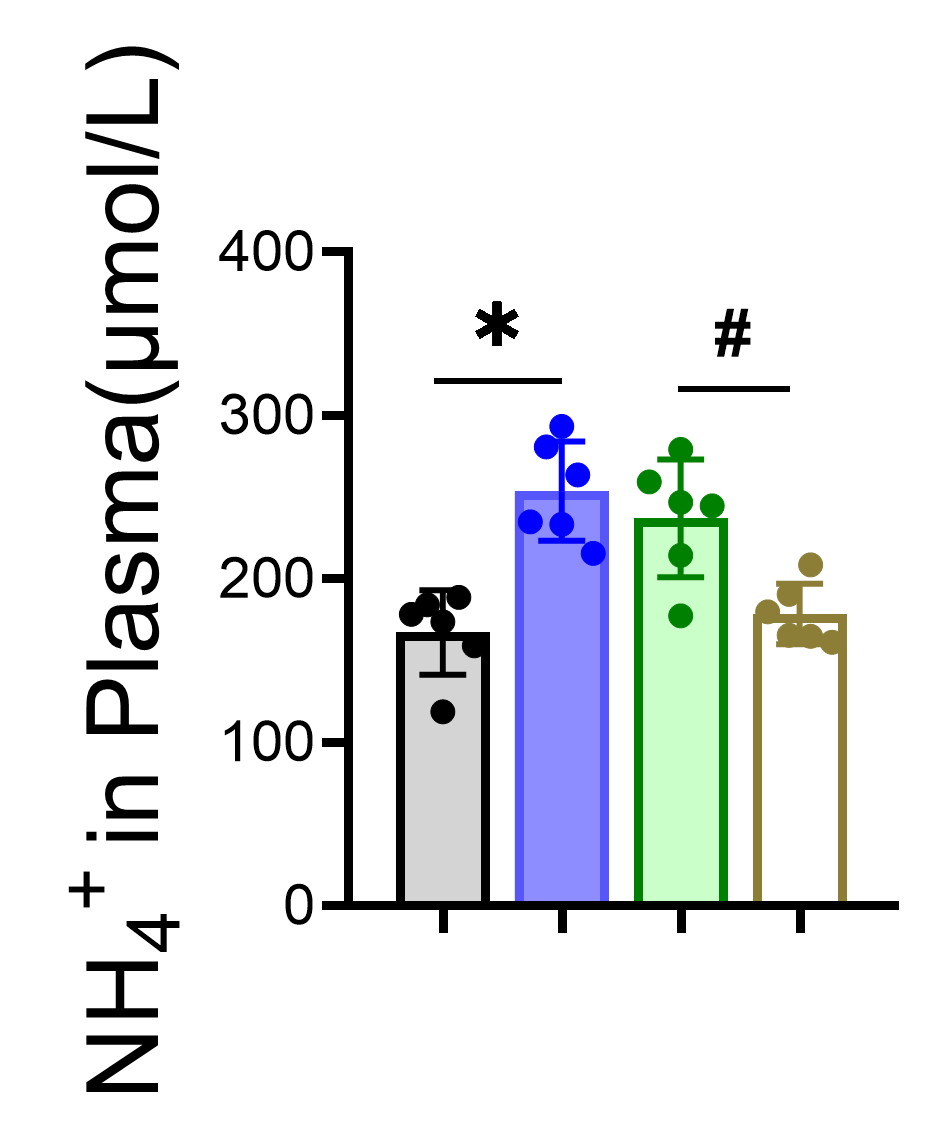

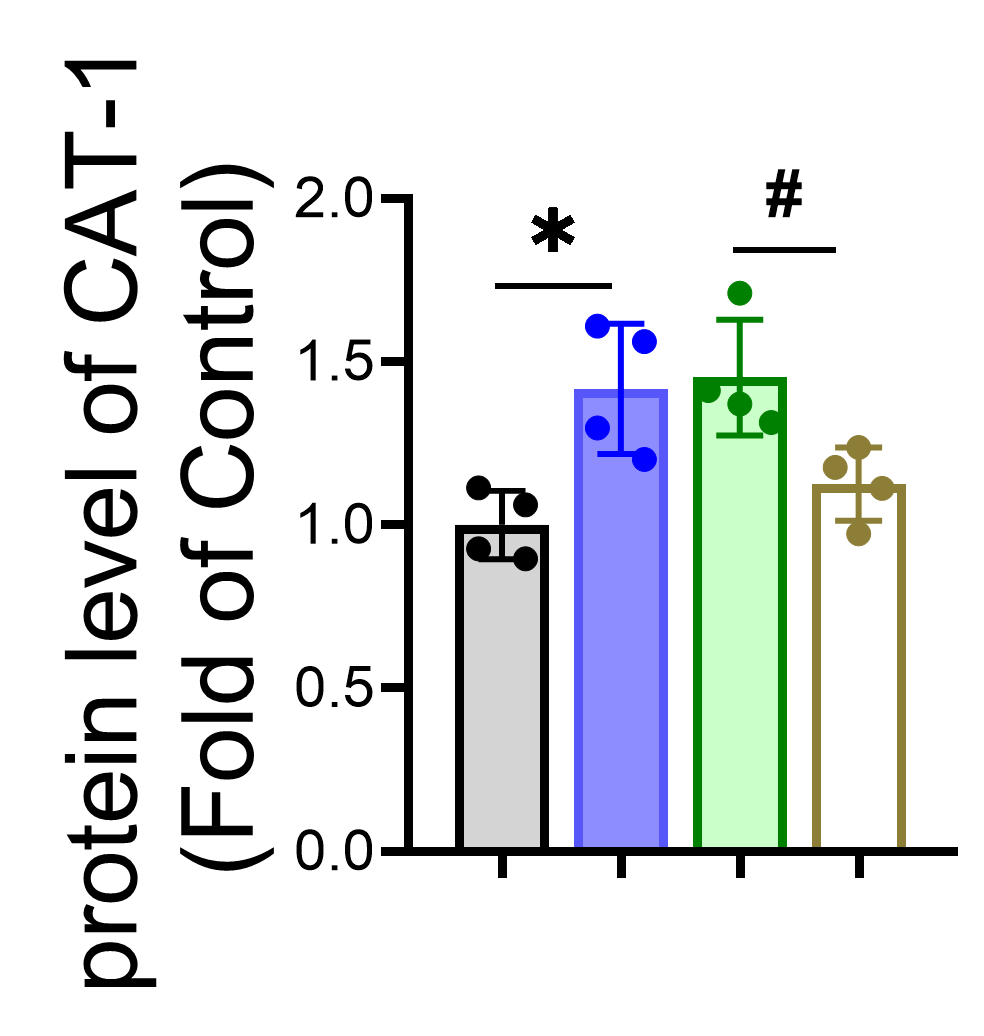


B

C

D

**Figure S9. Inhibition of hepatic OTC expression by realgar results in ornithine accumulation in the liver, blood, and frontal lobe.** (**A**) AAV fluorescence (green) in the liver of mice, scale bar, 50 μm.(**B**) Quantitative analysis of the protein expression of OTC in the liver (n=4). (**C**) Ammonia content in mouse plasma (n=6). (**D**) Quantitative analysis of the protein expression of CAT-1 in the frontal lobe (n=4). Compared with the Con group, **P* < 0.05; compared with the Rea_H+ NC^TBG^ OE group, #*P* < 0.05; the data are expressed as the mean±SD.

iAs^3+^（0-200 μM）

Orn（0-100 mM）


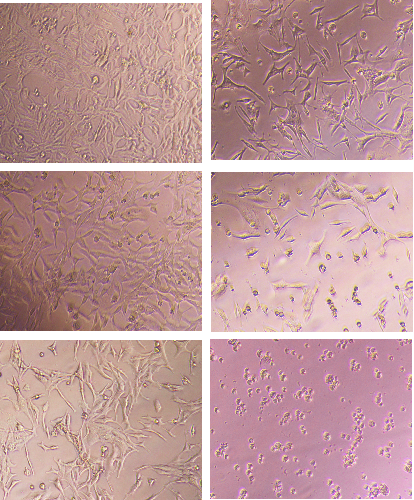

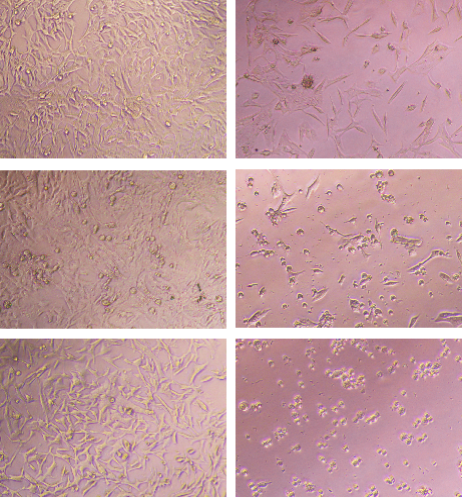

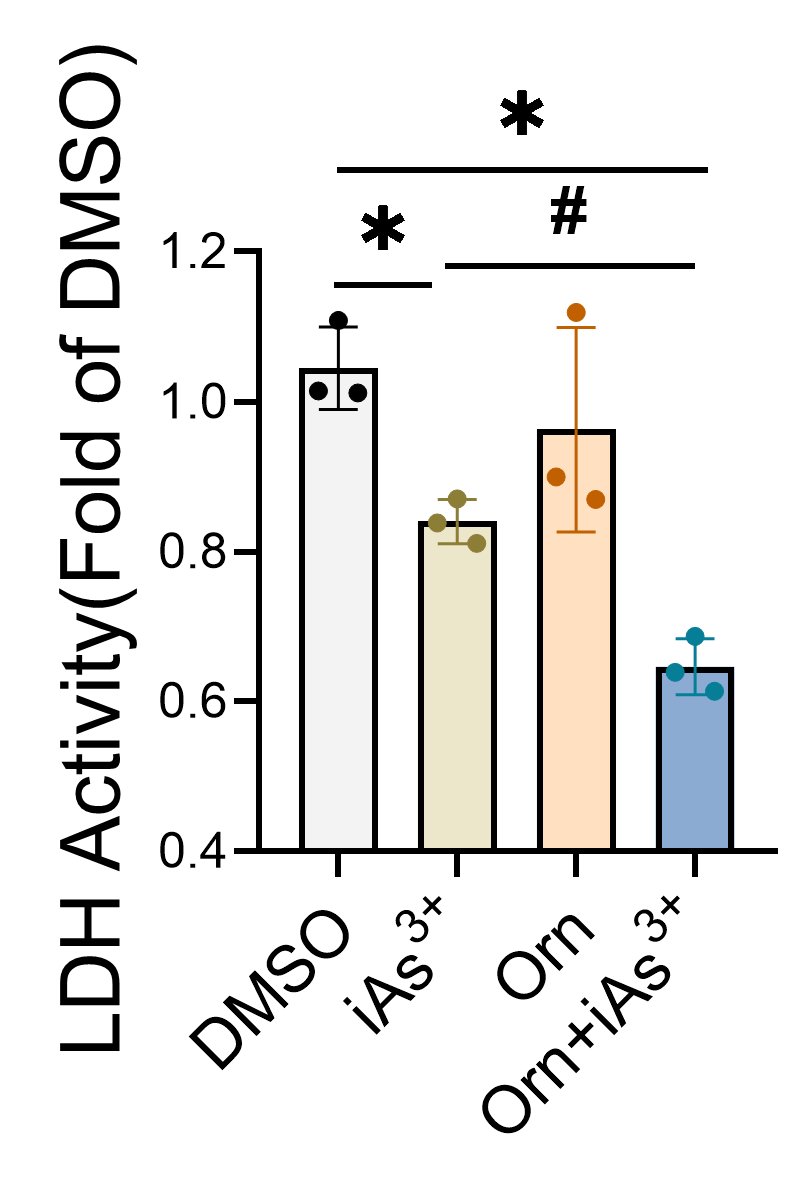

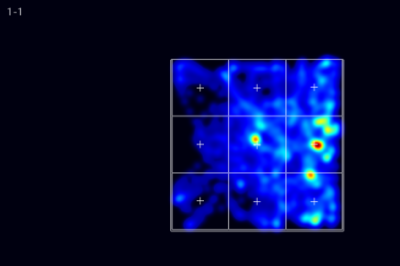

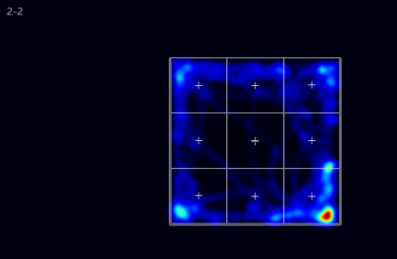

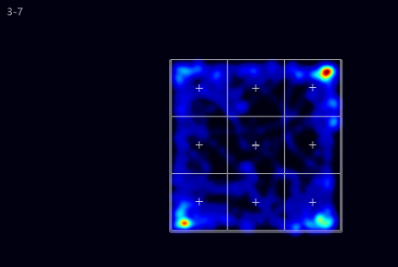

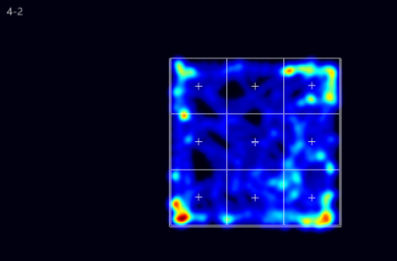

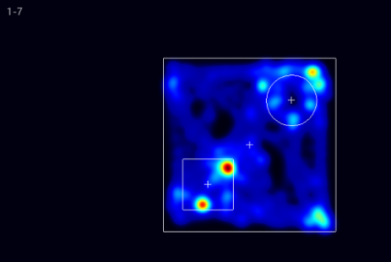

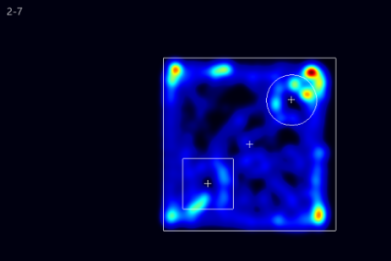

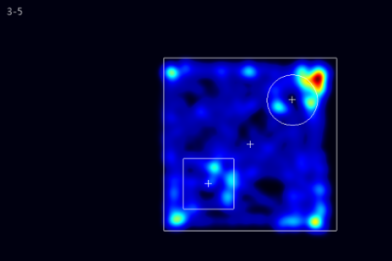

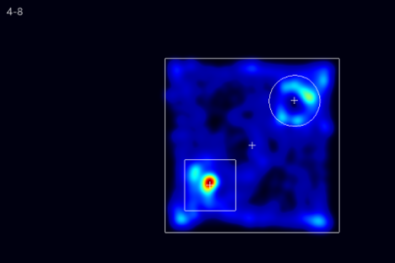

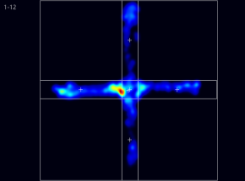

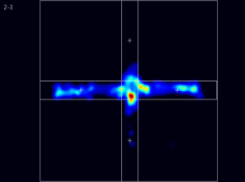

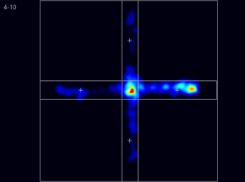

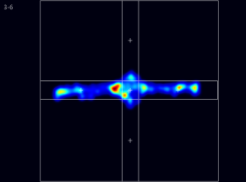


OFT

EPM

NOR

Con Rea_H


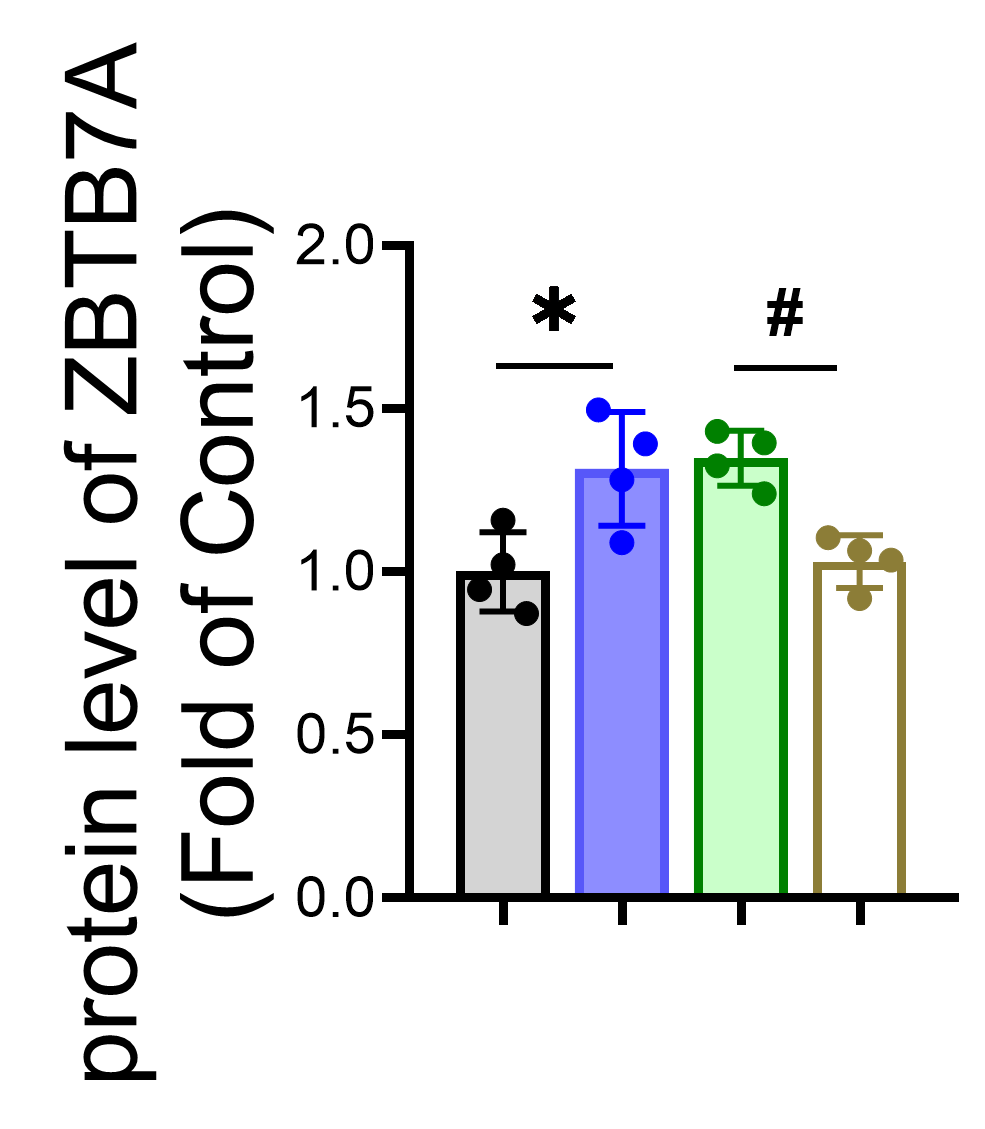

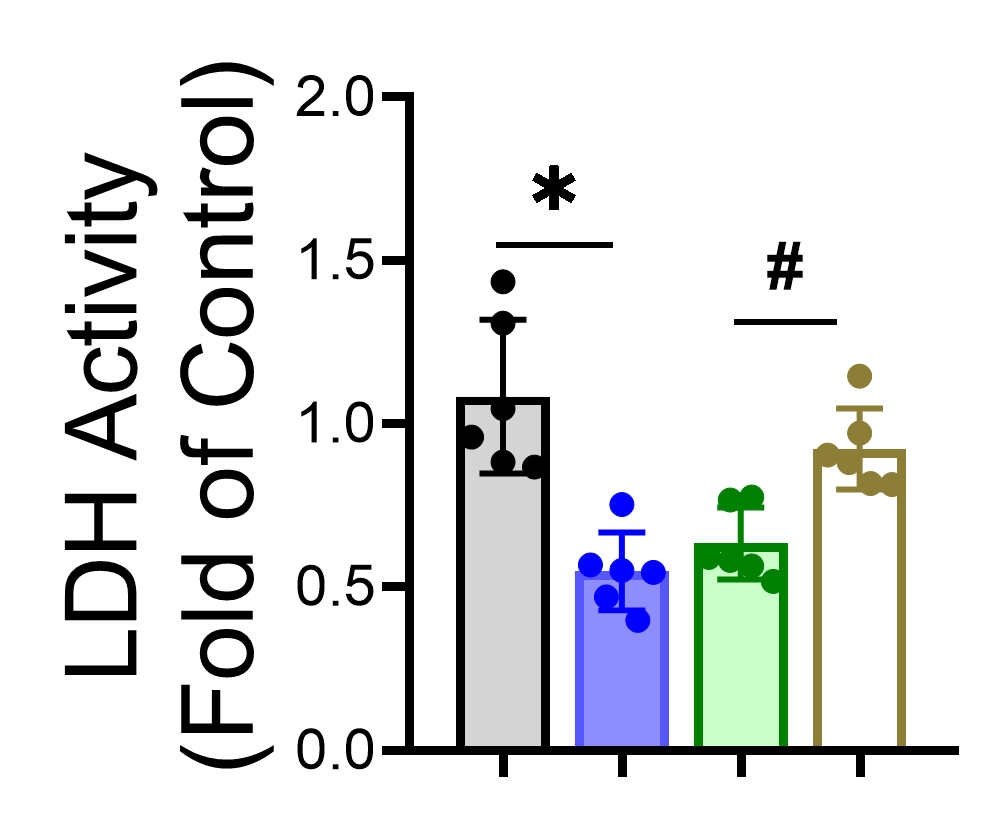

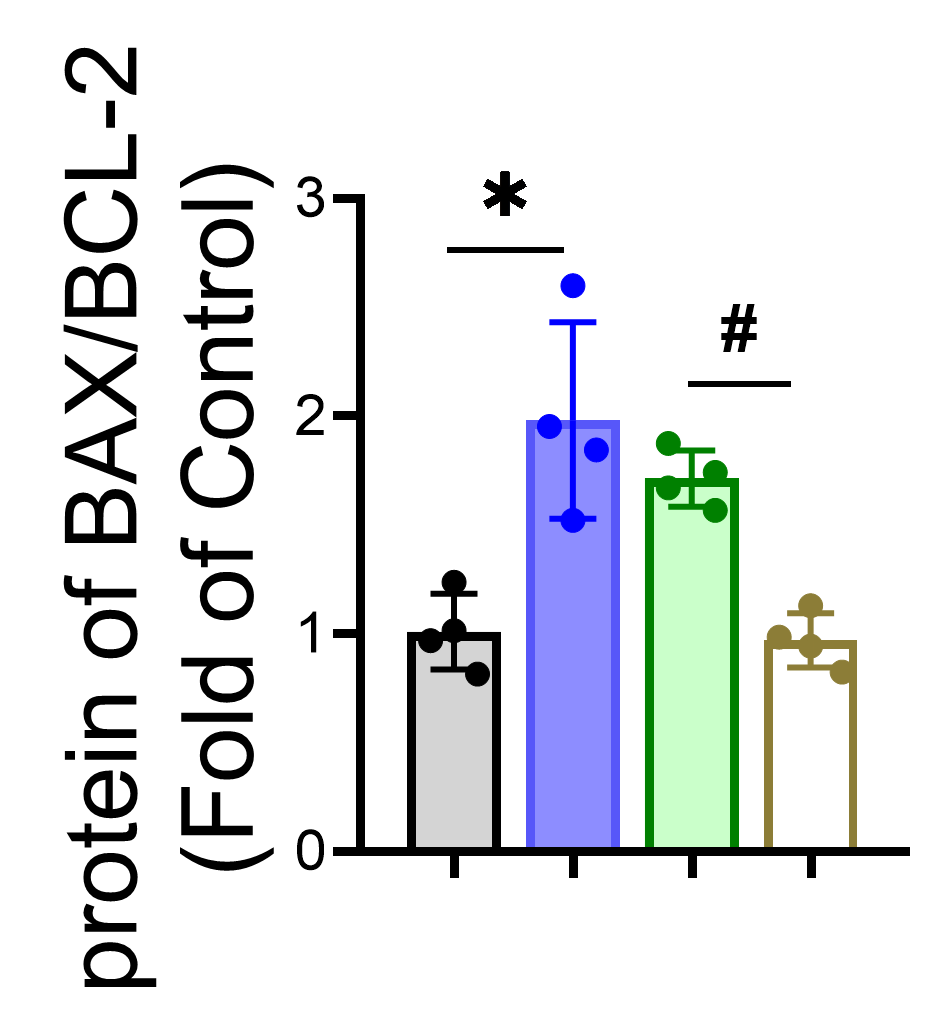

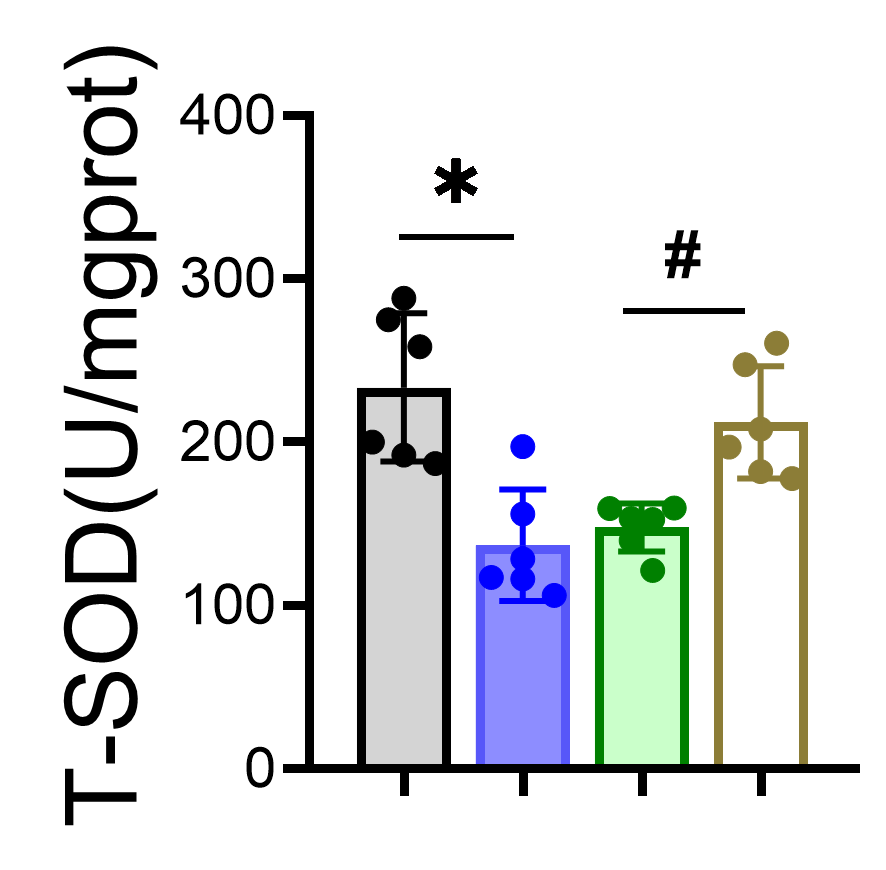

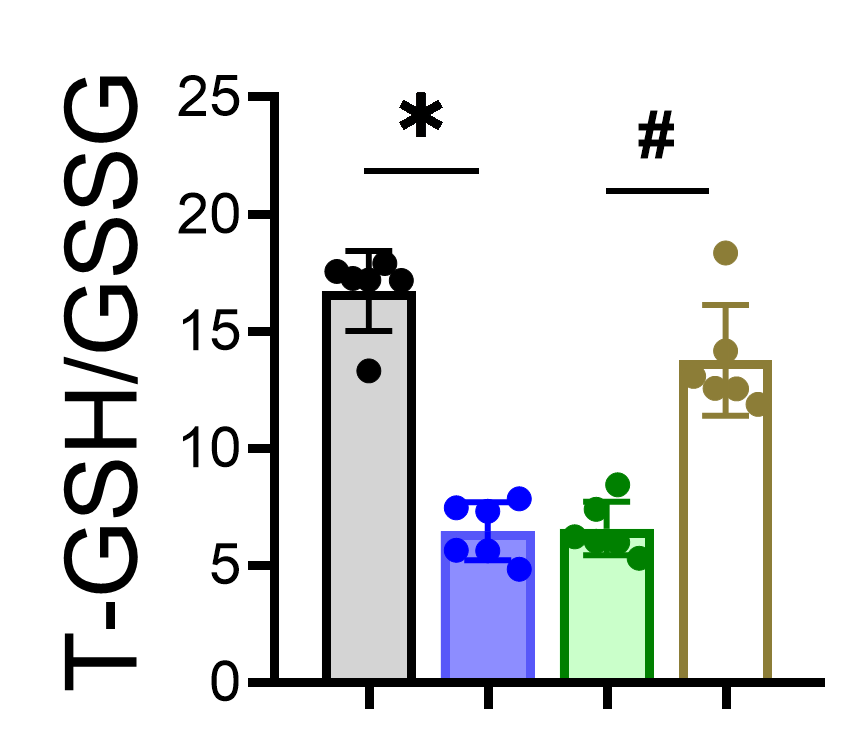

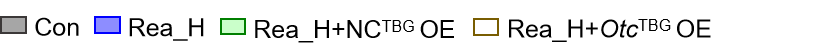


A

B

C

D

E

F

G

H


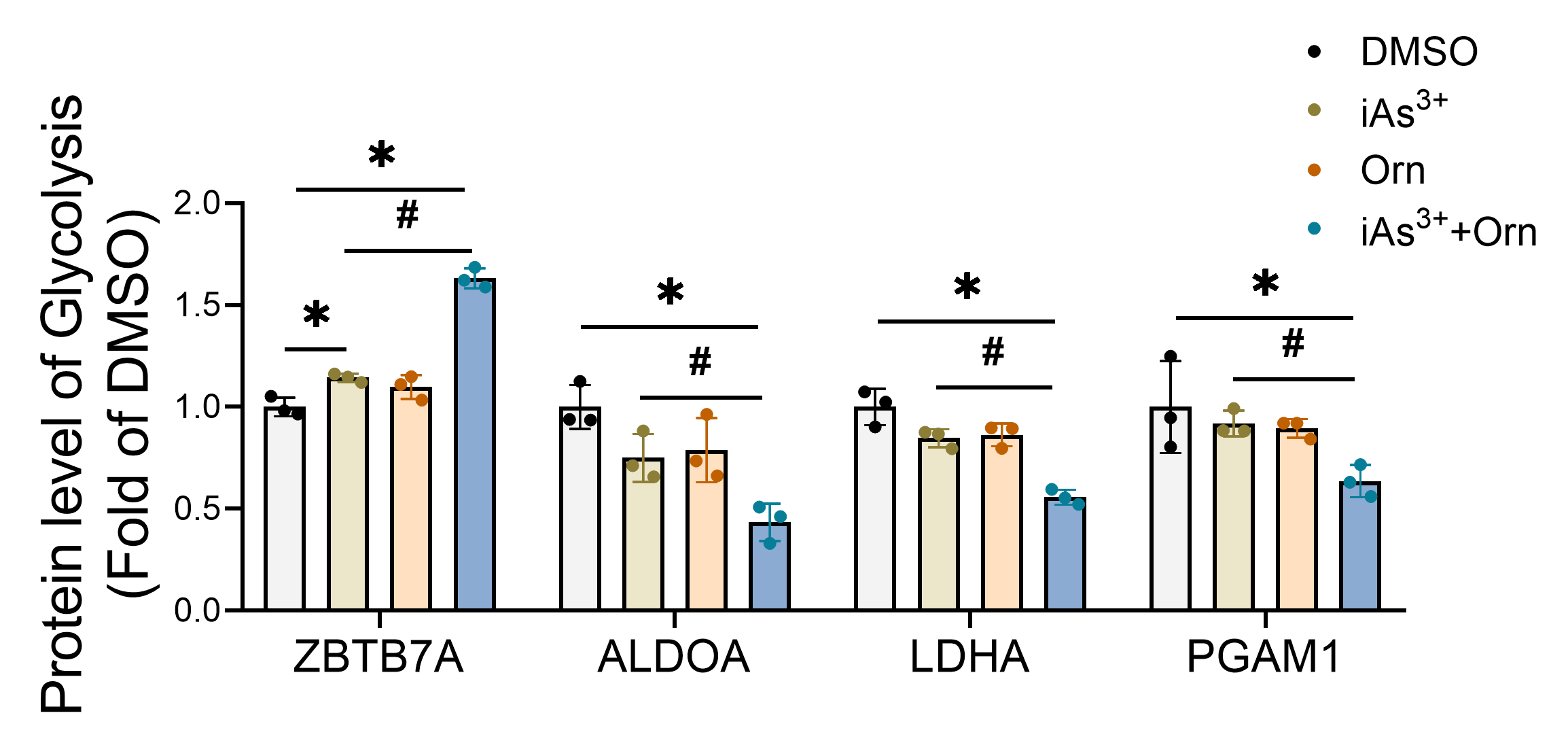


Rea_H+

*Otc*^TBG^ OE

Rea_H+

NC^TBG^ OE

**Figure S10. Ornithine-mediated regulation of ZBTB7A exacerbates the glycolysis inhibition in astrocytes induced by arsenic in realgar and increases CNS toxicity.** (**A**) Observed images of cell morphology after treatment of C8-D1A with different concentrations of iAs^3+^, Orn; (**B**) Quantitative analysis of the protein expression of ZBTB7A in the frontal lobe (n=4). (**C**) LDH content in the frontal lobe (n=6). (**D-E**) LDH content and quantitative analysis of the protein expression of ZBTB7A, LDHA, ALDOA, and PGAM1 in the C8-D1A cells (n=3). Compared with the DMSO group, *P<0.05; compared with the iAs^3+^+Orn group, #*P*< 0.05. (**F**) Movement trajectories in Novel object recognition, Open field test, Elevated plus maze experiment. (**G**) Quantitative analysis of the protein expression of BAX/BCL2 in the frontal lobe (n=4). (**H**) T-SOD content and T-GSH/GSSG ratio in the frontal lobe (n=6). Compared with the Con group, **P* < 0.05; compared with the Rea_H+NC^TBG^ OE group; the data are expressed as the mean±SD.


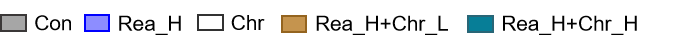

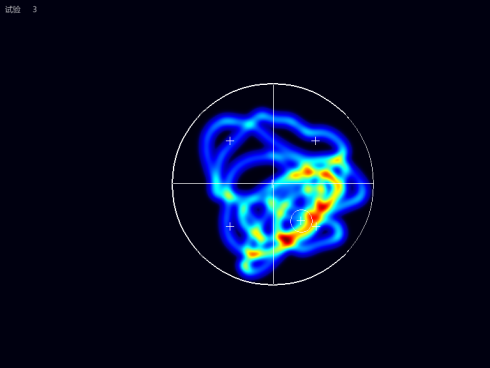

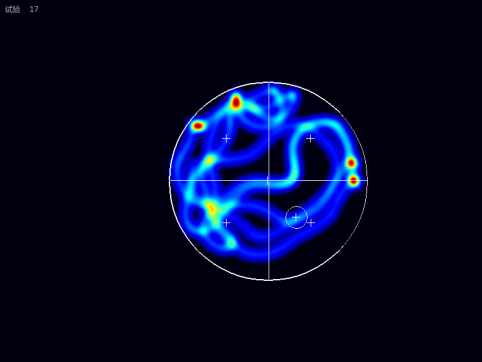

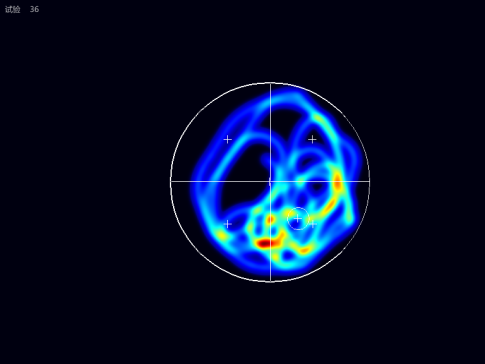

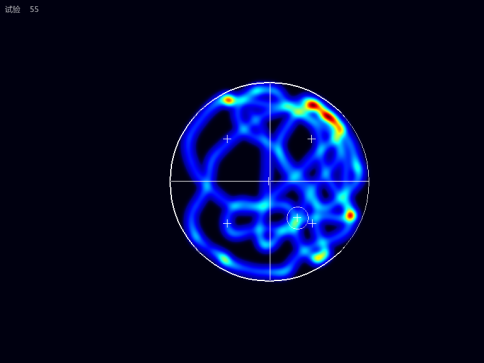

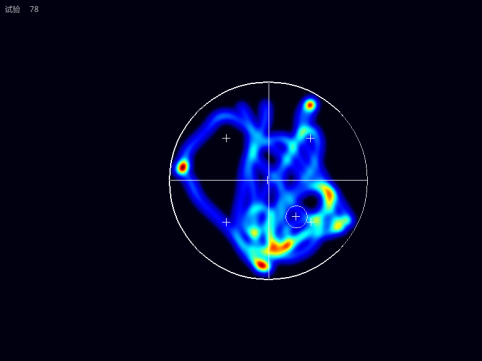


A

OFT

EPM

MWM

Con Rea_H Chr Rea_H+Chr_L Rea_H+Chr_H

B

C

D

E

F

G

H

I

J

**Fig S11. Chrysophanol protects against disorders of astrocyte glycolysis and the hepatic ornithine cycle , antagonizing realgar-induced CNS toxicity and hepatotoxicity.** (**A**) Movement trajectories in Morris water maze, Open field test, Elevated plus maze experiment. (**B**) Quantitative analysis of the protein expression of BAX/BCL2 in the frontal lobe (n=4). (**C**) Quantitative analysis of immunofluorescence double staining (n=3). (**D**) Quantitative analysis of the protein expression of C3 in the frontal lobe (n=4). (**E**) Quantitative analysis of the protein expression of CCL3 in the frontal lobe (n=4). (**F**) Quantitative analysis of the protein expression of ZBTB7A, ALDOA, LDHA, PGAM1 in the frontal lobe (n=4). (**G**) Pyruvate content in frontal lobe (n=6). (**H**) NAD^+^/NADH ratio in frontal lobe (n=6). (**I**) Urea content in plasma (n=6). (**J**) Quantitative analysis of the protein expression of OTC in the liver (n=4).Compared with the Con group, **P* < 0.05; Compared with the Rea_H group; the data are expressed as the mean±SD.
